# National character stereotypes mirror language use: A study of Canadian and American tweets

**DOI:** 10.1371/journal.pone.0206188

**Published:** 2018-11-21

**Authors:** Bryor Snefjella, Daniel Schmidtke, Victor Kuperman

**Affiliations:** Department of Linguistics and Languages, McMaster University, Hamilton, Ontario, Canada; University of Texas at Austin, UNITED STATES

## Abstract

National character stereotypes, or beliefs about the personality characteristics of the members of a nation, present a paradox. Such stereotypes have been argued to not be grounded in the actual personality traits of members of nations, yet they are also prolific and reliable. Stereotypes of Canadians and Americans exemplify the paradox; people in both nations strongly believe that the personality profiles of typical Canadians and Americans diverge, yet aggregated self-reports of personality profiles of Canadians and Americans show no reliable differences. We present evidence that the linguistic behavior of nations mirrors national character stereotypes. Utilizing 40 million tweets from the microblogging platform Twitter, in Study 1A we quantify the words and emojis diagnostic of Canadians and Americans. In Study 1B we explore the positivity of national language use. In Studies 2A and 2B, we present the 120 most nationally diagnostic words and emojis of each nation to naive participants, and ask them to assess personality of a hypothetical person who uses either diagnostically Canadian or American words and emojis. Personality profiles derived from the diagnostic words of each nation bear close resemblance to national character stereotypes. We therefore propose that national character stereotypes may be partially grounded in the collective linguistic behaviour of nations.

## Introduction

Stereotypes about national character, i.e., individually held beliefs regarding psychological traits of world nations and cultures, are ubiquitous, stable and influential (see [1]). The importance of these beliefs for the functioning of individuals, groups and societies is hard to overestimate. National character stereotypes have the capacity to fuel discrimination and intergroup conflict [2]; they are also a salient factor in diplomacy [3], governmental and corporate policies [4], marketing [5], and consumer decision-making [6]. The power of stereotypes regarding national character has put them in the center of prolific psychological and social research. A search for topics containing the term “national character” retrieved a total of 551 items in the Web of Science bibliographic database from 1976 till 2015, with an annual linear increase in the number of publications since 2000. Two ways of characterizing beliefs about a nation have reached particular prominence in the literature: an inquiry into personality traits perceived as typical of a nation [7] and an inquiry into perceived positivity of a national culture [8, 9]. The present paper addresses both lines of inquiry. Our interest lies in a question that has puzzled this research field since its inception [10–12]: what are national character stereotypes grounded in?

A simple mechanism for stereotypes to form would be “a statistical agreement between beliefs about a group and the aggregate characteristics of the group in question” [13, p. 831]. If stereotypes are accurate, each culture has a characteristic psychological profile, and individuals are capable—with a degree of precision—of discerning the traits that differentiate this culture from that of other groups. Two observations lend plausibility to this assumption. First, large-scale international surveys show national character stereotypes regarding personality traits to be stable both within individuals and at the aggregated level, as well as within and across cultures [13–18]. For example, when evaluating personality traits of their own cultures, Canadians and Americans demonstrate differences in perceived levels of neuroticism (less typical of Canadians), conscientiousness (more typical of Canadians) and agreeableness (more typical of Canadians) that are strong and consistent over multiple data collection sites in each country [14]. A second reason to expect a high level of accuracy in national character stereotypes is that stereotypes about many other group variables, including age and gender [19–22] show convergence with respective self-reports and observer ratings of psychological traits. It is therefore a tempting explanation that, for example, the reason Canadians are stereotypically more agreeable is that they really are more agreeable.

While attractive as a causal mechanism, simple accuracy of national character stereotypes is questionable. An influential body of negative evidence comes from a series of large-scale studies that focuses on comparing (i) stereotypes about personality traits associated with one’s own and other cultures with (ii) the personality traits of actual individuals from those cultures [13, 14, 18, 23–26]. Based on samples from up to 49 cultures, these studies have addressed (ii) by using the Revised NEO Personality Inventory instrument based on the Five-Factor Model of personality, [27, 28] and have also assessed (i) with the help of the National Character Survey, a short questionnaire designed to quantify stereotypical personality traits of a nation on the same facets as NEO-PI-R (for motivation and reliability, see [14]). Two central claims summarize the findings of these studies: perceived personality profiles vary dramatically from one culture to another, while actual aggregated personality profiles of those cultures show no reliable variability. In other words, when measured using self-reports, national character stereotypes are as consistent and strong as they are inaccurate.

These findings have raised several methodological concerns which question the validity and reliability of intra- and inter-cultural comparison of personality traits using self-reports [29–33]. McCrae et al. [13] address these and other points of methodological concern both by recruiting existing evidence in support of the validity and reliability of their instruments, and by conducting a replication and extension study in 26 cultures. Their recent study reiterates the finding that “[c]onsensual stereotypes of national character are internally consistent, generalizable across raters, and stable over time—but they show only weak traces of accuracy” [13, p. 840]. The broader debate about stereotype accuracy is ongoing [34, 35]. While the debate about the validity of using self-report instruments to make inter-cultural comparisons is not settled, we agree with McCrae et al. [13] that, at present, the more parsimonious interpretation of the available evidence is that aggregated self-reports are a valid method for intercultural comparisons (also see General discussion).

A similar question has been raised about the origin of stereotypes regarding a culture’s positivity. Massive research efforts like the World Happiness Index [36] and the World Values Survey [37] have shown that indices of subjective happiness and life satisfaction aggregated over individuals in a nation correlate strongly with objective measures of national well-being such as the national gross domestic product, social support, life expectancy and others (see also a real-time online happiness index at hedonometer.org [38]). Yet an influential folklore theory of happiness argues that a stereotypical outlook of a culture can diverge from either subjective or objective well-being of individuals from that culture [39–41]. Under this theory, happiness is not “an individual evaluation of life, but […] the reflection of a body of widely held notions about life, that is part of the national character” [39]. The theory proposes that if a culture’s outlook on life is predominantly pessimistic (e.g., as argued for France, Italy and Russia) or optimistic (as argued for the USA), this stereotype would persist despite a change in living conditions and would only be loosely related to subjective happiness of individuals in that culture [9, 42, 43]. There is evidence to suggest that perceived happiness of a culture might indeed be different from subjective happiness of individuals in that culture as well as their objective well-being [39, 44]. If so, stereotypes regarding the optimism or pessimism of a culture’s outlook might be inaccurate, much like the stereotypes about personality traits of a national character.

The central question remains: if it is not the *psychology* of nations, what does give rise to consistent national character stereotypes? In line with prior research [30, 45–47] we speculate that stereotypes about a group (including a nation) may stem from systematic and distinct *behaviors* that this group shows in comparison to other groups. More specifically, beliefs about the psychological profile of a group may partly be rooted in distinct patterns of *language use* that this group produces. As a toy example, suppose that phrases like “hiking”, “camping”, “nature”, “tent”, and “bird watching”” occur with a higher relative frequency in language productions of group A relative to group B. Both members of group A, members of group B, and non-members who are exposed to these language productions might sensibly form a belief that group A prefers outdoors, and are relatively adventurous and sportive in their leisure choices. Importantly, these verbal behavioral markers do not need to stem from respective psychological traits (e.g., adventurousness, or openness to new experiences), or even from relevant non-verbal behavior (e.g., days spent outdoors). A greater verbal emphasis on physically demanding activities or closeness to nature may be a strategy for group A to construct its identity by distinguishing itself from either one specific out-group (as is often done during inter-group conflicts) or from a generic out-group [48–50]. In this case, either a subjective or an objective measure of how adventurous and nature-loving people in groups A and B are will not detect any appreciable group differences in these traits, yet dissimilarities in their verbal behavior may give rise to different beliefs regarding psychological profiles of these groups. In sum, a stereotype of group A as an outdoorsy, hardened collective would be an accurate reflection of its observable verbal behavior, and it may form regardless of the ground truth of that group’s personality traits. That is, a nation’s persona may differ from the nation’s personality.

Determining whether this process of verbal behaviour influencing stereotypes has indeed occurred in the formation of national character stereotypes is challenging. It is possible that both differences in national language use and national character stereotypes are caused by some other factor (such as they are both a result, but not a cause, of each nation’s ethos). It seems unlikely that language use would be the sole cause of national character stereotypes, given the possibility of non-verbal behaviours and other mediums of transmission (such as visual media) contributing to national character stereotypes. We conduct an observational study of national language use, but do not determine if language use is a causal factor behind national character stereotypes. What we can establish is three things. First, whether the language use of each nation has systematic differences. Second, whether those differences in language use a sufficient basis to perform differing personality judgments. Third, whether the personality judgments derived from national language use are similar to other measurements of national character stereotypes or aggregated self-reports of personality traits.

We establish differences in national language use by examining linguistic productions by individuals from the United States and Canada in the micro-blog platform Twitter (see below for the motivation of our choice). Our goal is to establish diagnostic patterns of language use associated with the two cultures under comparison, quantify what personality traits and outlook these patterns convey, and ultimately test whether the national personality profiles that emerge from Canadian and American preferences in language use correspond to independent assessments of their respective national character stereotypes. We predict that linguistic biases of a nation are consistent with its national character stereotype but do not correlate with the results from self-report psychological tests.

This paper adopts an open-vocabulary approach [51–53], which predicts psychological characteristics of interest (in our case, selected aspects of national character) from distributional patterns in lexical choices that individuals and groups make in their natural language productions. The open vocabulary approach is a bottom-up, data-driven technique which provides inferential estimates as to how diagnostic each linguistic unit is of those characteristics. It generates specialized lexica where each word is assigned a weight showing how strongly and with what polarity a word reflects that characteristic: for instance, the words *bored, annoying*, *lonely* are associated with high neuroticism, whereas *success, workout* and *praise* are associated with lower neuroticism [51]. Available open-vocabulary analyses of social media (typically, Twitter or Facebook) have revealed reliable and face-valid differences in language use as a function of gender, age, type of personality, temporal orientation, occupation, region of origin or residence, political orientation and multiple other characteristics [51–61] Moreover, these patterns of preference show a higher-than-human-rater accuracy when predicting respective characteristics of “unseen” users, i.e. users not included in the dataset from which linguistic patterns were derived [51, 57, 59]. One study has used the open vocabulary approach to study age, gender, education, and political stereotype accuracy. [62] The accuracy of a stereotype was gauged through comparison of the linguistic features which are correlated with ground truth of Twitter users’ traits to features perceived to indicate these traits. Our study is similar, in that we are comparing the ground-truth of Canadian and American language use to other research which has established the perceived national character stereotypes of Canadians and Americans.

The novelty of the present paper is in the psychological phenomenon that it approaches: national character stereotypes and verbal behavior of nations. The logic of the study is as follows. We use tools of the open-vocabulary approach to identify linguistic units (words, phrases, emoticons and emojis) that are most specific to American and to Canadian users of Twitter, as well as to subsets of users living in two smaller regions straddling the border of these two countries (the Great Lakes regions and the West Coast region). We further quantify the level of positivity and the personality profile associated with the “most American” and “most Canadian” linguistic units. First, we resort to specialized lexica of positivity to assess characteristics of the verbal output of each nation. Second, we administer a modified version of the National Character Survey [14], which asks participants to evaluate a personality of an individual whose linguistic preferences contain units preferred by Americans and, separately, by Canadians. This enables us to obtain language-based psychological profiles of the two nations, i.e., personas of a typical American and Canadian based solely on their differential language use. Finally, we pit the results of those comparisons against available independent assessments of stereotypes to evaluate the alignment between language-based patterns and findings from the National Character Survey of Americans and Canadians [14].

Before presenting our study, we discuss implications of two methodological choices that we made: the use of a social media outlet like Twitter as a source of verbal behavior that may mirror beliefs about nations, and the focus of Canada and the US as our specific test case.

### 0.1 Verbal behavior in social media

Recent proliferation of electronic communication enables us to tap into billions of natural language productions, of which a substantial percentage can be enriched with information about the origin of the speaker and the geographic location of the production. Our study contributes to the rapidly growing body of social science research that demonstrates a link between language behavior and geographic, demographic, social or psychological characteristics of individuals or groups [38, 51, 63, 64]. The empirical base for our study is a dataset of approximately 50 million posts gathered over the year 2015 from Twitter, a microblog and an electronic communication platform, which enables individuals to publish messages restricted to 140 characters in length. For a short introduction to conducting psychological research on Twitter, including data collection and recent findings see [65]. Twitter is one of the 10 most visited sites on the internet (see http://www.alexa.com/siteinfo/twitter.com), with 310 million active monthly users (see https://about.twitter.com/company) reported by the company. An independent estimate is that approximately 21% of all adults in the US used Twitter in 2016 [66]. Advantages of analyzing outputs of electronic means of communication like Twitter are well known: one gains access to millions of observations from thousands of language speakers varying in gender, age, language knowledge, socioeconomic status, psychological traits, indices of well-being and happiness, and place of residence. Although Twitter is not a representative sample of the population [67] it provides a larger, more natural and diverse sample than many psychological studies employ. Approximately 1% of tweets are tagged with GPS coordinates, enabling one to identify the location of a tweet production with a precision within a few meters. We only considered these geo-tagged tweets to enable attribution of a language production to a region and a country.

Tweets are samples of natural language use and thus are free of potential biases that experimental methods of stereotype elicitation gives rise to [29–31]. We make no pre-selection of the topic that a tweet discusses: much more often than not, tweets in our pool did not specifically discuss the sender’s attitudes and beliefs about her own and other nations. Thus, statistical patterns of word occurrence extracted from tweets are indicative of broadly construed verbal behavior of a nation rather of specific linguistic patterns associated with a topic of national stereotypes.

Furthermore, we only considered public Twitter messages here. These can be directed to a specific person or group, or—more often—have no addressee, but importantly, they can be read by anyone with internet access. Tweets produced by representatives of one culture, e.g., Americans, are equally visible to fellow Americans, Canadians, Chileans, or Ghanaians, and vice versa. This transparency may partially answer why stereotypes about Americans (or any other nation) are relatively stable around the world [14], even though immediate exposure to and knowledge of psychological traits of Americans would clearly vary from one country to another [15]. Linguistic outputs of nations are arguably more accessible around the world (through in the past TV, radio, newspapers, and contemporarily on Internet sources and social media) than their non-verbal behavior or psychology, raising the level of mutual familiarity even between nations that are geographically remote. This is certainly true of widely accessible sources like Twitter.

We do not argue, of course, that only users of Twitter have access to the linguistic foundation of stereotypical beliefs, or even that such users can always differentiate between tweets sent by Americans, Canadians or say Brits. In practice, identifying a tweet with a culture of a person who sent it is only feasible if the sender revealed her affiliation with a country through either the content of the tweet message, linguistic cues like a regional dialect, an explicit mention of the location in the user profile or a profile picture. This limits the pool of messages relevant for national character stereotypes considerably. Yet prior work shows that even small subsets of tweets and users are representative of their communities, because they tend to share the same culture (including beliefs and attitudes), as well as environmental and social resources and affordances [51]. Thus, it is possible that readers of tweets and similar media might form their beliefs regarding nations based on a fraction of users and messages whose national affiliation is clearly marked. Also, the constructed national character channeled through tweets is likely to be similar to the character conveyed through other media, and so we use Twitter data to approximate linguistic choices that a nation would reveal in other accessible outlets too.

### 0.2 Geographic choices

Since we argue that the nation’s persona is constructed in opposition to a (specific or generic) out-group, we do not consider language output of a country in isolation, but in comparison against that of another country. Canada and the United States, and their national character stereotypes, are the exemplar of the controversy over whether national character stereotypes are accurate [68–77]. Canadians and Americans do not reliably differ in their personality traits as measured with the NEO-PI-R self-report instrument [14]. In Terracciano et al. [14] NEO-PI-R scores are standardized to have a mean of 50 and a standard deviation of 10. In this study, at the facet level estimated differences between Canadians and Americans are less than 4, with 27 of 30 facets below 2, and differences in the factor scores are 2 or below. Furthermore, Canadians and Americans cluster together when compared to personality traits of other nations [14, 78]. Yet, one finds marked differences in both their auto-stereotypes (what in-group members believe to be true) and hetero-stereotypes (what out-group members believe to be true). For instance, the National Character Survey [14] revealed that Canadians evaluate themselves with a substantially higher level of agreeableness and lower level of neuroticism than the levels emerging in self-evaluation by Americans. These self-evaluations are virtually identical across three testing sites in Canada and four in the US. Another dataset [79] comes from a study, in which individuals from 9 countries evaluated the typical citizens of 5 English-speaking countries. Similarly, the results show that Americans perceived themselves as open-minded and modern, but also as relatively unfriendly, selfish, impolite, and aggressive. They have believed that Canadians were more friendly. In turn, “[t]he Canadian participants had a fairly positive autostereotype, perceiving themselves as the least aggressive, most open-minded, and second most friendly (behind Australians) of the stimulus countries. They also had an extremely negative hetero-stereotype of Americans, whom they perceived as the most aggressive, most close-minded, most selfish, most patriotic, least religious, least friendly, and least polite of all of the stimulus countries.” [79]. Thus, Canada and USA exemplify the paradoxical case whereby nation-wide beliefs about one’s own psychological traits are both stable within the nation and confirmed by beliefs of the neighbor (e.g., Americans and Canadians both believe that Americans are relatively unfriendly, selfish, aggressive, and impolite), and yet these beliefs find no support in self-report personality assessments of individuals from those countries.

In sum, the present paper aims at finding diagnostic patterns of language use associated with the American and Canadian cultures. Study 1A presents a corpus analysis which identifies those patterns based on Twitter data from the entire territories of the two countries. In Study 1B, we use specialized lexica to learn what kind of outlook is signaled by preferential patterns of language use in the two countries. Studies 2A and 2B conduct a survey of the personality traits that human judges associate with linguistic patterns over-represented in the Canadian and American language use. We test whether these patterns correlate with independent estimates of beliefs about cultures (i.e. national character stereotypes) and estimates of psychological traits in those nations. Finding correlations between linguistic choices and stereotypes, but not between those choices and self-reports, would imply that (a) a multitude of linguistic productions by a national culture can form a sufficiently coherent psychological profile, which (b) is successfully perceived by members of other cultures, (c) can form a basis for sufficiently stable stereotypes regarding that culture, and (d) can even do so in the absence of systematic cross-cultural differences in self-report measured psychological traits. The supplementary materials S2 and S3 Files contain two further studies, showing the patterns observed nationally hold of two border regions of each country.

## 1 Study 1A: Language patterns of Canada versus USA

This study examines the large scale differences in linguistic productions between the USA and Canada. Our linguistic analysis further imposes the requirement that populations use the same language, English, across the national border (minus possible dialectal or regional differences). Thus we pass no judgment on an intriguing possibility that non-English speaking populations in both countries (including Quebec and other francophone areas in Canada [80]) constitute separate nations, with their own auto- and hetero-stereotypes of national character.

All studies were approved by McMaster Research Ethics Board, protocol number #2011-165, titled “Research program of the eye-tracking lab at the Department of Linguistics and Languages.”

### 1.1 Twitter data collection and filtering

Tweets were collected from the Twitter “garden hose” Streaming API(available at https://dev.twitter.com/streaming/overview) which allows queries to 1% of recent tweets. We collected tweets marked with latitude and longitude coordinates using the *streamR* package [81] in the *R* statistical computing environment [82]. Data collection ran from February 12, 2015 to February 14, 2016. Data collection complied with the Twitter steaming API’s terms of service.

For the present paper our criteria for including tweets in our analyses were as follows: The tweet was tagged by Twitter’s automatic language recognition as being written in English; the language in the tweet matched the user’s self-announced default language (i.e., only English tweets from US and Canadian speakers); the tweet included latitude and longitude coordinates indicating where it was sent from; the tweet was sent from within a prespecified set of coordinates; the tweet user must have sent more than 10 tweets; and the tweet was not sent by a bot (see below).

Bots are applications that automatically perform tasks like reporting the weather or traffic conditions, advertising businesses and others. It is possible to detect bots using natural language processing methods purely from Tweet content [83]. To clean bot accounts in the data, we used two of the three methods presented in [83]: average pairwise tweet dissimilarity and average URLs per tweet. Average pairwise tweet dissimilarity compares the average dissimilarity between the longest common substring of pairs of a sample of a user’s tweets; bots tend to send tweets with highly similar common substrings. We calculated this measure for all users, using a random sample of up to 500 tweets per user. According to these metrics, the following tweet is likely from a bot

Details #gentleman #dandy #menwear #instafashion #fashion #instagramers #lookoftheday… [URL]

However the following tweet is likely from a human:

This girl sent me a text bout happy father’s day. I’m like.… who dis?

We removed all users with an average tweet dissimilarity of less than.8. Bots also tend to send tweets with many URLs. We excluded any user who had an average of 1 or more URLs in a random sample of 500 of their tweets.

#### 1.1.1 Language filters

We defined what linguistic features to count for our analysis as follows. Psychological characteristics like attitude, mood, and sentiment are often expressed on social media using emoticons, i.e., combinations of keyboard characters used to denote a facial expression, or emojis, i.e., Unicode characters containing a standardized set of pictographs [84]. We included both unigrams (individual words, emoticons and emojis) and bigrams (contiguous pairs of words, emoticons and emojis) in our analysis. Specifically, we defined a unigram as any emoji character, any sequence of punctuation and characters matching an emoticon, or any non-URL sequence of two or more alphanumeric characters or hyphens and underscores, minus what we filter out below. All tweets were tokenized, i.e., divided into individual unigrams and bigrams, and the frequency of each unigram and bigram was calculated. To this end, we included a dictionary of emoticons (Accessed at http://people.few.eur.nl/hogenboom/files/EmoticonSentimentLexicon.zip) in our tokenizer, as well as considered emojis alongside words.

We removed from the data all unigrams and bigrams containing hashtags (e.g., #scientistproblems), Twitter usernames (e.g., @user), and URLs, as well as tokens with a frequency of 2 or less, non-emoji tokens containing only a single character, punctuation and sequences of punctuation not matching an emoticon, and function words using stoplists from the *tm* package for English [85]. We standardized spellings between English-speaking nations, (e.g., “centre” and “center”) based on lists of spelling variants collected at http://www.tysto.com/uk-us-spelling-list.html.

One issue is that the most diagnostic words of a geographic region (i.e., statistically over-represented in that region) are often words denoting locations in those regions. That Canadians say “Toronto” more than Americans is neither interesting nor surprising. To cope with this, we removed geographic names originating from the countries of interest from the data, based on the free gazetteer database (www.geonames.org). Geographic names were removed, unless those geographic names were entirely composed of words from a list of common words for that language (e.g., occurrences of “River Bend”, a common place name, or “Hell”, the name of a location in Michigan, would not be removed from the data, but “Greater London” or “Toronto” would be). The end result of this data processing stage was a list of all unigrams and bigrams with their frequency of occurrence in tweets sent from Canada and, separately, those sent from the US.

#### 1.1.2 The open-vocabulary method: Statistical considerations

Our next step was to identify unigrams and bigrams that are statistically over-represented from one country relative to another. Based on the frequency of unigrams and bigrams in their respective national sources, we implemented the log-odds ratio informative Dirichlet prior (LORIDP) method [86, 87]. The LORIDP method was originally proposed in order to find words that are statistically over-represented in one text document as compared to another, and vice versa. It has the advantage of detecting differences in high frequency words, while not overemphasizing differences in rare words, a common problem for measures of effect sizes used to compare word frequency in corpora (see [87] for an extensive discussion). In the present study, the two documents under comparison are the words (unigrams) and bigrams produced by speakers from two different countries (or smaller regions within each country). To illustrate its application to Canadian versus American language usage, the LORIDP method estimated the difference between the frequency of each word or bigram *w* in the Canadian (*i*) and US (*j*) parts of the Twitter corpus via the log-odds ratio for *w*, $\delta_{w}^{\left(i-j\right)}$, which is computed as

$$\begin{array}{c} \delta_{w}^{\left(i-j\right)}=log(\frac{y_{w}^{i}+\alpha_{w}}{n_{i}+\alpha_{0}-\left(y_{w}^{i}+\alpha_{w}\right)})-log(\frac{y_{w}^{j}+\alpha_{w}}{n_{j}+\alpha_{0}-\left(y_{w}^{j}+\alpha_{w}\right)}), \end{array}$$

where in this case, *n*_*i*_ is the total number of words and bigrams in the Canadian part of the Twitter corpus *i*, *n*_*j*_ is the total number of words and bigrams in the US part of the Twitter corpus *j*, $y_{w}^{i}$ is the frequency count of word or bigram *w* in corpus *i*, $y_{w}^{j}$ is the frequency count of the word or bigram *w* in corpus *j*, *α*_0_ is the total number of tokens in the combined Canadian and US parts of the Twitter corpus and *α*_*w*_ is the total frequency of the word or bigram *w* in the combined Canadian and US parts of the Twitter corpus. The variance of the above measure was then estimated as

$$\begin{array}{c} \sigma^{2}(\delta_{w}^{\left(i-j\right)})\approx \frac{1}{y_{w}^{i}+\alpha_{w}}+\frac{1}{y_{w}^{j}+\alpha_{w}}, \end{array}$$

Next, the *z*-score statistic of the LORIDP of each noun was calculated as

$$\begin{array}{c} \frac{\delta_{w}^{\left(i-j\right)}}{\sqrt{\sigma^{2}\left(\delta_{w}^{\left(i-j\right)}\right)}}. \end{array}$$

The resulting z-score can be used to select which words are significantly over/under represented in the corpus, based on traditional thresholds of statistical significance (i.e. z-scores exceeding ± 1.96). Due to the large number of comparisons (2,761,118 words/emojis/emoticons), we apply the Bonferroni correction to control for the inflated Type I error rate which originates from considering multiple comparisons: we removed all items with *z*-scores below the corrected threshold of *p* = 0.01 (|*z*| < 5.9).

The resulting range of LORIDP *z*-scores represents a continuous standardized measure of the overall divergence in single word or emoji (unigram) and bigram usage between Canadian and US Twitter users. To revert to the above demonstration of the LORIDP’s application to Canadian versus US language usage, a unit associated with a negative value indicates a degree of ‘over-representation’ in Canada, and a unit associated with a positive value indicates a degree of ‘over-representation’ in the US. If a word or bigram is equally well represented in both the US and Canada, the formula yields a *z*-score of exactly 0.00. To illustrate, the word “great” has the most negative *z*-score (*z* = -89.72) and thus indicates that the noun is significantly over-represented in Canada, compared to the US. Conversely, the word “shit” lies at the opposite end of this continuum, with the most positive *z* score (*z* = 104.34), which signifies that it is over-represented in the US.

It is common practice to compare word use between two corpora by treating the statistical analysis as a classification problem (i.e. predicting whether a tweet originates from Canada or the United states based on the words in the Tweet). Oft-used methods include ridge or other regularized regression or support vector machines. However, such methods have been argued to be conceptually problematic [87, 88] for language data. Someone’s traits (such as their nationality or personality) are not plausibly a function of the words they use; rather the words someone uses are a function of their traits. As a reductio ad absurdum, upon finishing writing this paper we did not discover that we are Canadian or British or introverted or neurotic, yet this is how a classifier would treat our language generation process. The conceptual strength of LORIDP is also complemented by its ease of computation and statistical properties. By employing shrinkage to the log-odds-ratio based on overall corpus size and frequencies from a background corpus, LORIDP scores are free from disproportional influence from very high and very low frequency words. Typically before classifiers are trained, various transformations, filters, and normalizations of word-frequencies are employed to correct for word-frequency distributions. LORIDP achieves this in a principled and easy to compute fashion. Supplementary material S4 File contains a simulation to provide further illustration of the strengths of the LORIDP statistic.

### 1.2 Results and discussion

**Fig 1:**
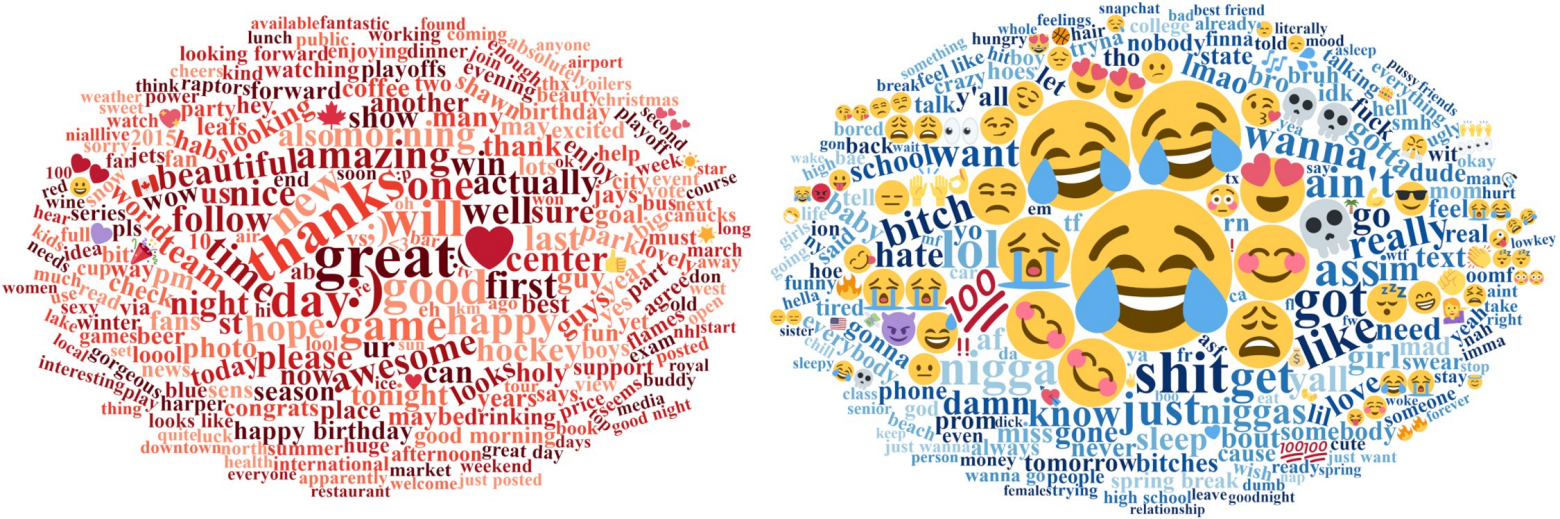
250 most Canadian and American words. The top 250 US and Canadian words. Text size is proportional to LORIDP *z*-score. Color is for readability only.

We collected 44,405,347 tweets, of which 37,066,693 yielded usable tokens after our filters were applied. 6061 words passed the critical Bonferroni-corrected *z*-score at the lower tail of the LORIDP distribution, i.e. words over-represented in Canadian tweets. 3393 words passed the critical Bonferonni-corrected *z*-score at the upper tail of the LORIDP distribution, i.e. words over-represented in US tweets. Fig 1 presents wordclouds of Canadian (red) and American (blue) words, with size of the word corresponding to its absolute LORIDP z-score. A full list of these words, including LORIDP score and frequency can be found in the supplementary materials S5 File.

Before exploring the nationally diagnostic language, it is important to note that the vast majority (99.66%) of words, emoticons, and emojis on Twitter are not used in a reliably different way between Canada and the US in our analysis. This leads to a critical caveat to our argument. We are not claiming that language use between Canada and the US is wildly divergent. Rather, a minority of words show differences in relative use, and it is these words with the most extreme difference in relative use that mirror national character stereotypes. Importantly, there is no statistical reason why words selected using our procedure would reflect differences in personality traits, positivity or any other psychological aspect. We select words solely based on their frequency of use. With these important caveats in mind, we discuss what the differences are in the most nationally diagnostic words.

While a comprehensive linguistic analysis of dialectal differences between Canada and the US is a subject of future research (for relevant dialectological work see [89–91], we highlight cross-linguistic patterns relevant for our study. One distinguishing feature of American lexical choices relative to Canadian is an over-representation of several types of non-standard language. For instance, American tweets tend to use slang, primarily netspeak (i.e., specialized slang developed for electronic communication): lol [laughing out loud], lmao [laughing my ass off], as well as af [as f*ck], rn [right now], tf [the f*ck], idk [I don’t know], ion [I don’t]. Canadians more often used forms like ur [your] and pls [please]. Similarly, American tweets contain a substantially greater number of dialectal forms: e.g., ain’t, wanna, yall, bout, yo, y’all, bro, lil, gonna, bruh, tryna, finna, and hella. Some of these forms tend to be relatively localized (e.g., yall, y’all and ion are mostly found in Southeastern states [89]), while others (bro, lil) show a broader geographic spread over the US in our data, with a preferential occurrence in large metropolitan areas. Also, there is a relative abundance of emojis (pictographs conveying emotional states) in the American data (e.g., 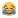, 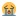), while Canadians favor a small number of emoticons, such as :), ;) and :(. Conversely, no emoticons are found among very American outputs, and only few emojis (“purple heart” 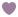 and “maple leaf” 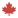) are found among Canadian outputs. A preference of emojis over emoticons or vice versa is in line with the findings of [84], who report an increase in the use of emojis to correlate with a decrease in the use of all other non-standard forms, including emoticons. Finally, American lexical choices show a clear relative preference for taboo words, including swear words, expletives, and racial slurs (e.g., f*ck, sh*t, ass, hoe, b*tch, n*gga).

In sum, we observed a tendency for Canadians to prefer standard verbal output and generally eschew non-standard language and non-linguistic means of visual communication. At the same time, Americans showed a more diverse use of registers and dialects of the English language, as well as emojis. Greater fluency in the use of taboo words correlates with such personality traits as openness and neuroticism [92]. We argue, and demonstrate in what follows, that this increased resourcefulness in language use, and reliance on non-standard language, is indeed indicative of a higher level of openness and neuroticism characteristic of Americans as compared to Canadians.

**Fig 2:**
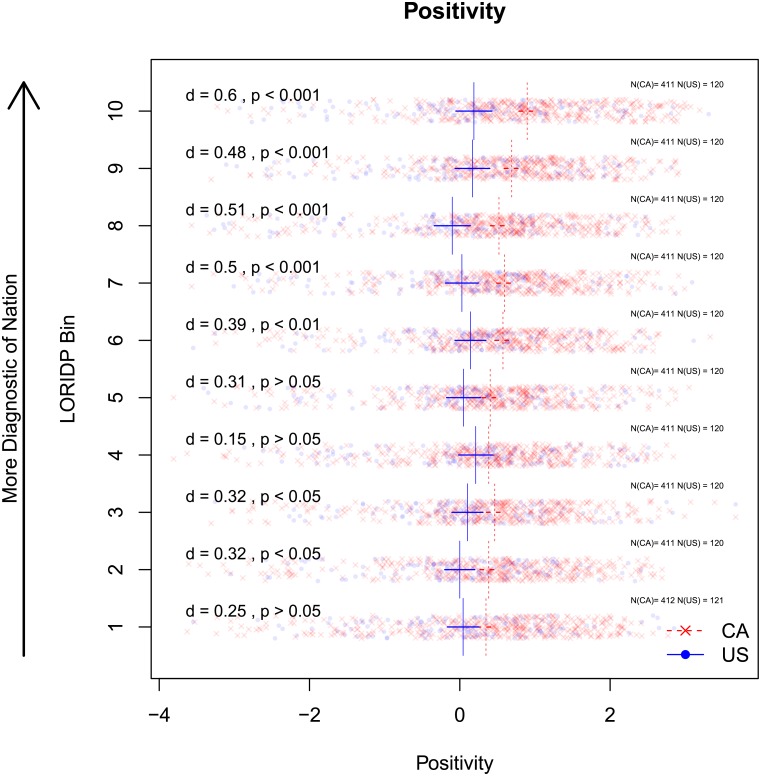
Positivity of Canadian and American words by LORIDP bin. Relative Positivity of very American (blue) versus very Canadian (red) words. Vertical lines indicate mean positivity of American and Canadian words in each bin. Horizontal lines are 95% confidence intervals of the means. Cohen’s d and p-values for t-tests within each bin are reported in the left of the fig. Canadian words are generally more positive across the LORIDP distribution, with the greatest and most robust difference amongst the most nationally diagnostic words.

**Fig 3:**
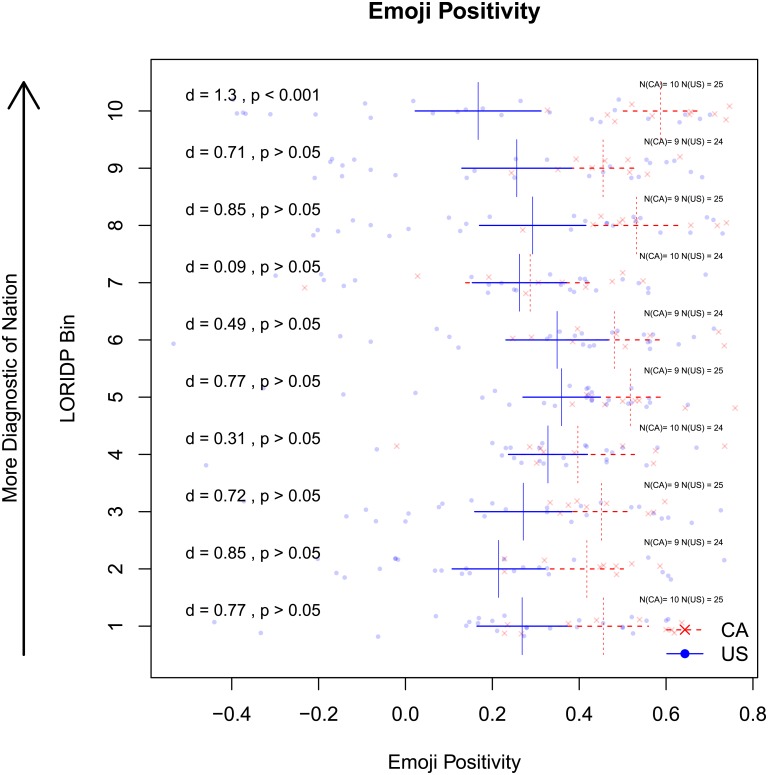
Positivity of Canadian and American emojis by LORIDP bin. Relative Positivity of very American (blue) versus very Canadian (red) emojis. Vertical lines indicate mean positivity of American and Canadian words in each bin. Horizontal lines are 95% confidence intervals of the means. Cohen’s d and p-values for t-tests within each bin are reported in the left of the fig. Canadian emojis are consistently more positive across the LORIDP distribution, but not reliably.

Another, more critical difference is a clear prevalence of negative outputs in American tweets over Canadians. This trend is obvious both in words and phrases denoting emotional states (see Fig 2. American: hate, love, miss, mad, feel, swear, tired; Canadian: great, thanks, good, amazing, happy) and in emojis and emoticons (see Fig 3 below). The next section quantifies these tendencies and examines whether they contribute to the stereotypes about optimism in a nation’s outlook. As mentioned in the Introduction, we use two methods to this end: a corpus study of specialized lexica (Study 1B) and a personality survey with human participants (Study 2A and 2B). We describe the findings of both methods in turn.

### 1.3 Study 1B

In this series of analyses, we examine the relationship between how diagnostic a linguistic unit is of one country relative to another and how indicative this unit is of perceived optimism. Since optimism is a backbone of cultural norms of a nation (see the Introduction), it is important to examine cross-national differences in optimism and their psychological reality. Whether or not different measures of a nation’s positivity align sheds light on the core question that we posited above: what are stereotypes grounded in? While the LORIDP measure reveals which words are preferred by each nation, we require a means to quantify the emotional positivity of words and emojis. Estimates for positivity of words are taken from two datasets. [93] collected positivity ratings for 13,000 English lemmas (citation forms of words, i.e. without affixes), from human participants on the crowdsourcing platform Amazon Mechanical Turk. Ratings for these lemmas were expanded to word forms, providing close to 23,000 positivity-rated words. [38] collected ratings for 10,000 words frequent within multiple English corpora. Positivity, in both these datasets, is measured on a 1-9 scale, from negative to positive. Combining both datasets yields 32,186 words rated for positivity. Positivity of emojis was assessed by [94] who used a supervised machine learning approach to Twitter data in 13 languages. Novak et al.’s machine learning algorithm predicted whether a tweet was positive, negative, or neutral in sentiment. An emoji’s positivity was defined as the proportion of positive tweets it occurs in minus the proportion of negative tweets it occurs in. Emojis scores range from −1 to +1, where −1 would indicate an emoji always occuring in negative tweets and +1 an emoji only occuring in positive tweets. In what follows, we consider both words and emojis: Emojis allow for conceptual replication of results of the positivity of words.

In order to compare the positivity conveyed by the most diagnostic Canadian and American words, we aggregated all Canadian and American words, separately, into 10 bins, based on the order-ranked LORIDP score in each country. In other words, we compare the top 1-10% most Canadian against the top 1-10% most American words, the 11-20% most Canadian against the 11-20% most American etc. In each bin and for each country, we associate words (and for positivity, also emojis) with their positivity, where such scores are available.

To facilitate comparison of very Canadian and very American words, Figs 2 and 3 include standardized effect sizes (Cohen’s d) and results of t-tests (Bonferroni-corrected for 10 comparisons for each dimension of personality) in each bin. Fig 3 reports a comparison of positivity of emojis. The supplementary materials S1 File include wordclouds that report the words with the highest and lowest levels of positivity (S1 File Fig A) within each bin of rank-ordered LORIDP scores.

#### 1.3.1 Positivity

Fig 2 presents the positivity/negativity of bins of Canadian and American words ranked by LORIDP. Canadian words are consistently more positive across the LORIDP ranks, with the greatest difference in the most nationally diagnostic words (top 4 LORIDP bins). The difference between the most diagnostic words of Canadians and Americans is medium sized by Cohen’s criteria (d = 0.6, p < 0.001). Similarly, the positivity of emojis diverges somewhat between nations with a large difference (d = 1.3, p < 0.001) amongst the most nationally diagnostic words. American emojis are overall more negative than Canadian emojis in lower bins, but the differences are much less reliable, see Fig 3.

In sum, distinctively Canadian word use does seem to be more positive, and unlike national character stereotypes, this difference appears to be rooted in psychological reality. Independent evidence confirming our assessments that Canadians are overall happier than Americans comes from the 2005-2009 World Values Survey Wave 6 [37], i.e., the most recent dataset to include our two target countries. This dataset demonstrates that the subjective level of happiness is substantially higher in Canadians compared to Americans (46.4% vs 34.4% are very happy, 49.2% vs 58.8% quite happy, 3.8% vs 6.4% not very happy and 0.6% vs 0.4% very unhappy). This advantage held across age and gender groups. It appears that, unlike in the case of personality traits (see Introduction), the stereotype of a happy Canadian is grounded in the reality of Canadians feeling more happy than their southern neighbors, and these differing levels of happiness also emerge in lexical preferences of the two countries. To a degree, our findings run counter the literature that advocates a predominantly positive outlook among Americans [39, 44], and highlights the importance of comparative cross-national studies of subjective and objective optimism. We expand this analysis over individual personality traits, using a lexicon of words associated with personality traits on Facebook from [51] in the Supplementary Materials S1 File.

## 2 Study 2A: Human ratings of diagnostic words

Thus far we have identified words and phrases that are most characteristic of tweets authored by Canadians and Americans. We have also demonstrated that these linguistic choices tend to be associated with different levels of positivity. But can these choices lead a reader of social media to form a recognizable belief regarding a character who uses this language? Also would these beliefs be stable enough between readers to form a coherent stereotype that holds in a community? We took a step towards answering these questions by conducting a version of the National Character Survey. [14] Specifically, we presented participants with either the most characteristic Canadian or US linguistic choices and asked them to evaluate the personality traits of a fictitious person whose speech is best characterized by those sets of linguistic items. We further correlated language-based judgments of personality traits of a fictitious Canadian and American with independent assessments of the national character stereotypes of those cultures from [14]. If language behaviour mirrors national character stereotypes, we expect a reliable correlation between the two assessments of perceived personality traits.

### 2.1 Method

The following study was approved by McMaster Research Ethics Board, protocol number #2011-165, titled “Research program of the eye-tracking lab at the Department of Linguistics and Languages.”

#### 2.1.1 Participants

Two experiments were conducted using the online crowdsourcing Amazon Mechanical Turk platform (mturk.com), one presenting participants with characteristic US words and another with characteristic Canadian words, henceforth Exp-Canada and Exp-US. Data collection occured twice, once in 2016 and once in 2018. 200 participants with the IP addresses based in the USA or Canada were recruited for each experiment: mean age in Exp-US was 38 years old (SD = 12.2; ages of 2 participants were unreported; 105 females) and in Exp-Canada it was 38 (SD = 11.55; 5 ages unreported; 110 females). Participants only took part in one of the experiments and each received a monetary compensation of 1 USD.

#### 2.1.2 Materials

We identified words with the most negative (Canadian) and most positive (US) z-scores, as estimated by the LORIDP measure. In line with Schwartz et al. (2013), we removed from this list all inflections and derivations of “f*ck” as well as “n*gger”: these taboo words tend to overshadow the impact of the less emotionally charged lexicon. Then we selected 120 words from either end of the distribution (see Table 1 for Canadian words and Table 2 for American ones) with the greatest absolute z-scores and presented the two lists separately for evaluation in Exp-US and Exp-Canada, respectively.

**Table 1:** 120 words most characteristic of Canadian tweets.

1	2	3	4	5	6
great	:)	thanks	will	good	day
new	game	one	time	amazing	happy
well	first	hope	awesome	morning	nice
follow	center	actually	last	beautiful	please
win	team	hockey	sure	;)	also
can	st	ur	us	show	thank
guy	guys	now	photo	looks	looking
many	another	pm	park	season	today
wow	fans	world	:(	holy	best
year	habs	may	place	goal	check
coffee	maybe	happy birthday	years	enjoy	forward
shawn	jays	leafs	congrats	fun	beer
lots	way	yes	sens	hey	playoffs
boys	pls	birthday	part	fan	excited
drinking	via	ab	big	vs	loool
watching	bit	city	support	lovely	two
yet	party	says	news	good morning	end
help	jets	evening	series	10	agree
winter	blue	2015	bus	must	flames
read	huge	snow	harper	eh	cup

120 words most characteristic of Canadian tweets, in a descending order of their absolute z-score values.

**Table 2:** 120 words most characteristic of American tweets.

1	2	3	4	5	6
shit	lol	got	ass	like	get
just	ain’t	wanna	bitch	want	really
know	go	yall	hate	im	damn
girl	sleep	gotta	need	love	lmao
af	gone	school	text	baby	bout
miss	yo	y’all	bro	let	never
mad	bitches	lil	gonna	rn	somebody
feel	dude	prom	tho	tomorrow	real
phone	state	tf	idk	hoes	cause
nobody	talk	everybody	said	mom	tell
bruh	swear	always	tired	back	tryna
spring break	hell	crazy	boy	smh	hoe
wit	god	ion	yeah	talking	asf
oomf	finna	ya	even	feel like	funny
someone	wish	people	college	ny	fr
car	bae	bored	ugly	told	money
everything	hair	ready	beach	cute	hit
wanna go	life	nah	ca	already	stay
man	em	okay	aint	da	take
imma	hella	high school	going	high	just wanna

120 words most characteristic of American tweets, in a descending order of their absolute z-score values.

We adapted the National Character Survey [14] (henceforth, NCS) to interrogate one’s opinions regarding the (hypothetical) speaker with a given set of linguistic preferences. The NCS is a brief instrument designed to evaluate subjective beliefs regarding personality traits prevalent in a certain culture and has been widely used in research of national stereotypes (see Introduction for references). The most important deviation we made from the NCS is that our evaluation aimed at a hypothetical person rather than a culture: accordingly, no mention of a country or a culture has been made at the time the key evaluations were made (see below). The original NCS (and our adaptation) consists of 30 scales, with 6 scales representing one of the Big Five personality traits. [14] Each scale is a pair of contrastive verbal descriptions (e.g., *Anxious, nervous, worrying* vs *At ease, calm, relaxed*) and a sequence of five radio buttons. Selection of a radio button closer to one definition or equally distant from both enables us to quantify opinions about the person behind the linguistic choices.

### 2.2 Procedure

We used the option provided by the Amazon Mechanical Turk to mark these experiments as a project that “may contain potentially explicit or offensive content, for example, nudity.” Participants who chose to sign up for the task first read the letter of information and the following instructions:

You are invited to take part in the study that is investigating how a person’s language use reflects his or her personality. We are interested in what impression you get about a person when you read words that that person uses much more often than other people. We have prepared for you a list of 120 words that are most characteristic of a certain person’s language. The top rows in the list are especially reflective of that person’s language, while rows in the bottom are somewhat less reflective. Your job is to read these words and come up with an impression of character traits of an individual who produced them.

Now use scales below to communicate your opinion of the person who often produces the words above. Fill the circle that is the closest to the description that you think fits that individual’s personality.

Then participants saw a list of 120 words organized in the descending order of the absolute z-scores, from the more to the less characteristic words and phrases for one of the nations under comparison, see Tables 1 and 2. They were then presented with 30 scales from the National Character Survey (Terracciano et al., 2005) and were instructed to fill in circles closer to the definitions that they see fitting.

Finally, we presented them with two free-form questions marked as optional: “Does this person’s language remind you of any nationality (e.g., German, Chinese, etc.)?” and “Do you have any other comments about this person?” These questions were not visible until after the survey scales were completed.

Participants were additionally asked to provide basic demographic information (age, gender, where they lived till the age of 7, and their native languages). The entire experiment took no more than 20 minutes.

### 2.3 Results and discussion

7 participants in Exp-Canada and 5 in Exp-US did not provide responses or provided responses in an unrealistically short time (less than 1 minute), and were excluded from further consideration. We also excluded 170 responses in which a selection on a given scale was not provided. The resulting pool consisted of 5,648 data points for Exp-Canada and 5,822 for Exp-US.

**Fig 4:**
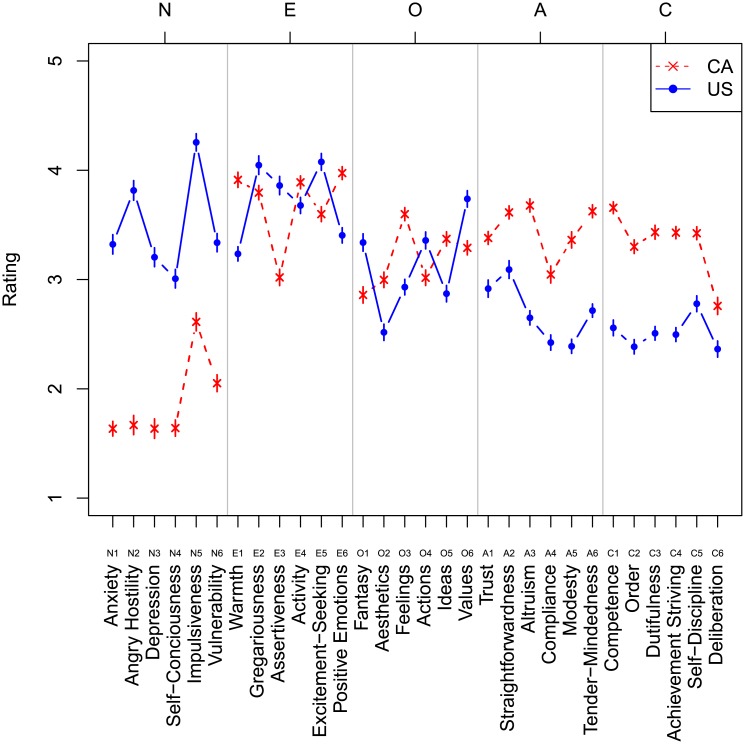
Mean ratings for 30 scales of the modified National Character Survey. Mean ratings for 30 scales of the modified National Character Survey, based on words characteristics of the US (blue) and Canada (red). Error bars stand for 1 standard error of the mean adjusted using the method of [95] for within-participants comparison.

Fig 4 visualizes descriptive statistics of responses to each of 30 scales in both experiments. Scales are coded by the Big Five trait they reflect on: N(euroticism), E(xtraversion), A(greeableness), O(penness), and C(onscientiousness), and the number of the question in the NCS (Terracciano, 2005). We coded responses in the 1 to 5 interval such that a greater value reflects a higher (more intensive) level of a personality trait.

The personality profiles of the hypothetical speaker using Canadian and the hypothetical speaker using American words differ (Fig 4 and Table 3). T-tests in each facet (Bonferroni corrected for 30 comparisons) reveal significant differences in all 6 facets of Neuroticism (all ps < 0.001), judged as lower in the speaker using Canadian words. Agreeableness (higher in the speaker using Canadian words) is significantly different in all 6 facets (all ps < 0.01). Conscientiousness (higher in the speaker using Canadian words) is significantly all 6 facets (6 of 6 ps < 0.01). Facets E2 and E5, gregariousness and excitement seeking, are not significantly different, but Americans score significantly more highly in assertiveness (E3) and activity (E4), and score lower on interpersonal warmth (E1) and positive emotions (E6). Only 3 facets of openness, aesthetics (O2), feelings (O3) and ideas (O5) differ significantly, with Canadians scoring higher on all three. We remind the reader that the observed differences originated solely from a small sample of linguistic preferences: relatively narrow error bars further indicate a relative consensus in the language-based evaluation of personality traits. Interclass correlations [ICC(1,k)] at the facet level are very high, .98 for the hypothetical American speaker and.99 for the Canadian speaker, indicating agreement on average trait levels between raters drawn from each country. Crucially, however, these differences appear in line with prior descriptions of North American national stereotypes (see Introduction). Below, we confirm these differences resemble the national character stereotypes quantitatively, and show they are dissimilar from the personality profiles collected in large samples of Canadians and Americans using the NEO-PI-R instrument. [14]

**Table 3:** Facet level scores of hypothetical speakers using Canadian and American words.

		Canadian Use			American Use		
Facet		N	Mean	SD	N	Mean	SD
Anxiety	N1	191	1.87	0.92	195	3.10	1.24
Angry Hostility	N2	187	1.89	1.20	194	3.59	1.25
Depression	N3	188	1.86	1.22	195	2.98	1.20
Self-Conciousness	N4	189	1.87	1.02	193	2.79	1.18
Impulsiveness	N5	188	2.84	1.13	195	4.04	1.10
Vulnerability	N6	188	2.28	1.06	193	3.12	1.16
Warmth	E1	188	4.14	0.95	195	3.02	0.91
Gregariousness	E2	188	4.02	0.90	194	3.83	1.18
Assertiveness	E3	188	3.25	1.02	193	3.64	1.16
Activity	E4	189	4.12	0.81	194	3.46	1.02
Excitement-Seeking	E5	189	3.83	0.94	194	3.86	1.07
Positive Emotions	E6	189	4.20	0.81	193	3.19	0.97
Fantasy	O1	188	3.09	1.02	194	3.12	1.10
Aesthetics	O2	188	3.22	0.97	195	2.30	1.04
Feelings	O3	189	3.83	0.82	194	2.71	0.99
Actions	O4	189	3.24	0.97	193	3.14	1.07
Ideas	O5	186	3.60	0.88	195	2.65	1.05
Values	O6	187	3.51	0.88	194	3.52	1.05
Trust	A1	188	3.61	0.81	194	2.70	1.10
Straightforwardness	A2	188	3.84	0.82	195	2.87	1.13
Altruism	A3	189	3.90	0.83	194	2.43	0.91
Compliance	A4	188	3.27	1.06	194	2.20	0.97
Modesty	A5	187	3.59	1.01	195	2.17	0.90
Tender-Mindedness	A6	189	3.85	0.81	193	2.50	0.84
Competence	C1	188	3.88	0.74	193	2.34	1.00
Order	C2	188	3.53	0.85	194	2.16	0.91
Dutifulness	C3	188	3.66	0.87	194	2.28	0.87
Achievement Striving	C4	189	3.66	0.77	195	2.28	0.89
Self-Discipline	C5	189	3.65	0.81	195	2.56	1.01
Deliberation	C6	188	2.98	1.07	193	2.15	1.03

Mean facet level scores and standard deviation of hypothetical speakers who use very Canadian or very American words.

Our free-form question about the nation that the hypothetical speaker might belong to yielded the following results in Exp-US: out of 73 responses with the name of a nation, 67 (91%) were “American”. In Exp-Canada, 70 responses were given: 27 (39%) chose “American”, 29 Canadian (41%) and the remainder chose other nations. This suggests that typical American word choices strongly point to the USA as the origin, while the national identification is more ambiguous when typical Canadian choices are given.

We proceeded to a critical test of our claim that language productions typical of a nation mirror national stereotypes regarding that nation’s character. If the relationship holds, then we expect a strong correlation between the personality traits evaluated on the basis of language samples (“what is this speaker like”, Tables 1 and 2) and those traits evaluated based on one’s belief about a nation (“what is a typical American like” [14]). We extracted mean ratings associated with 30 personality scales in the NCS, as reported in [14]. Since intra-national evaluations were similar in the NCS data, we used responses made in California and Winnipeg test sites, respectively. For each of 30 scales, we calculated the difference between the US and Canadian rating: these represent differences in national stereotypes. Similar difference scores were obtained for each of 30 scales in our survey data: these scores reflect differences in personalities of hypothetical speakers who produce the most American and the most Canadian words and expressions.

**Fig 5:**
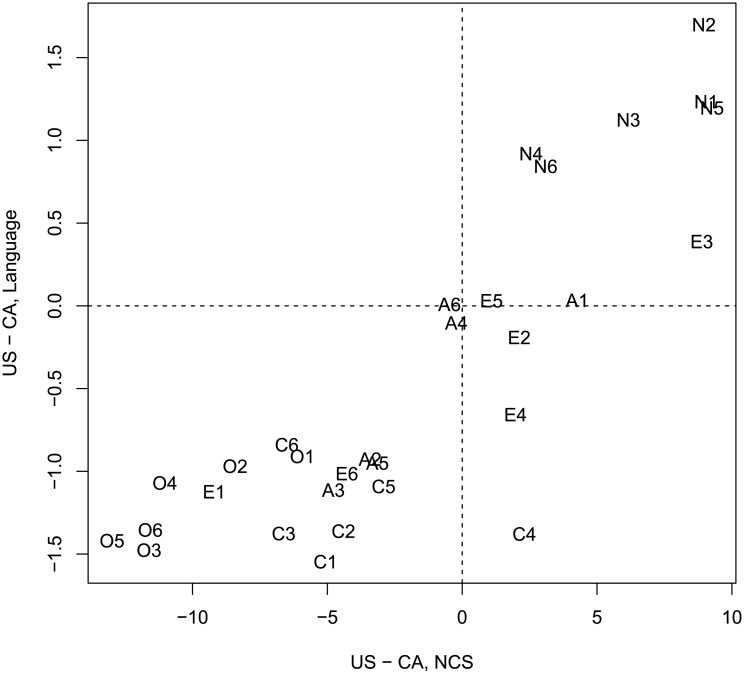
Scatterplot of difference scores: Language versus NCS. Scatterplot of difference scores in 30 personality traits evaluated based on language samples vs national character stereotypes according to the NCS. Codes represent question numbers in [14].

We observed a very strong convergence between two methods of evaluating cross-national differences in personality. Pearson’s correlation between two sets of difference scores was strong and highly reliable: r = 0.85, t(28) = 8.5, p < 0.001. Fig 5 further demonstrated that a vast majority of facets fell into the bottom left and top right quadrants of the two-dimensional space formed by two sets of difference scores: this confirms a high level of convergence between two independent estimations of national character. One of the biggest discrepancies was in estimates of select aspects of Extraversion, and Conscientiousness (E2 Gregariousness, E4 Activity, and C4 Achievement Striving) where raters of language productions judged Canadians to be higher in these facets than raters of the national character stereotype did.

**Fig 6:**
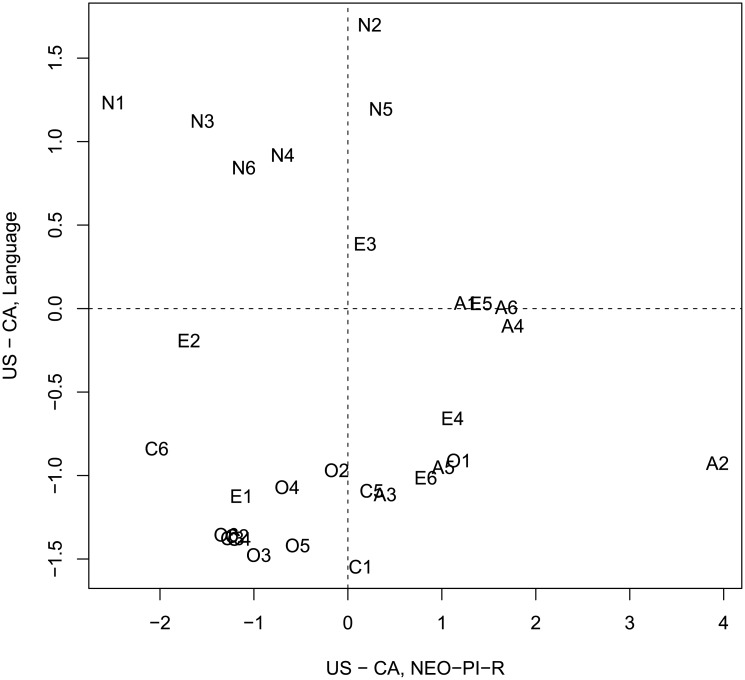
Scatterplot of difference scores: Language versus self-report NEO-PI-R. Scatterplot of difference scores in 30 personality traits evaluated based on language samples vs self-report NEO-PI-R. Codes represent question numbers in [14].

Importantly, language-derived difference scores yielded no reliable relation to compared to difference scores of personality traits from NEO-PI-R self-reports (see Fig 6),: r = -.04, t(28) = -0.25, p = 0.8. Our language based assessment is similar to the NCS, but dissimilar from the aggregated NEO-PI-R self-reports.

The overall tendency was quite clear-cut. Different groups of raters exposed to separate sets of words diagnostic of the US and Canadian linguistic patterns formed fairly consistent opinions of hypothetical individuals producing these patterns. These opinions were reliably different in most of the personality traits under evaluation. Moreover, the resulting differences between character assessments were very similar to the differences that emerged under another method of probing national stereotypes, i.e. direct evaluation of the national character using the NCS. This finding is remarkable given the small number of words and phrases (120 per nation) that our raters were exposed to. A long-standing argument in the literature claims that stereotypes about the national character cannot arise from the actual measurable (via self-reports) psychological differences in personality traits across nations. The present data points to another possible origin of stereotypes. It supports our hypothesis that the stereotypes mirror differences in the linguistic productions of the nations, and readers’ sensitivity to these differences.

Both Canada and the USA are demographically, geographically, socially and linguistically diverse, and this diversity was argued to also emerge in stereotypes about personality traits and optimism typical of geographic areas within each country and across countries (see [35, 51, 63] as well as the proposal of Canada and US represented by four distinct nations [80]). To ensure that findings reported above are not artifacts of our choice of entire countries as geographic domains, we replicated our analyses choosing two areas straddling the national border: the West Coast and the Great Lakes region. While not required by our method of linguistic analysis, in this instance we opted for geographically contingent areas. This choice increases the likelihood that neighboring nations have high familiarity with each other’s prevalent psychological traits, thus removing familiarity as a potential modulating factor [15]. Also, this choice constrains variability in climate and temperature, which have been argued to affect both personality traits and national character stereotypes [58, 96–98] but see [78]. Geographically adjacent areas are also more likely to share access to same environmental and natural resources. Finally, as argued by [78], geographically proximate nations tend to be more similar in their personality profiles, due to shared genes, history, culture or migration. Thus, whatever beliefs are formed on the basis of verbal behavior, they would be less likely grounded in the cross-border variability in personality traits [14]. The results in small regions are highly similar to the national trends, and are reported in the supplementary materials S2 and S3 Files. This convergence of results confirms the regional stability of linguistic patterns, even in the regions markedly different in their affluence, demographic and climatic parameters.

## 3 Study 2B: Human ratings of diagnostic words and emojis

The results of Study 2A are not without caveats: the stimuli didn’t contain emojis, we used no randomization of word order, and there is a noteworthy preference for racialized words in the American words. To address these concerns, we make some changes to the stimuli from Study 2A, and attempt to replicate it in a new sample. A difference between the American and Canadian word lists is the presence of racialized words such as *af, yo, bitches, finna, imma* in the American word list. These words may well be signal for the American national character stereotypes. Their presence could reflect the true demographic difference that a higher percentage of the American population is black. Alternatively, use of features of Black American English could be a component of how Americans construct their national identity via language. Regardless of why these words are among the most diagnostic of Americans, our results in Study 2A could be driven by the perception that the speaker using Canadian words is white, and the American words is black. To address this concern, we removed strongly racialized words *af, imma, bitches, yo, finna, bae, hella, lil, hoes, hoe* from the American stimulus list according to our intuitions and the etymology of the most American words where known.

A second concern is the absence of emojis in the stimuli for Study 2A, when Americans had a clear preference for using emojis. Our stimuli for the replication included the dominant emojis for each nation. We excluded one emoji from the Canadian word/emoji list: the Canadian flag emoji, which we considered too strong a cue for the national identity of the hypothetical speaker.

A third concern is that in Study 2A we explicitly stated that the words at the top of each nations list were the most diagnostic of the speaker’s personality, and performed no randomization of the words in each list. This may have introduced a primacy effect. To address this concern, we used the 120 top words and emojis for each nation, but randomized their order, and did not instruct participants that words at the top of the list were the most diagnostic. As Amazon Mechanical Turk does not allow for on-the-fly randomization, we produced 5 randomly ordered stimulus lists for each nation.

### 3.1 Method

The following study was approved by McMaster Research Ethics Board, protocol number #2011-165, titled “Research program of the eye-tracking lab at the Department of Linguistics and Languages.”

#### 3.1.1 Participants

Two experiments were conducted using the online crowdsourcing Amazon Mechanical Turk platform (mturk.com), one presenting participants with characteristic US words and another with characteristic Canadian words, henceforth Exp-Rep-Canada and Exp-Rep-US. 200 participants with the IP addresses based in the USA or Canada were recruited for each experiment: mean age in Exp-Rep-US was 36 years old (SD = 11.03; ages of 2 participants were unreported; 91 females, 2 gender other) and in Exp-Rep-Canada it was 35 (SD = 11.4; 1 ages unreported; 92 females, 4 gender not reported). Participants only took part in one of the experiments and each received a monetary compensation of 1 USD.

#### 3.1.2 Materials

**Fig 7:**
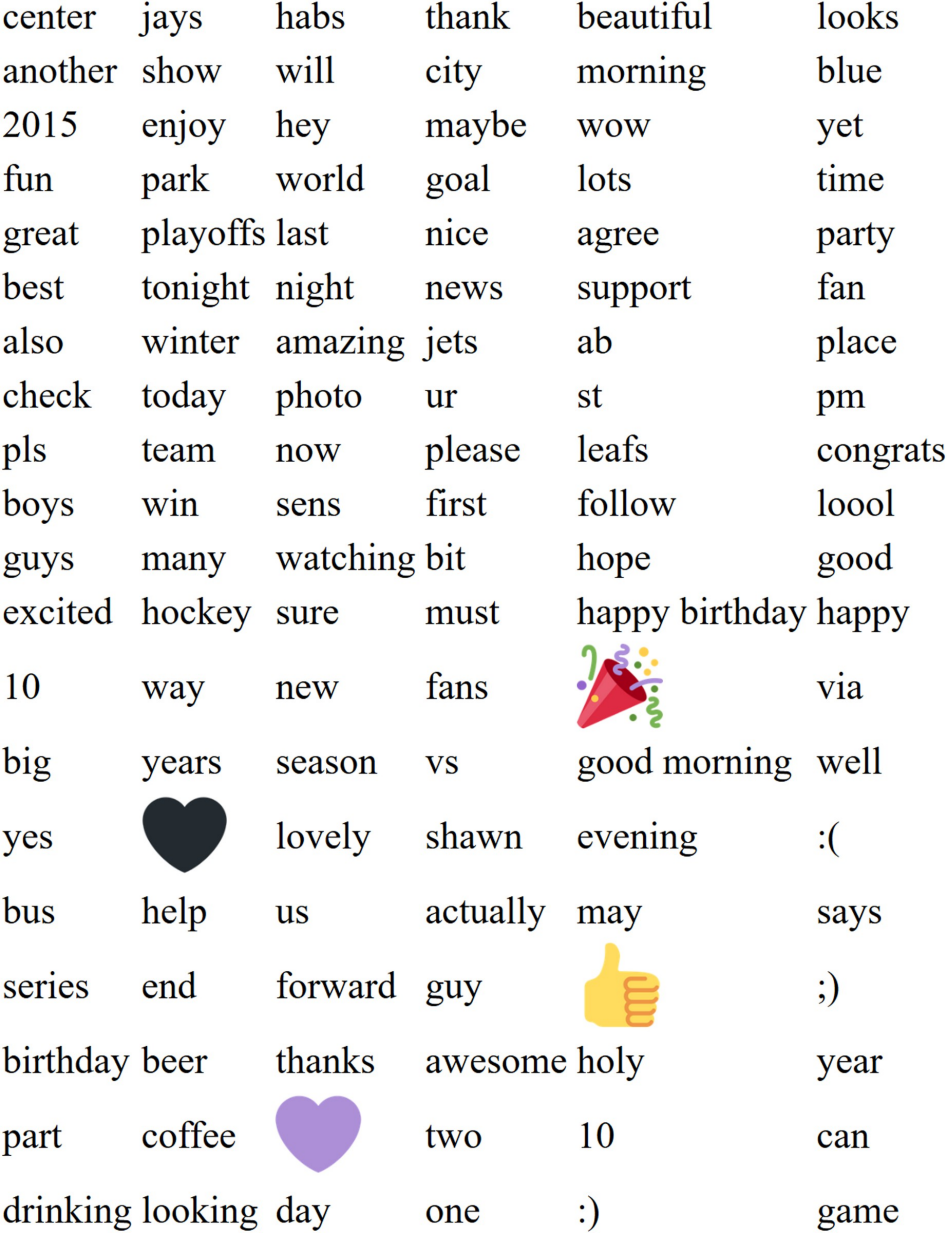
120 words and emojis most characteristic of Canadian tweets. 120 words and emojis most characteristic of Canadians used as stimuli in Study 2B.

**Fig 8:**
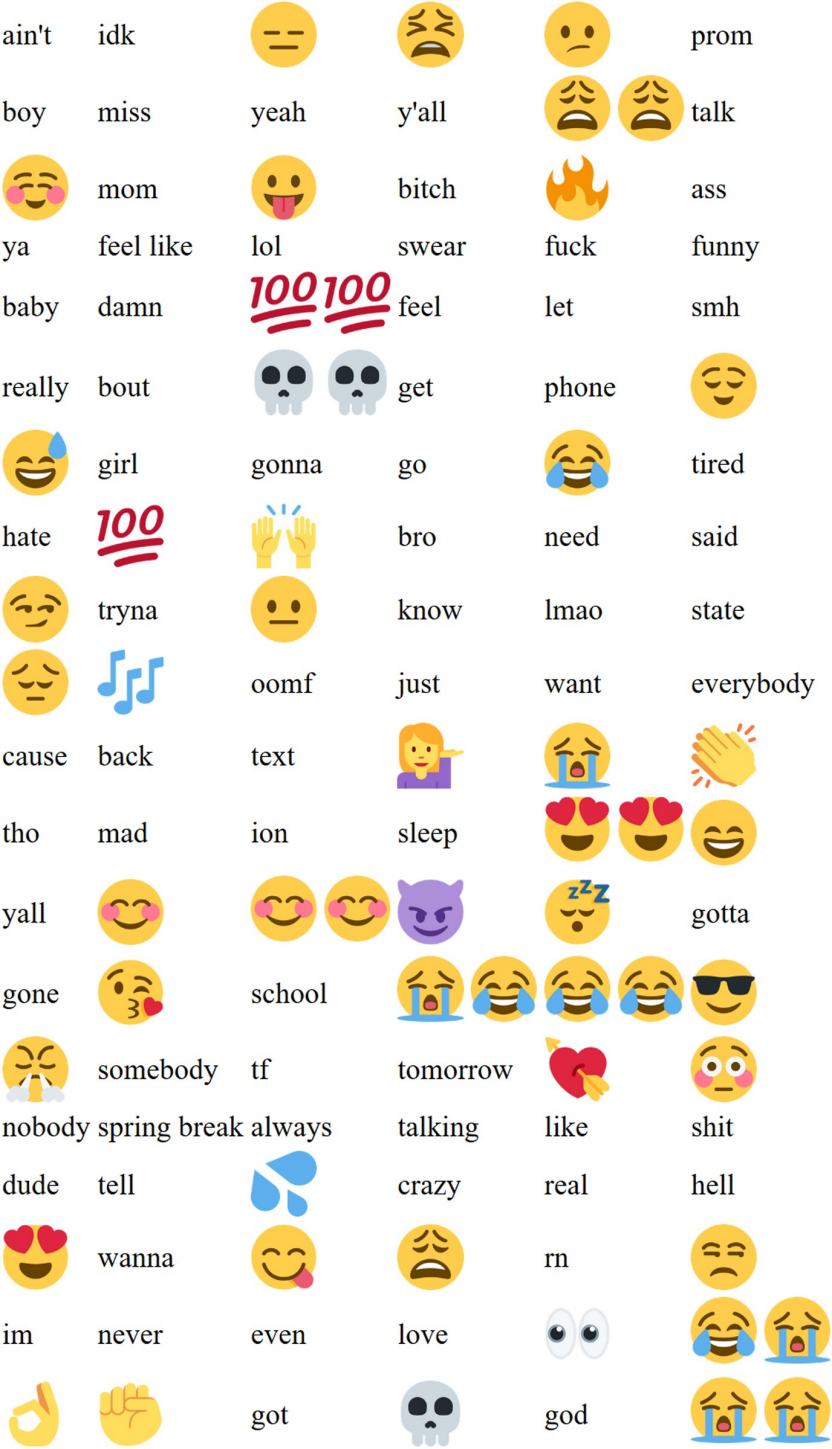
120 words and emojis most characteristic of American tweets. 120 words and emojis most characteristic of Americans used as stimuli in Study 2B.

We identified words and emojis with the most negative (Canadian) and most positive (US) z-scores, as estimated by the LORIDP measure. We removed strongly racialized words from the American list, and the Canadian flag emoji from the Canadian list. Then we selected 120 words and emojis from either end of the distribution (see Fig 7 for Canadian words and Fig 8 for American ones) with the greatest absolute z-scores and presented the two lists separately for evaluation in Exp-Rep-US and Exp-Rep-Canada, respectively. We produced 5 randomly ordered lists of these words for each nation. The evaluation of the hypothetical speakers personality was otherwise identical to Study 2A.

### 3.2 Procedure

The procedure was identical to Study 2A, save for the removal of the sentence “The top rows in the list are especially reflective of that person’s language, while rows in the bottom are somewhat less reflective.” from the instructions.

### 3.3 Results and discussion

Participants that filled out multiple lists had only their first response included in the data. 5 reponses in Exp-Rep-Canada and 2 responses in Exp-Rep-US took less than 60 seconds, and we excluded from consideration. This resulted in 174 valid responses for Exp-Rep-Canada and 184 valid responses for Exp-Rep-US. 200 ratings were excluded due to non-response. The resulting pool consisted of 5,134 data points for Exp-Rep-Canada and 5,406 for Exp-Rep-US.

**Fig 9:**
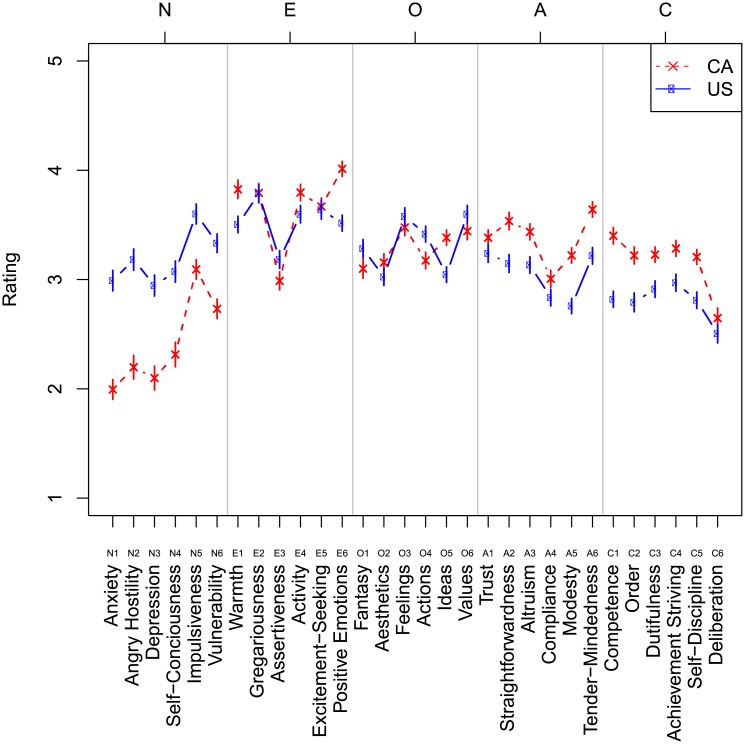
Mean ratings for 30 scales of the modified National Character Survey. Mean ratings for 30 scales of the modified National Character Survey, based on word and emoji characteristics of the US (blue) and Canada (red). Error bars stand for 1 standard error of the mean adjusted using the method of [95] for within-participants comparison.

Fig 9 visualizes descriptive statistics of responses to each of 30 scales in both experiments. Scales are coded by the Big Five trait they reflect on: N(euroticism), E(xtraversion), A(greeableness), O(penness), and C(onscientiousness), and the number of the question in the NCS (Terracciano, 2005). We coded responses in the 1 to 5 interval such that a greater value reflects a higher (more intensive) level of a personality trait.

Similar to Study 2A, the personality of the hypothetical users of Canadian and American words and emojis differ. The user of Canadian words and emojis is reliably less neurotic along neuroticism facets anxiety, angry hostility, depression, self-consciousness, and warmth, (all ps < 0.01). The user of Canadian words and emojis is reliably less extroverted on facet gregariousness, but more extroverted on facet positive emotion (ps < 0.05). The user of Canadian words and emojis has reliably higher openess on facet ideas (p < 0.01). The user of Canadian words and emojis is reliably higher in agreeableness on facets straightforwardness, altruism, modesty and tender-mindedness (ps < 0.01). The user of Canadian words and emojis is reliably higher in conscienciousness on facets competence, order, dutifulness, achievement striving, and self–discipline (all ps < 0.01). The difference scores in each facet from Study 2A and Study 2B are highly positively correlated (r = 0.95, t(28) = 15.67, p < 0.001), indicating agreement on the differences between speakers from each study.

However, the magnitude of the differences between the hypothetical speakers is reduced. Table 4 gives the differences between American and Canadian scores on each facet in each study. The mean absolute difference on all facets in Study 2A is 0.94 (sd = 0.48) on the 1-5 scale, but the mean absolute difference on all facets is only 0.4 (sd = 0.24) in Study 2B. Randomizing the order, removing racialized words, and including emojis seems to cut the magnitude of the differences between the speakers roughly in half.

**Table 4:** Differences between hypothetical speakers in Study 2A and Study 2B (Replication).

Facet	Study 2B	Study 2A
N1	0.86	1.23
N2	0.85	1.70
N3	0.71	1.12
N4	0.62	0.92
N5	0.37	1.20
N6	0.47	0.84
E1	-0.46	-1.12
E2	-0.15	-0.19
E3	0.06	0.39
E4	-0.34	-0.66
E5	-0.18	0.03
E6	-0.63	-1.01
O1	0.05	0.03
O2	-0.27	-0.93
O3	-0.04	-1.11
O4	0.10	-0.10
O5	-0.48	-0.95
O6	0.02	0.01
A1	-0.28	-0.91
A2	-0.53	-0.97
A3	-0.44	-1.48
A4	-0.31	-1.07
A5	-0.60	-1.42
A6	-0.56	-1.35
C1	-0.72	-1.55
C2	-0.57	-1.36
C3	-0.45	-1.38
C4	-0.45	-1.38
C5	-0.53	-1.09
C6	-0.28	-0.84

Differences between ratings of the personality of the hypothetical users of Canadian and American words/emojis in Study 2A and Study 2B.

**Fig 10:**
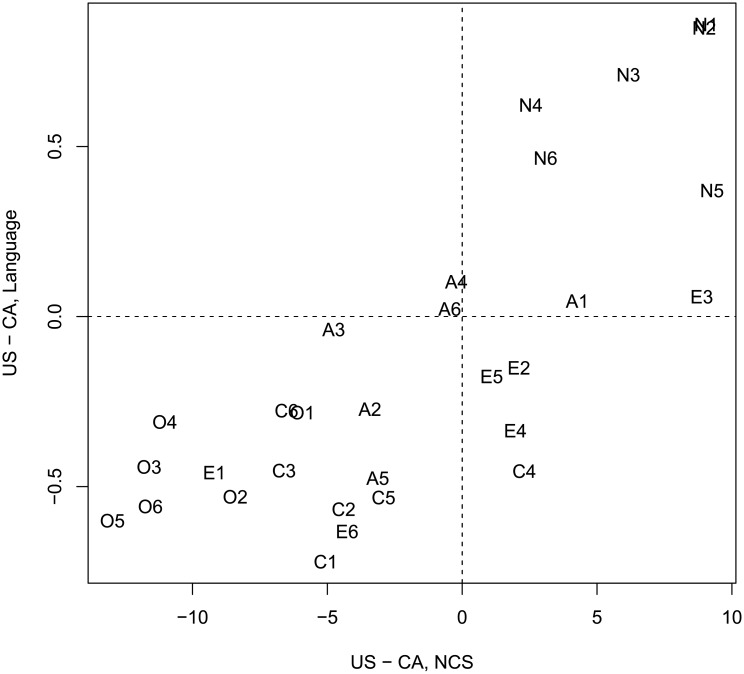
Scatterplot of difference scores: Language versus NCS for Study 2B. Scatterplot of difference scores in 30 personality traits evaluated based on language samples vs national character stereotypes according to the NCS. Codes represent question numbers in [14].

**Fig 11:**
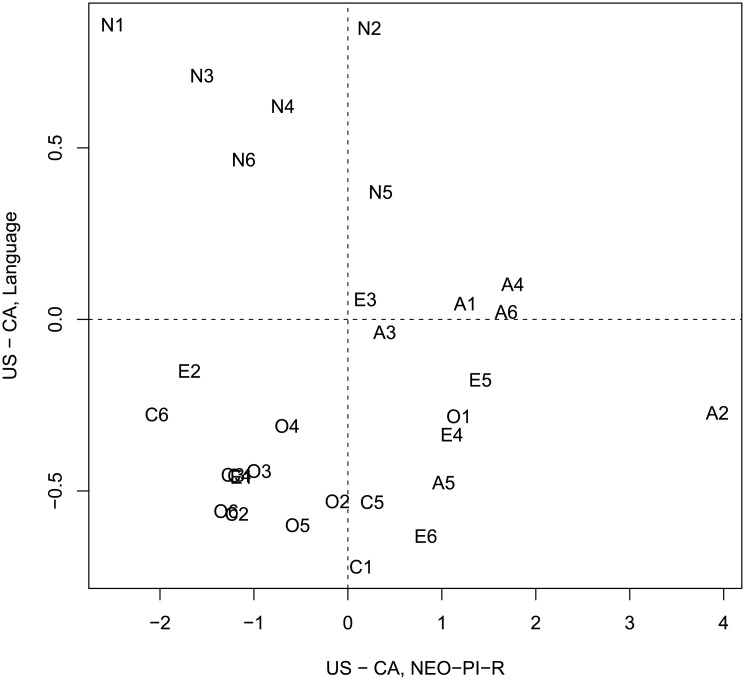
Scatterplot of difference scores: Language versus NEO-PI-R for Study 2B. Scatterplot of difference scores in 30 personality traits evaluated based on language samples vs NEO-PI-R. Codes represent question numbers in [14].

Similar to Study 2A, the differences between the hypothetical speakers are aligned with the results of the NCS, but not with the NEO-PI-R [14]. Fig 10 plots difference scores derived from words and emojis in Study 2B, with differences obtained from the NCS. Similarly to Study 2A, there is a strong and reliable relationship between the difference scores (r = 0.77, t(28) = 6.4, p < 0.001). However, no such relation is found (see Fig 11) when language-and-emoji derived difference scores are compared against results from the NEO-PI-R (r = -0.13, t(28) = -0.68, p > 0.05). Thus we largely replicate the results of Study 2A, albeit with a reduced magnitude of difference between the hypothetical speakers.

The results of our open-response question about the nationality of the speaker yielded 36 responses of American, 18 Canadian, 12 English, and 2 Japanese for the hypothetical speaker of Canadian words. For the American speaker, results yielded 49 responses of American, 9 English, 1 Chinese and 1 German. This is similar to Study 2A, where American words seemed to more strongly point to an American origin for the speaker than Canadian words point to Canada.

Thus we largely replicate the results of Study 2A even when racialized words are excluded from the American output, emojis are included, and the order of the words/emojis is randomized.

## 4 General discussion

National character stereotypes, i.e., beliefs about the personality profiles of members of nations, present a paradox. They are prolific and reliable, yet aggregated self-reported personality profiles of peoples composing nations bear no consistent relation to national character stereotypes. The most well studied example, Canadians and Americans, are believed to have divergent personalities by the peoples of both nations, yet self-report evidence fails to show any such divergence [13, 14]. This disagreement is particularly puzzling given that stereotypes regarding the nation’s positivity or optimism are generally aligned across different types of measurements (Study 1B).

We argue that disagreement between national character stereotypes and the self-reports of the personalities of the people composing nations may be valid, but national character stereotypes may also be informed, in part, by verbal behaviour. In a corpus of 40 million tweets, we quantified the over- and under- representation of the words and emojis in each nation’s collective linguistic output. Although Canadian and Americans use the vast majority of their shared English lexicon the same, some differences in word use emerge. Using two original experiments (Study 2A and 2B) with the most nationally diagnostic words and emojis as the stimulus, we showed that participants form systematically diverging personality judgments of speakers using the most diagnostic words of each nation. Personality profiles formed by these judgments closely match respective national character stereotypes (Fig 5), and do not match self-report measured personality traits of Canadians and Americans (Fig 6). As exemplified in the collective Twitter activity of each nation, Canadians’ and Americans’ most distinct linguistic behaviours are consistent with their stereotypes. Distinctively Canadian words are more positive (Fig 2). Speakers using the most nationally diagnostic language are perceived as different across all facets of neuroticism (Canadians are lower), agreeableness (Canadians are higher), and conscientiousness (Canadians are higher) and select facets of extraversion (Canadians are higher on interpersonal warmth and positive emotions, lower in assertiveness) and openness.

National character stereotypes mirror the distinctive language use of nations. Assuming that mean trait levels from aggregated self-reports are valid for intercultural comparison, our data suggests that national character stereotypes might reflect something true. Unexpectedly, that something isn’t people and their personality traits. However, national character stereotypes may be true of the differences in collective linguistic behaviour between nations. This statement begs a chicken-and-an-egg question: what is the origin of the distinct linguistic behavior? The present data only allow for a speculation on this topic and point to an important direction of future research. As stated in the Introduction, we speculate that language is a means by which a national identity can be constructed, in opposition to a generic or specific out-group. On an individual level, changing one’s language use to fit some context is probably familiar—not swearing in church, relaxing into an accent at home, or choosing formal language in an academic paper. What we show is that when the linguistic choices of the individuals that make up a nation are added up, they do not match aggregated self-reports of personality profiles of that population. Rather what emerges is consistent with the national character stereotype. In other words, thinking that individual Canadians are quiet and nice, or individual Americans are brash and outgoing because their combined behaviors point to those traits, might be an ecological fallacy.

It is a well established finding that people who use certain words (on Facebook) tend to vary in personality in systematic ways [51]. For age, gender, and political stereotypes on Twitter, people can accurately infer which features of language are correlated with ground truth (see Introduction and [62]). In other words, it seems that at an individual level, we have some accurate knowledge of what linguistic behaviour likely tells us about someone’s traits. Perhaps when national character stereotypes are invoked, they are driven by a reverse-ecological fallacy—to assume that our somewhat accurate knowledge of how individuals’ traits vary with their linguistic behavior must also generalize to the aggregate, national level. It can be true that people who say “best friend” more often tend to be more extraverted, and that Americans say “best friend” more often than Canadians, and yet this does not mean that Americans are more extraverted than Canadians. However, our evidence does not establish whether language is a cause of national character stereotypes, only that diagnostic language use as exemplified on Twitter leads to systematically different personality judgments of hypothetical speakers using that language, which is consistent with national character stereotypes.

It is important to note that we are exploring differences in word usage in each nation, where they exist, and the relation between these differences in word usage and personality traits. Canadians and Americans are speakers of the same language, and Canadian and American English are very similar dialects. Furthermore, most language use will involve words that have no particular association with personality traits (e.g. function words such as *the, a, of* etc.). And as far as we know, only a subset of the English lexicon has any particular association with personality traits, [51] identified around 8000 words with frequency of usage linked to personality traits, yet the English lexicon is composed of many hundreds of thousands of words, depending on how “word” is defined. There is no a priori reason why words with higher LORIDP scores in our data need to have any bias in their association with personality traits. Our argument is contingent on Canadians and Americans being sensitive to differences in language use, where those differences exist.

A logically possible alternative explanation of our findings is that self-report personality tests like NEO-PI-R suffer from a systematic bias, which blurs a faithful correspondence between national character stereotypes and true aggregated personality traits. One proposed candidate for such a bias [31, 32] is the reference group effect (RGE), or the tendency to base responses “not on respondents’ absolute level of a construct but rather on their level relative to a salient comparison group” [99]. [59] show that the RGE has little if any influence of language based assessment of personality. Also, the RGE is not an undesired bias but rather a core feature of the NCS survey, where reference groups are given explicitly.

We note that, if present, the RGE does not influence the relationship that we observed between language-based personality profiles (Study 2A and 2B) and independent results of the NCS survey [14]. It might however mask existing relationships between stereotypical and actual Canadians and Americans. Consider the trait of Neuroticism and the conditions under which the RGE would obscure the results of a test like NEO-PI-R. Stereotypical Americans are slightly more than 1 SD higher on Neuroticism than stereotypical Canadians [14], yet their Neuroticism scores in NEO-PI-R are very similar to those of Canadians (within 0.2 SD from the mean of standardized T-scores). If this discrepancy is due to the RGE and the stereotypes reflect the ground truth, an average American downplays their neuroticism when responding to NEO-PI-R. This, in turn, is only expected if the reference group for that average American is more neurotic than that person. So, under the RGE predictions, an average American is both less neurotic than their reference group and also Americans on average are highly neurotic. As pointed out by [13], both statements cannot be true at the same time. We conclude that a more parsimonious explanation for the discrepancy at stake is that national character stereotypes are inaccurate.

A weaker interpretation of the RGE is that it introduces noise to self-reports, rather than shifts in specific directions. Indeed, differences exist between NEO-PI-R scores across nations, and the RGE might attenuate them. Differences between NEO-IR-R scores drawn from Terraciano et al 2005 are given in Table 5. In this case, we expect to observe that fluctuations in the NEO-PI-R scores are minor but still match their respective national character stereotypes. Fig 6 rules out this possibility: there is no reliable correlation between difference scores based on NEO-PI-R and our language-based version of the NCS.

**Table 5:** NEO-PI-R difference scores and language-based difference scores.

	Facet					
NEO-PI-R	1	2	3	4	5	6
Neuroticism Difference	-0.25	0.02	-0.16	-0.07	0.03	-0.11
Extraversion Difference	-0.11	-0.17	0.02	0.11	0.14	0.08
Agreeableness Difference	0.12	0.39	0.04	0.18	0.10	0.17
Openness Difference	0.12	-0.01	-0.09	-0.06	-0.05	-0.13
Conscientiousness Difference	0.01	-0.12	-0.12	-0.12	0.03	-0.20
Language	1	2	3	4	5	6
Neuroticism Difference	1.01	1.39	0.92	0.75	0.97	0.69
Extraversion Difference	-0.92	-0.16	0.32	-0.54	0.02	-0.83
Agreeableness Difference	-0.74	-0.79	-1.20	-0.87	-1.16	-1.10
Openness Difference	0.03	-0.75	-0.91	-0.08	-0.78	0.01
Conscientiousness Difference	-1.26	-1.11	-1.12	-1.12	-0.89	-0.68

NEO-PI-R Difference scores and Language-based Difference scores (US − Canada) between the US and Canada from [14] and our Study 2A. Scores from both studies are presented in units of standard deviation. Most differences on facets between Canadians and Americans are less than 2 fifths of a standard deviation for NEO-PI-R. Language based differences tend to be larger than NEO-PI-R differences.

We cannot definitively rule out the presence of the RGE, given the currently available data. Data supporting the presence of cultural differences in the standards of evaluation of personality traits between Canadians and Americans would be a positive step towards resolving this issue without invoking parsimony.

### 4.1 Limitations

Several criticisms of the exploratory study presented here are possible. First, Twitter is a relatively new platform, and yet the national character stereotypes of Canadians and Americans clearly existed long before and outside Twitter. What we observe on Twitter should be replicated in other corpora, including historical corpora, of Canadian and American English. We also cannot rule out definitively, with the data we have, if the RGE explains the discrepancy between NEO-PI-R self reports and our language data and the NCS. Language is also one possible source among of national character stereotypes among many, such as visual media. As well, we must note that our data does not establish causation of national character stereotypes by differential language use. Study 2A and 2B does establish that exposing participants to the most diagnostic words of each nation causes them to form a systematically different personality profile of a speaker who uses these words. Whether this has ecological validity is questionable. Outside of this narrow experimental context it may be that differences in the national language use and national character stereotypes are both caused by some other underlying factor. It is also important to acknowledge that while Twitter provides a sample large in both number, demographics, and geographical coverage, it is still not a random sample of the population.

We verified that the presence of highly racialized words in the American output does not eliminate the difference in the perceptions of hypothetical speakers of American and Canadian words (Study 2B). It is however possible that these words contribute to the larger difference between the hypothetical speakers between Studies 2A and 2B. It could be the case that use of African American English, either by native speakers or appropriated by non-native speakers online, is one of the drivers of the American national character stereotype. Future work should explore the contribution of African American English features to the American national character stereotype.

### 4.2 Future directions

Comparisons of other national character stereotypes between different countries would give further valuable insight into national character stereotypes. Cross-cultural comparisons using the methods we present are limited, currently, to nations sharing a common language. Whether national character stereotypes are manifested in the distinctive linguistic behaviours of nations not sharing a language remains to be seen. At the level of words, such comparisons could be undertaken using the methods in this paper provided a set of translation pairs of words (i.e. “cat” and “chat” in English and French). This would allow application of the LORIDP statistic corpora from two different languages.

Our Study 1B assumes that Americans and Canadians agree on how positive they perceive words to be. Indeed, we also inherently ignore differences in perceptions of posivity of words between speakers as well. Future work could reduce these potential biases by developing country-specific lexica of positive words, and taking into account individual differences in the perception of how positivity words are. In a similar vein, it could be that the observed differences in positivity are driven by national differences in why Canadians and Americans use Twitter.

As the aggregated self-reports of individuals do not seem to explain national character stereotypes, [13] emphasizes the role of culture and other factors (climate, historical influences etc.) in maintaining national character stereotypes. Yet, cultural factors are also challenging to measure and compare. Social media activity discussing interactions between Canadian and American leadership fulfilling or subverting national character stereotypes provide a rich set of natural experiments in which national character stereotypes play a salient role, and often an explicit one. Recent and ongoing political events evoke stereotypes of Canadians and Americans. In 2013, Former Toronto Mayor Rob Ford became an international target for comedy because of his “un-Canadian” behaviour. Former President Bill Clinton remarked on late-night comedy show *Jimmy Kimmel Live!* “[Ford] has absolutely destroyed every stereotype people have about Canadians… you know, that Canadians are upbeat, optimistic, can-do, they’re embracing, they’re inclusive…” More recently, Federal elections in Canada and the US have produced leaders with strongly contrasting personas (arguably aligned with their respective national character stereotypes) and policy positions. These unfolding events and the social media activity around them present unique opportunities for studying national character stereotypes. An additional challenge for future research will be to explain why stereotypical personality traits are so different from the personality traits obtained from actual members of nations, but stereotypical levels of optimism align well with the levels of optimism that these people demonstrate.

Finally, our findings in the two nations and two cross-border regions shift the question from “where do stereotypes come from” to “where do distinctive linguistic behaviors come from”. A study of how national group identities are manifested and engendered by the means of language is a necessary continuation of the present work.

To conclude, our findings contribute to the literature by making a step towards resolving a paradoxical lack of psychological grounding for stable and robustly observed national character stereotypes. These stereotypes may be inaccurate with respect to the traits of individuals, but they are accurate with respect to the differences in collective linguistic behaviour of nations.

## Supporting information

S1 File Addtional analyses of nationally diagnostic language based on lexica of words associated with personality traits. (PDF)

S2 File Additioinal analyses within a western subregion of Canada and the US. (PDF)

S3 File Additional analysis within an eastern subregion of Canada and the US. (PDF)

S4 File Additional details about the LORIDP statistic. (PDF)

S5 File The most Canadian and American words. (ZIP)
