# Supplementary material for: National character stereotypes mirror language use: A study of Canadian and American tweets
Source: PLoS One. 2018 Nov 21;13(11):e0206188. doi: 10.1371/journal.pone.0206188 (PMC6248921; doi:10.1371/journal.pone.0206188)
Supplement: S1 File — (PDF) [file pone.0206188.s001.pdf]

S1

Another method of gauging the personality traits of national language is to compare it against existing lexica of words scored on their associations with personality traits. Estimates of how indicative words are of personality traits is available from [1]. This dataset is based on the myPersonality [2] sample of 75,000 Facebook users who completed the Five Factor Model International Personality Item Pool questionnaire [3] based on NEO-PI-R [4]; 700 million words of public Facebook posts have also been available from those same users. Using an open-vocabulary regression approach, [1] predicted scores on all personality traits from word usage in public Facebook posts. Regression coefficients taken from these models are then a measure of association between a word and a given personality trait (extraversion, agreeableness, conscientiousness, neuroticism, or openness). Armed with the Big-5 regression weights and LORIDP scores for a large set of words, we can construct a second assessment of if very Canadian or very American words are associated with certain personality traits, though not at the facet level.

Figures A to F present the words with extreme associations with positivity, negativity, and personality traits, within each country.

Figure G presents the associations with openness of words binned by ranked LORIDP. Across the LORIDP distribution, Canadian words have higher average associations with openness than American words, (Cohen's d = .24 to .61). Figure H presents the associations with conscientiousness of words binned by ranked LORIDP. Canadian words are associated with higher conscientiousness only among the most nationally distinctive words ($d = 0.53$, $p < 0.001$). Figure I presents the associations with extraversion of words. Canadian words are associated with lower extraversion robustly across the LORIDP distribution (d = -1.01 to -0.7, all ps < 0.001). This difference is extraversion is remarkably large, given that extraversion at the facet level shows some differences between nations in the NCS (e.g. Canadians are perceived as higher in warmth and positive emotions, but lower in assertiveness). Below, we perform another assessment of the personality traits associated with the national language, which captures these stereotypical differences between Canadians and Americans at the facet level. Figure J presents the associations with agreeableness of words. Canadian words are associated with higher agreeableness in the 4 most diagnostic LORIDP bins (d = 0.93 to 0.37). Figure K presents the associations with neuroticism of words, where there are no reliable differences among the most nationally diagnostic words.

Figure A: The most positive and negative words used by each nation, Canada (2 left columns) and the United States (2 right columns).

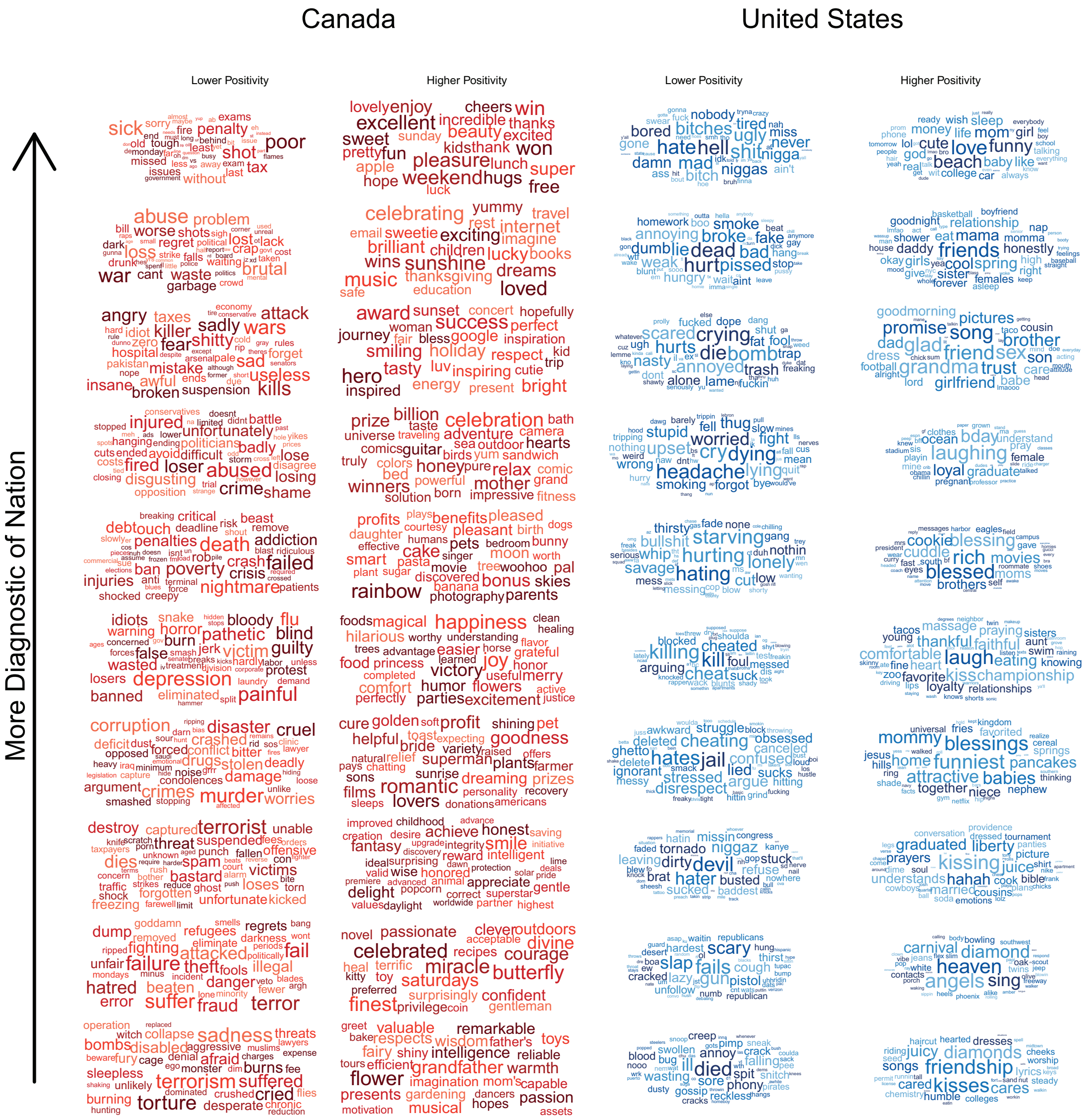

Size indicates relative negativity/positivity of each word. Words at the top of the figure are most diagnostic of each nation, and and less diagnostic towards the bottom.

Figure B: The words most and least associated with openness used by each nation, (2 left columns) and the United States (2 right columns)

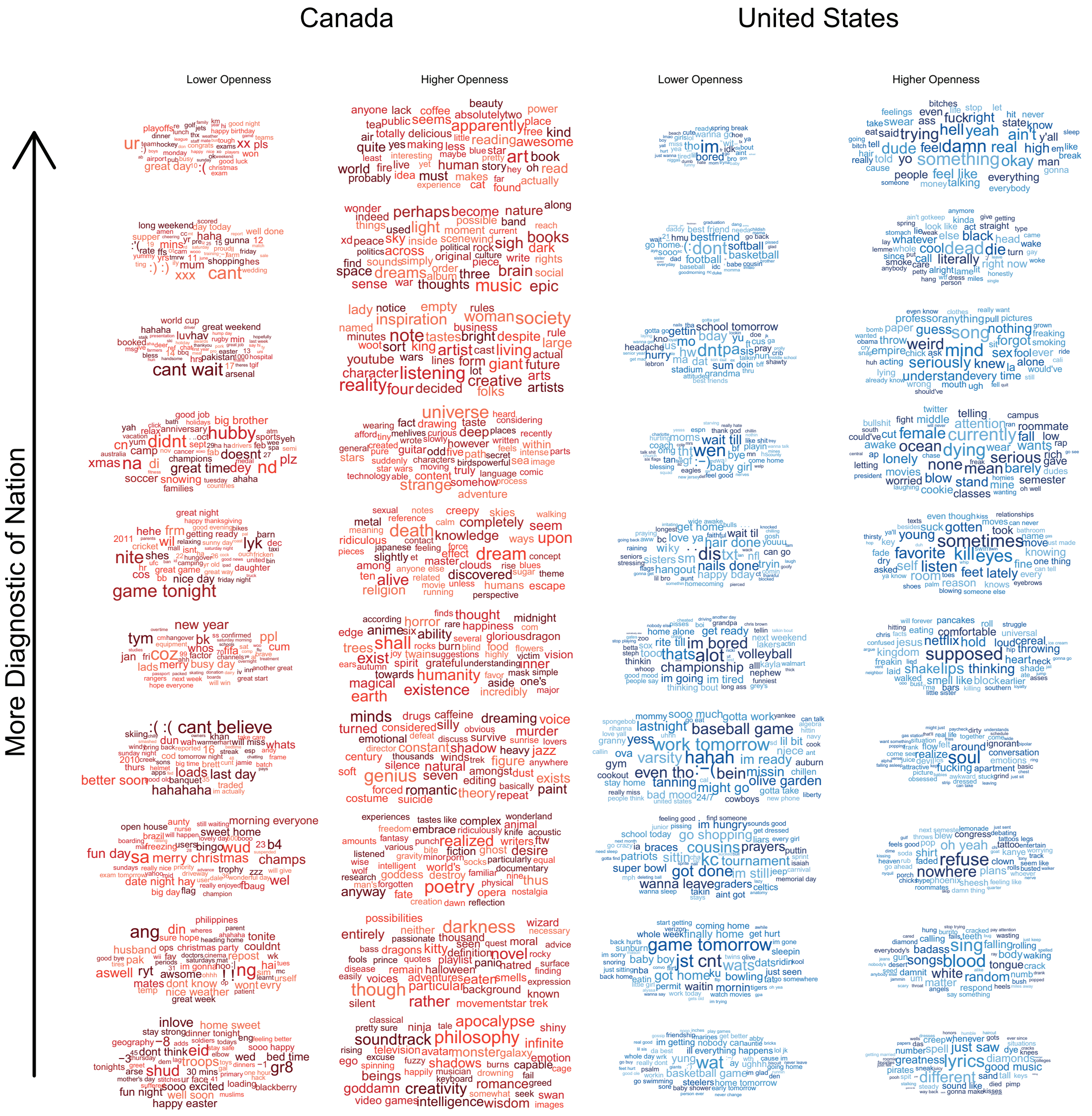

Size indicates relative openness of each word. Words at the top of the figure are most diagnostic of each nation, and and less diagnostic towards the bottom.

Figure C: The words most and least associated with conscientiousness used by each nation, (2 left columns) and the United States (2 right columns)

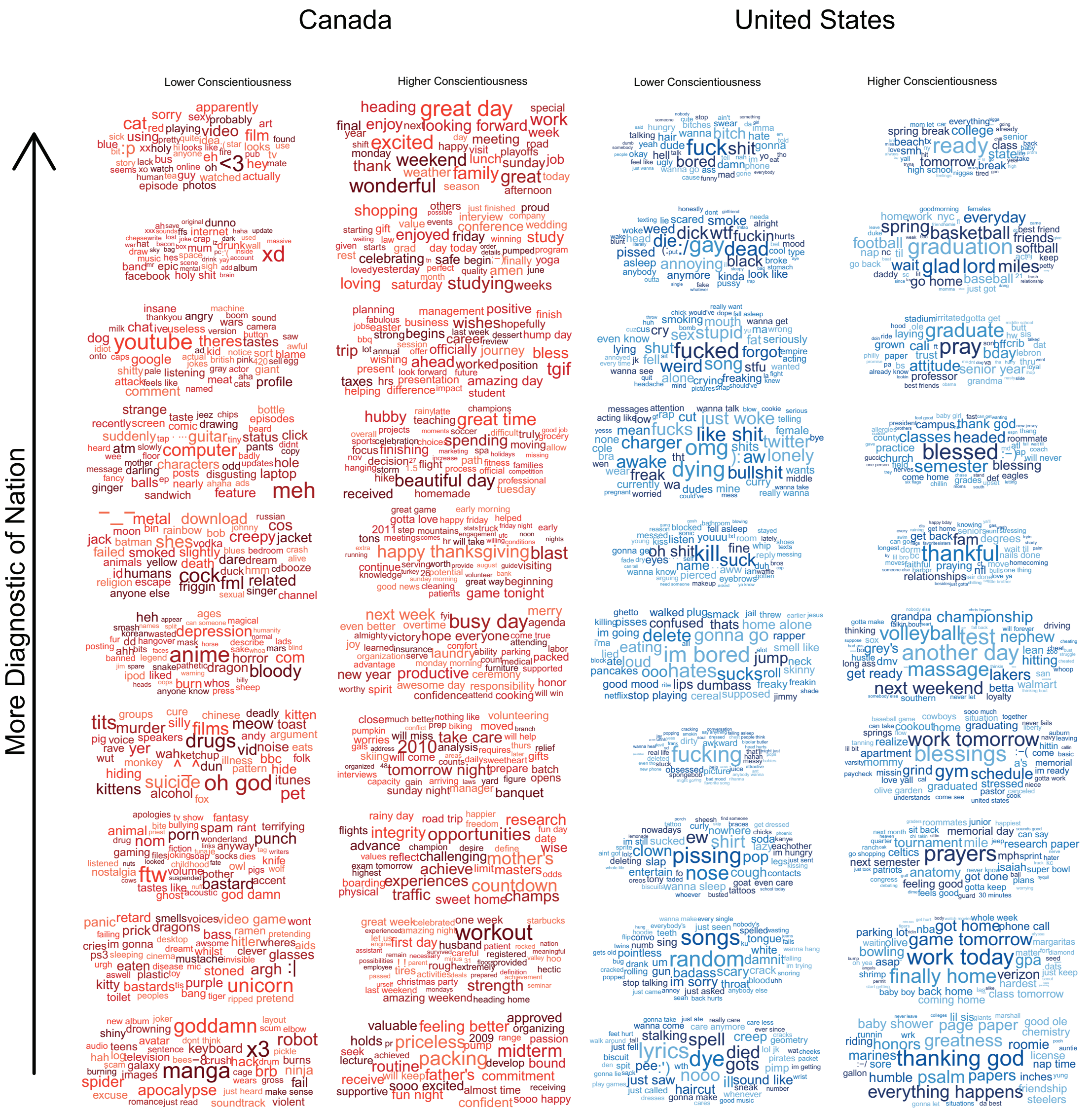

Figure D: The words most and least associated with extraversion used by each nation, (2 left columns) and the United States (2 right columns)

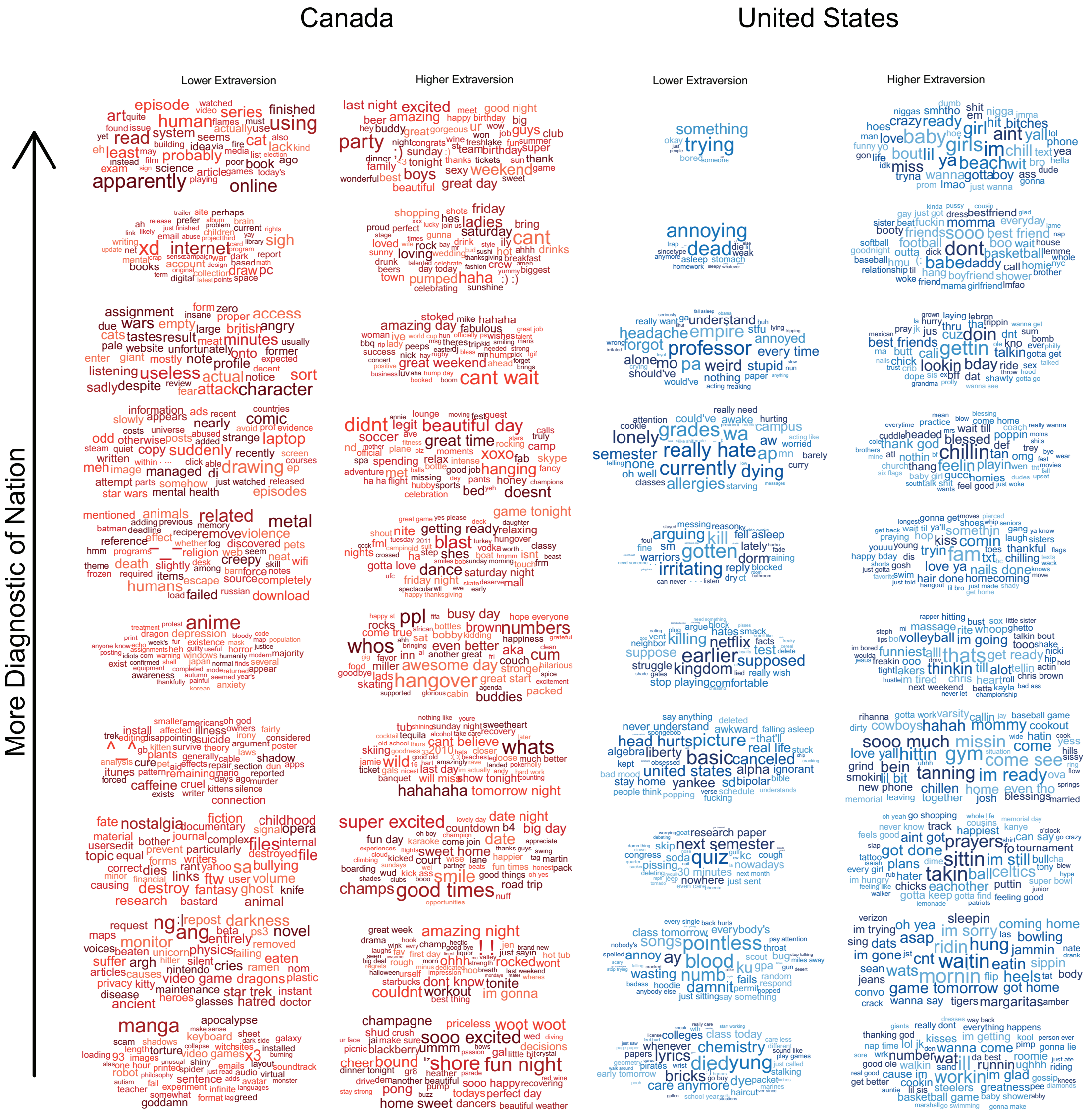

Size indicates relative extraversion of each word. Words at the top of the figure are most diagnostic of each nation, and and less diagnostic towards the bottom.

Figure E: The words most and least associated with agreeableness used by each nation, (2 left columns) and the United States (2 right columns)

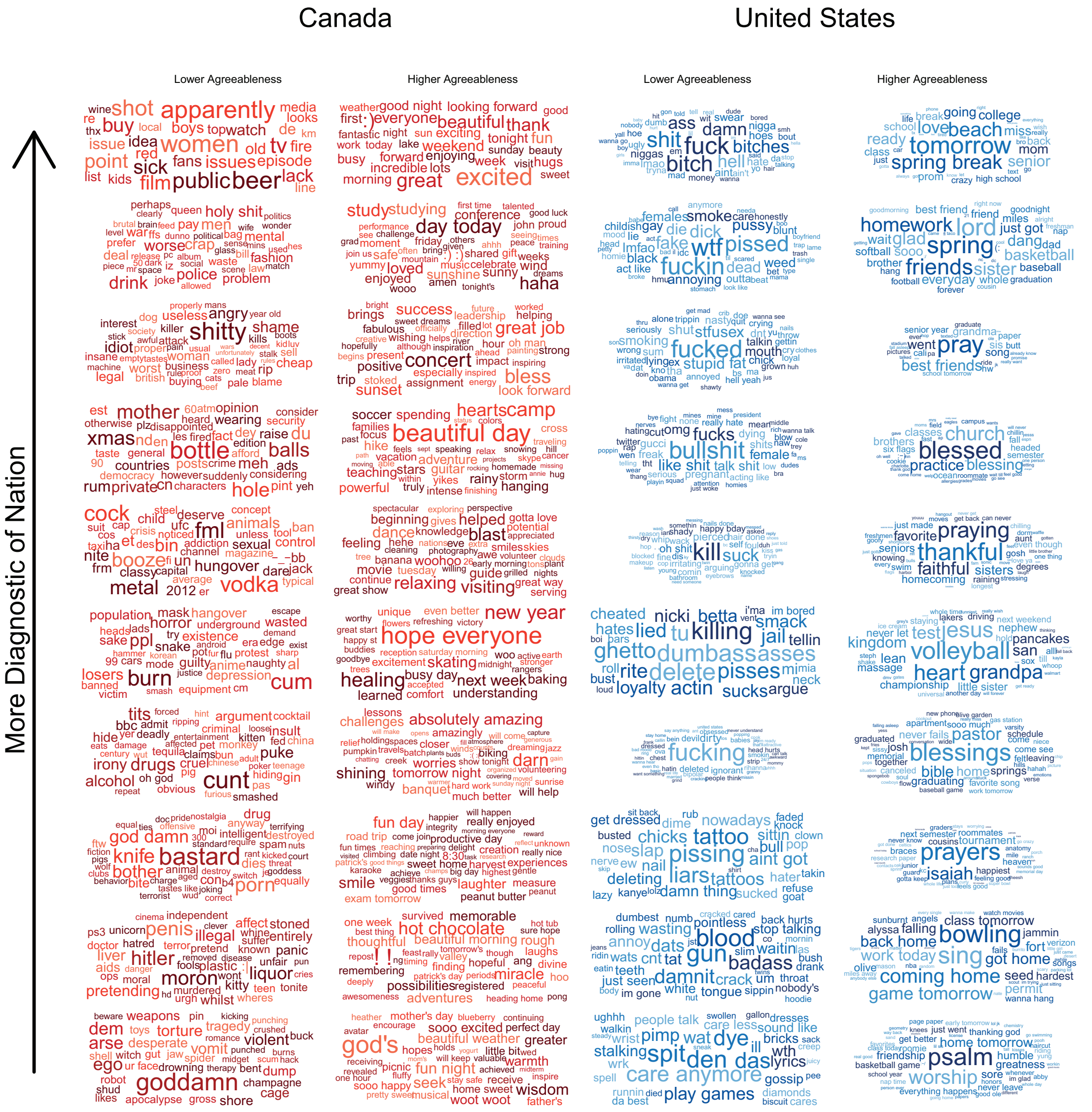

Size indicates relative agreeableness of each word. Words at the top of the figure are most diagnostic of each nation, and and less diagnostic towards the bottom.

Figure F: The words most and least associated with neuroticism used by each nation, Canada (2 left columns) and the United States (2 right columns)

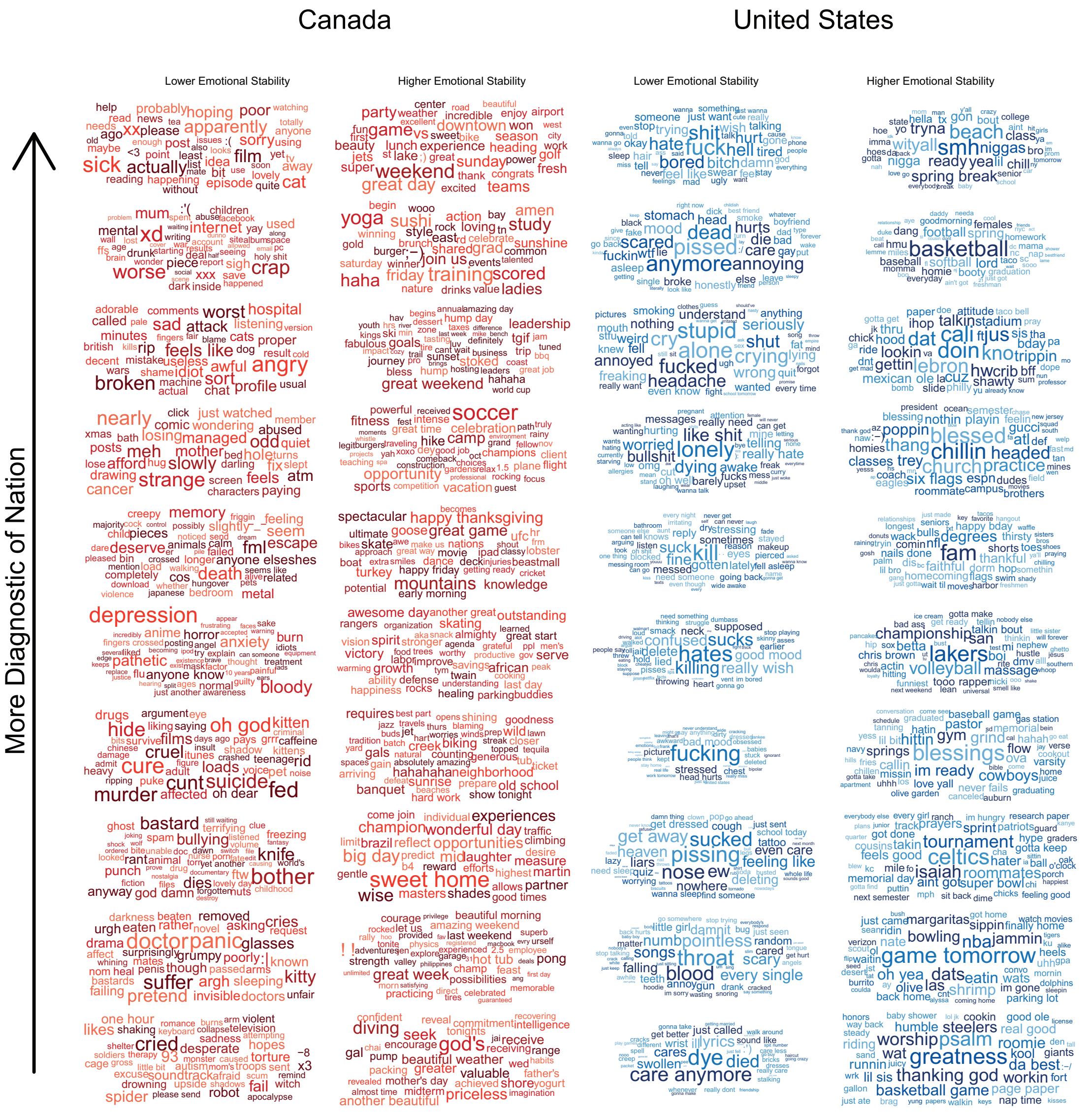

Figure G: Relative association with openness of very American (blue) versus very Canadian (red) words

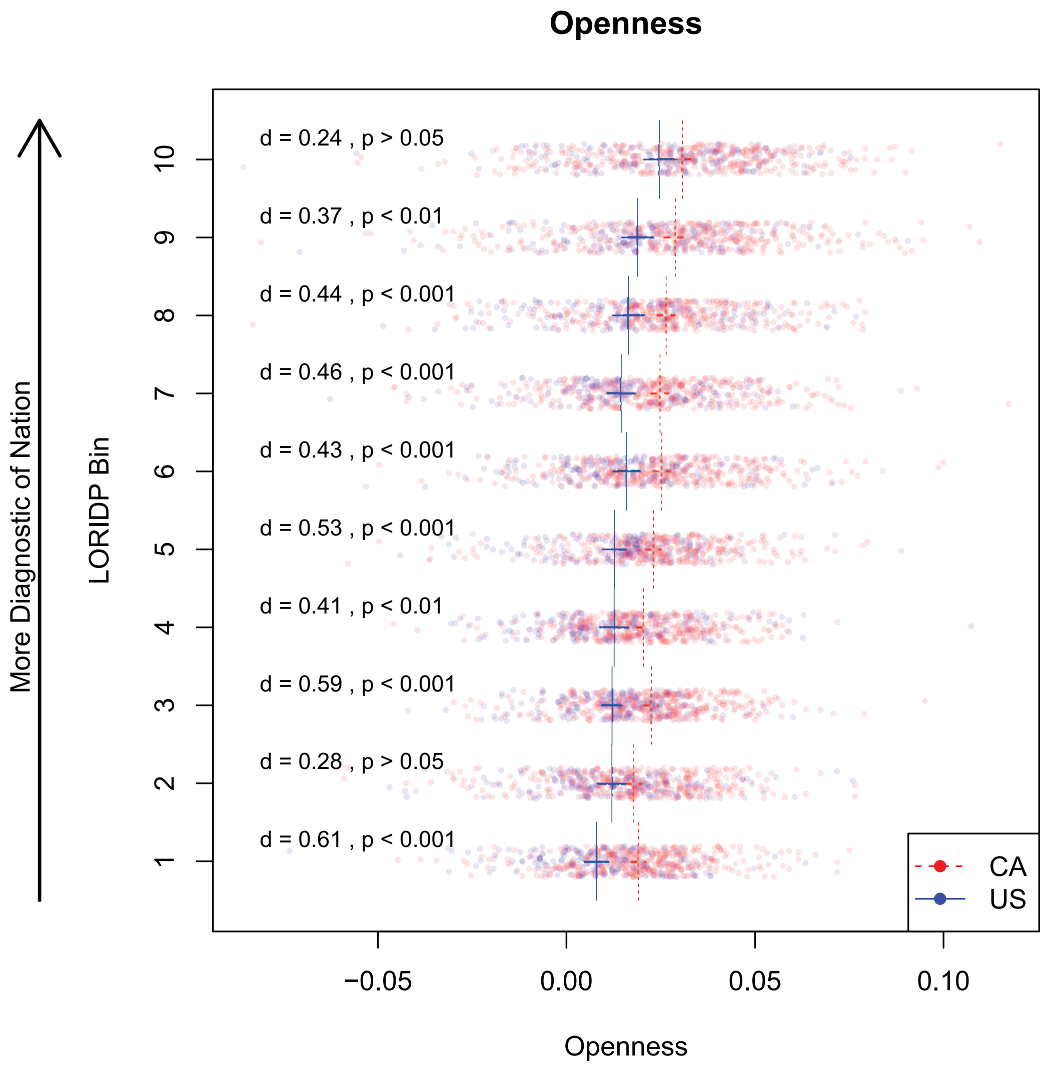

Verticle lines indicate mean openness of American and Canadian words in each bin. Hotizontal lines are 95% confidence intervals of the means. Cohen's d and p-values for t-tests within each bin are reported in the left of the figure. Canadian words are consistently associated with greater openness across the LORIDP distribution.

Figure H: Relative association with conscientiousness of very American (blue) versus very Canadian (red) words

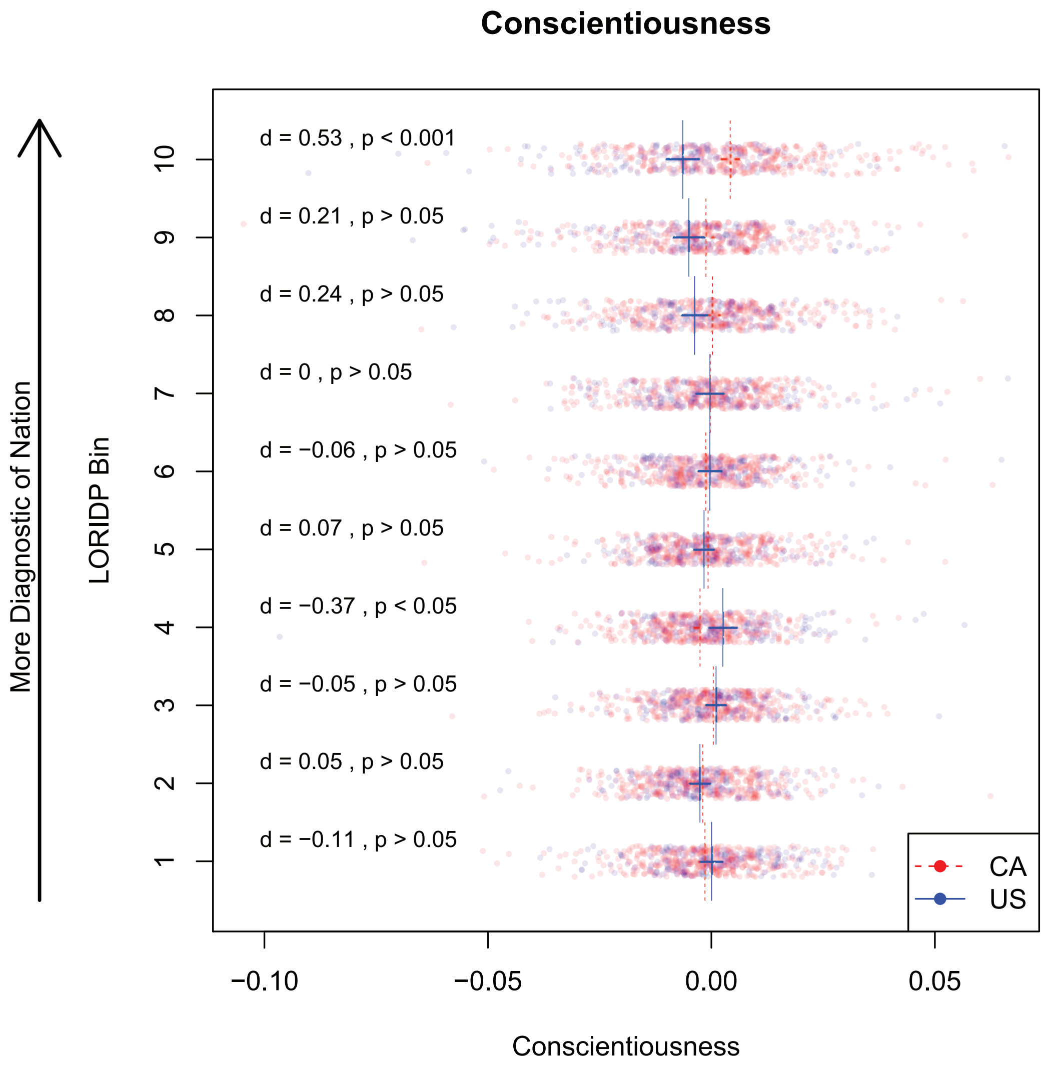

Verticle lines indicate mean conscientiousness of American and Canadian words in each bin. Horizontal lines are 95% confidence intervals of the means. Cohen's d and p-value for t-tests within each bin are reported in the left of the figure. Canadian words are more associated with conscientiousness amongst only the most nationally diagnostic words.

Figure I: Relative association with extraversion of very American (blue) versus very Canadian (red) words

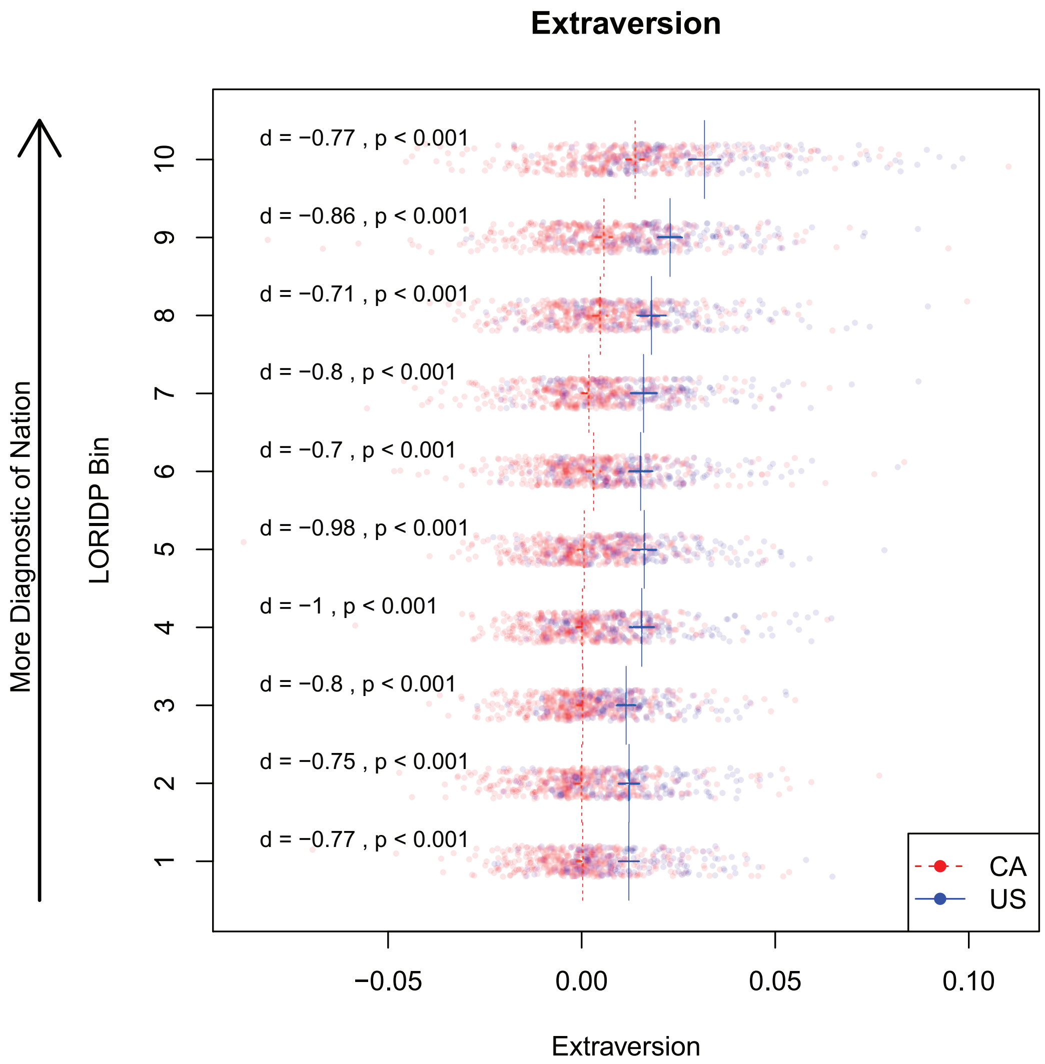

Vertical lines indicate mean extraversion of American and Canadian words in each bin. Horizontal lines are 95% confidence intervals of the means. Cohen's d and p-value for t-tests within each bin are reported in the left of the figure. American words are consistently associated with higher extraversion across the LORIDP distribution.

Figure J: Relative association with agreeableness of very American (blue) versus very Canadian (red) words

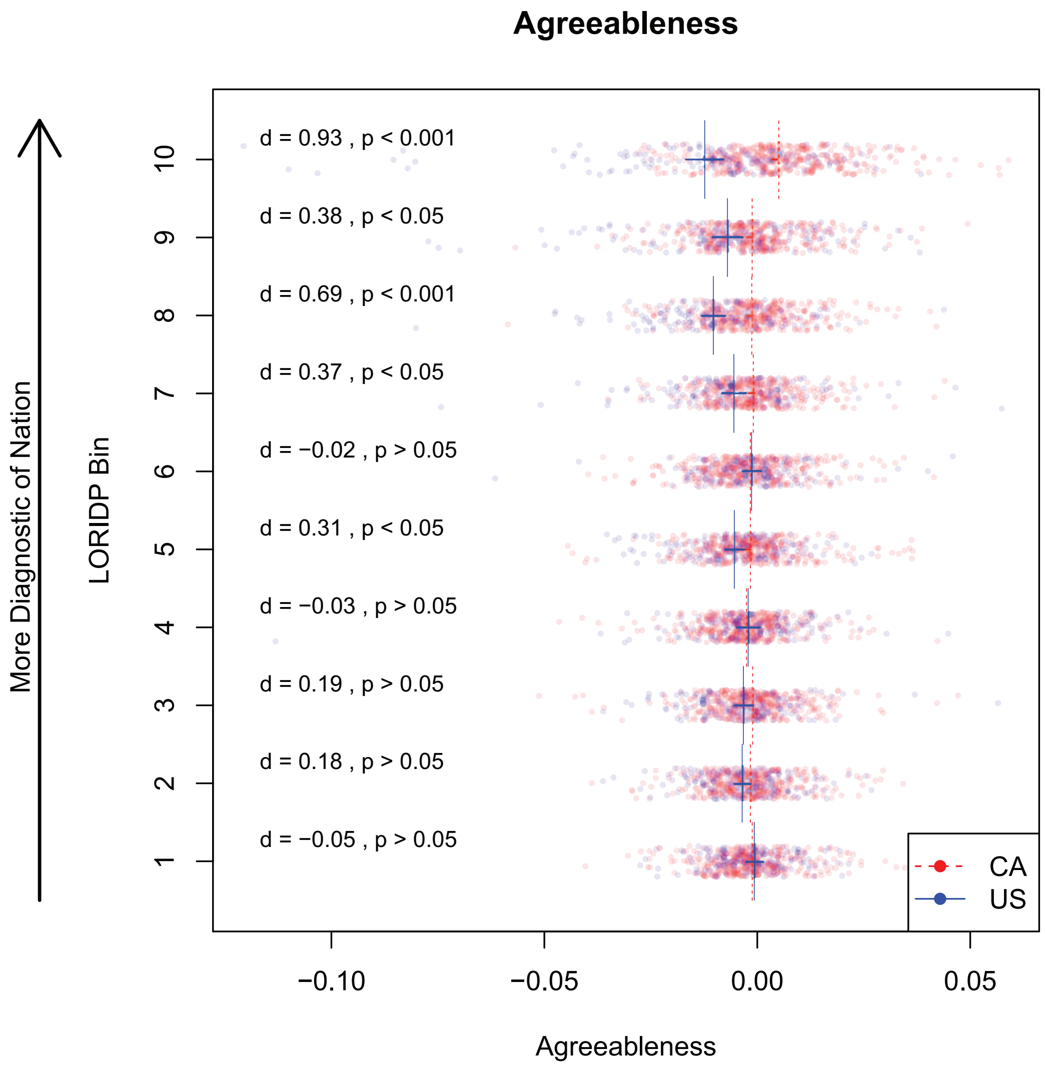

Vertical lines indicate mean agreeableness of American and Canadian words in each bin. Hotizontal lines are 95% confidence intervals of the means. Cohen's d and p-value for t-tests within each bin are reported in the left of the figure. Canadian words are associated with higher agreeableness, amongst the most nationally distinctive words.

Figure K: Relative association with neuroticism of very American (blue) versus very Canadian (red) words

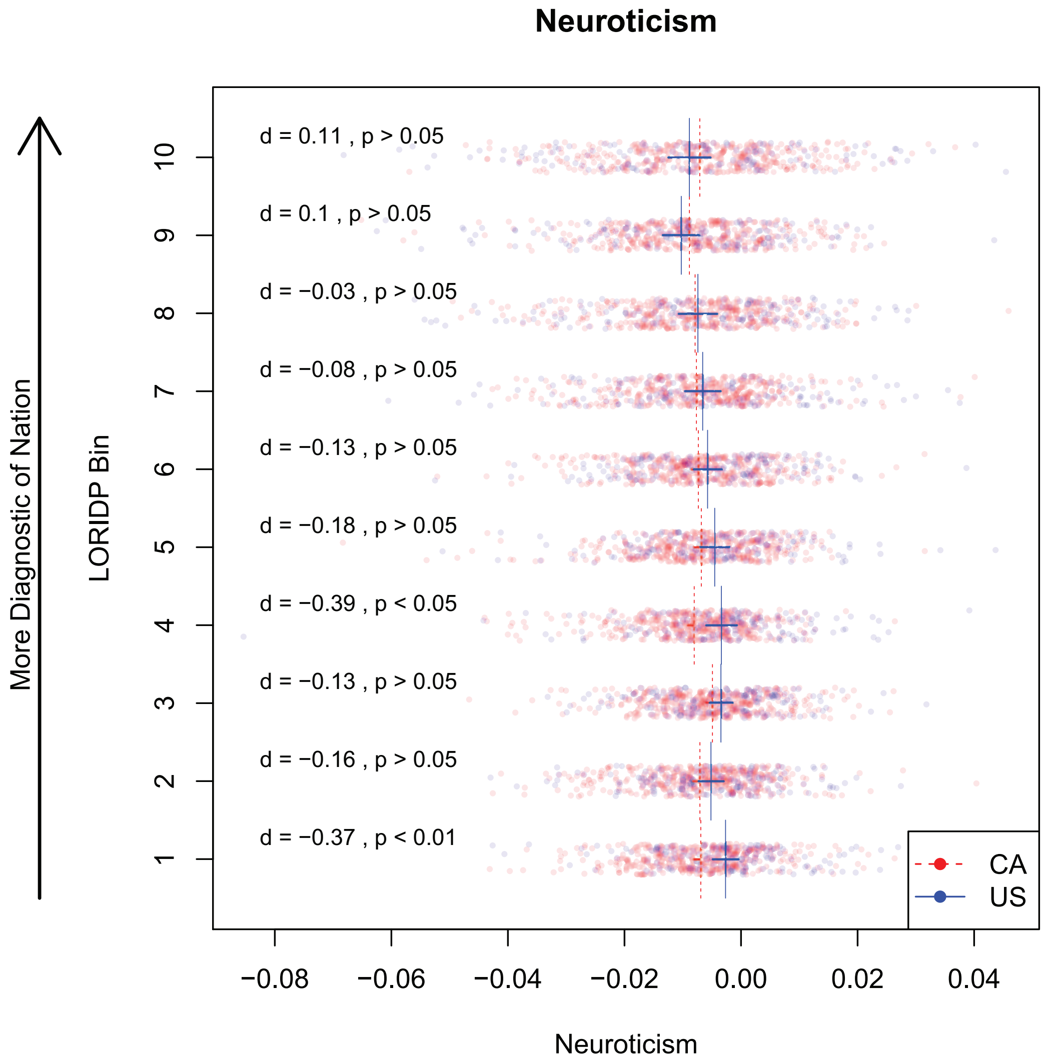

Vertical lines indicate mean neuroticism of American and Canadian words in each bin. Horizontal lines are 95% confidence intervals of the means. Cohen's d and p-value for t-tests within each bin are reported in the left of the figure. Canadian and American words do not have robust differences in neuroticism.

References

[1] Schwartz HA, Eichstaedt JC, Kern ML, Dziurzynski L, Ramones SM, Agrawal M, et al. Personality, gender, and age in the language of social media: The open-vocabulary approach. PloS one. 2013;8(9):e73791.

[2] Youyou W, Kosinski M, Stillwell D. Computer-based personality judgments are more accurate than those made by humans. Proceedings of the National Academy of Sciences. 2015;112(4):1036–1040.

[3] Goldberg LR, Johnson JA, Eber HW, Hogan R, Ashton MC, Cloninger CR, et al. The international personality item pool and the future of public-domain personality measures. Journal of Research in Personality. 2006;40(1):84–96.

[4] Costa PT, Mac Crae RR. Neo Personality Inventory-Revised (NEO PI-R). Psychological Assessment Resources Odessa, FL; 1992.
