# Supplementary material for: National character stereotypes mirror language use: A study of Canadian and American tweets
Source: PLoS One. 2018 Nov 21;13(11):e0206188. doi: 10.1371/journal.pone.0206188 (PMC6248921; doi:10.1371/journal.pone.0206188)
Supplement: S2 File — (PDF) [file pone.0206188.s002.pdf]

S2

One possible issue with our finding that national language use mirrors national character stereotypes is that our comparisons up to this point have been based on the total linguistic output of Canada and the United States on Twitter. For our claims to hold, comparisons of smaller, and ideally geographically adjacent areas, should follow the trends established in our comparisons of the nations as a whole. In this study we focused on an area of land on the West Coast of Canada and the USA. We considered all US and Canadian Tweets that were sent within the following bounding box area, which was based on the following coordinates: latitude 47.06534, 49.53447; longitude: -125.93, -121.8276. Figure **??** shows a map of the area included in this bounding box; Seattle and Vancouver are included within its area.

686 words passed the critical Bonferroni-corrected z-score at the lower tail of the LORIDP distribution, i.e. Canadian words, and 495 words passed the critical Bonferroni-corrected z-score at the upper tail of the LORIDP distribution, i.e. US words. Figure B shows the most diagnostic words for each country at the west coast. Similar to the countries as a whole, Canadian words are more positive than American words in most LORIDP bins. The openness difference is not robust in this smaller data set, though Canadian words skew more open. The most Canadian words are more associated with conscientiousness than the most American words.

Figure A: Area of our analysis of the West Coast

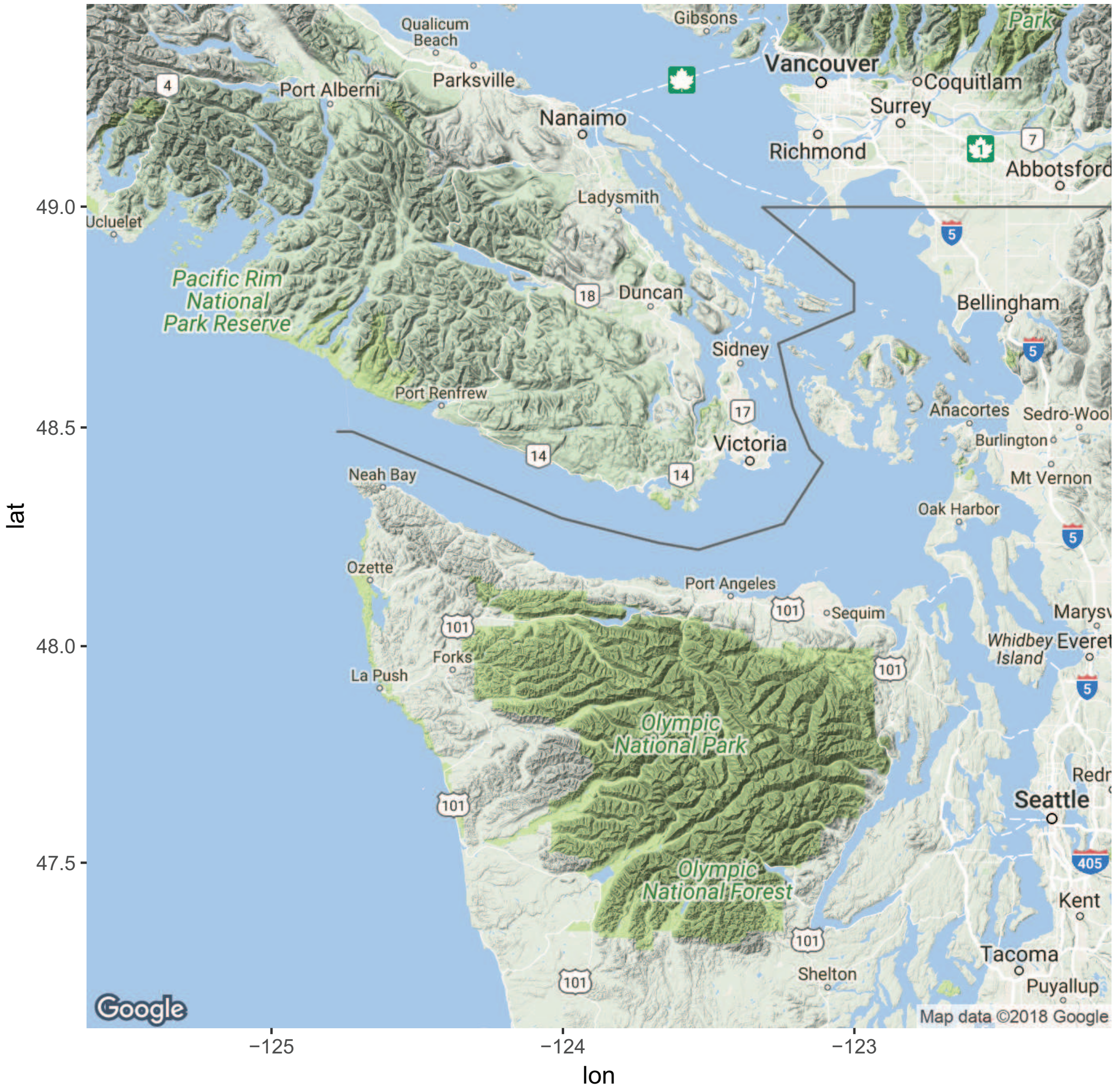

The area contained in bounding box used in our comparison of the Canadian and US west coast.

Figure B: Most Canadian (red) and Most American (blue) words at the West Coast

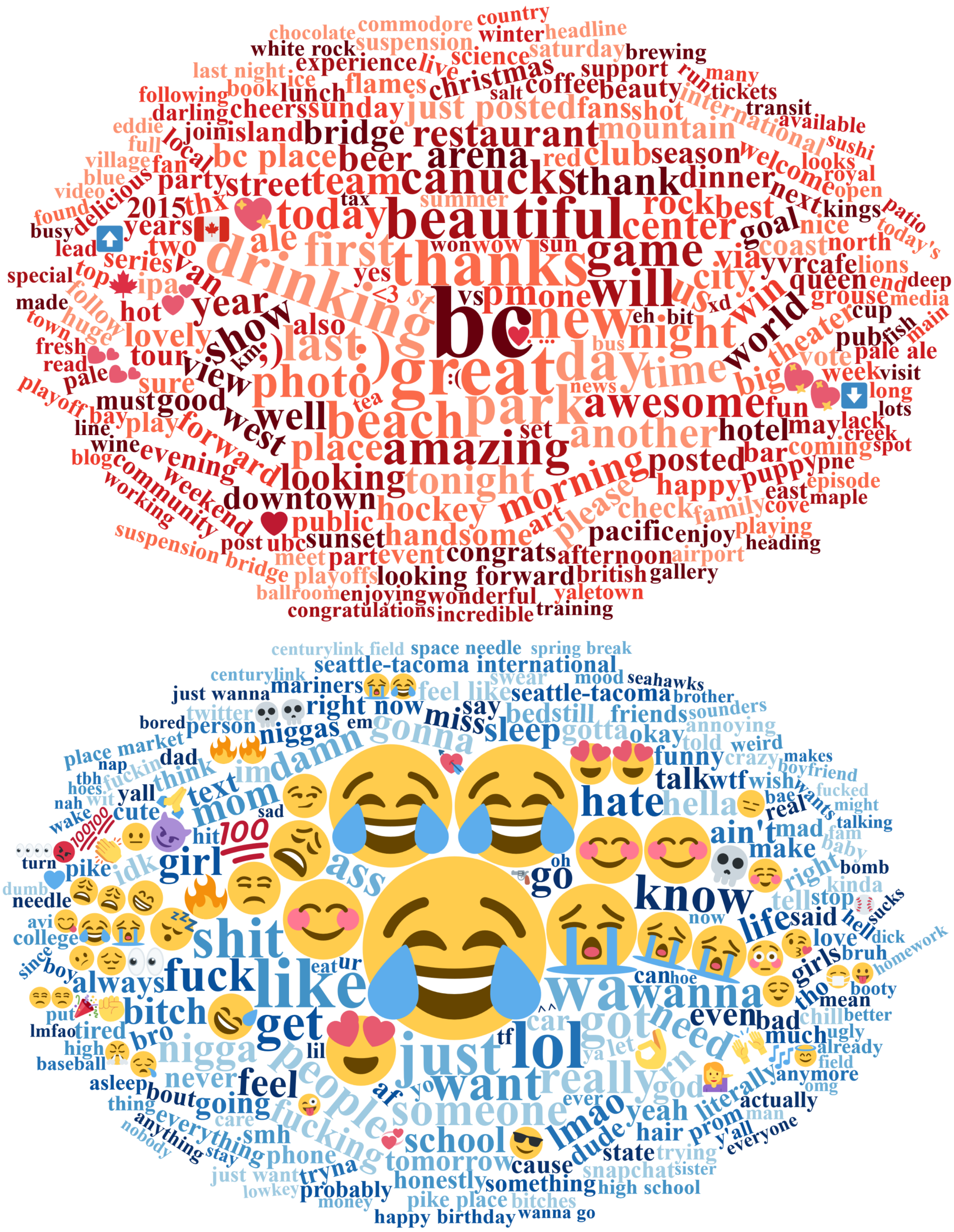

The top 250 US and Canadian words at the west coast. Text size is proportional to LORIDP z-score. Colour is for readability.

Figure C: Relative Positivity of very American (blue) versus very Canadian (red) words in the west coast region

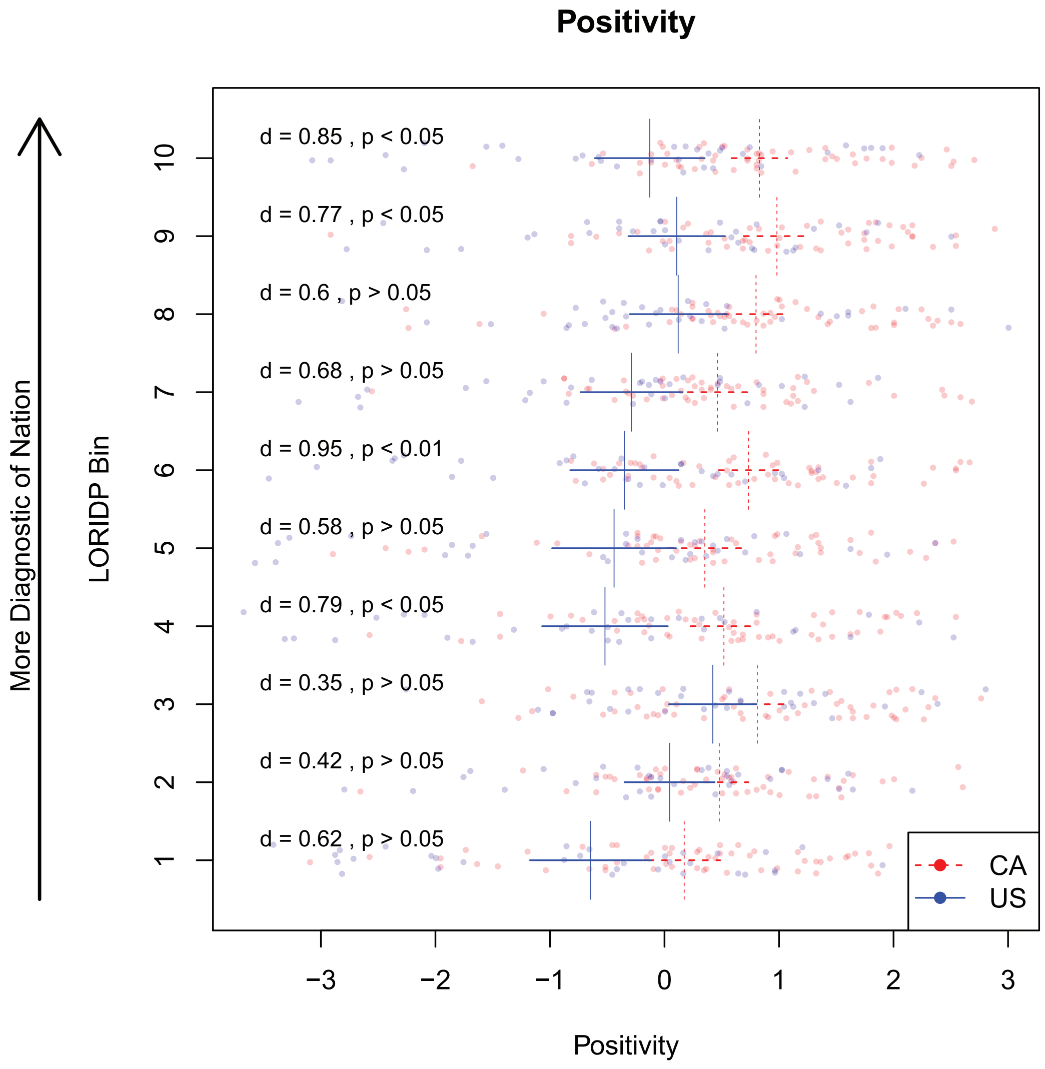

Dashed lines indicate mean positivity of American and Canadian words in each bin. Black lines are 95% confidence intervals of the means. Cohen's D and p-values for t-tests within each bin are reported in the left of the figure.

Figure D: Relative association with openness of very American (blue) versus very Canadian (red) words in the west coast region

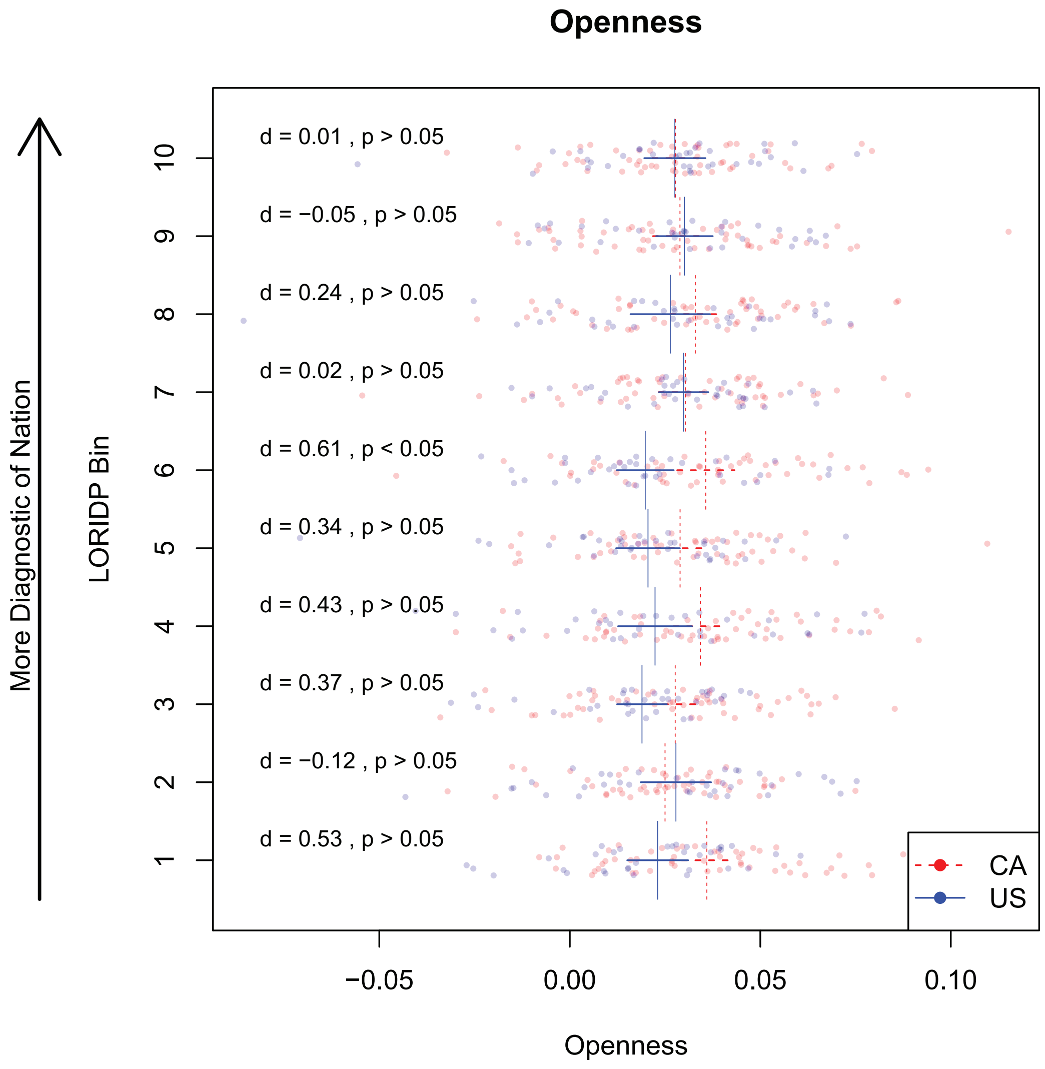

Dashed lines indicate mean openness of American and Canadian words in each bin. Black lines are 95% confidence intervals of the means. Cohen's D and p-value for t-tests within each bin are reported in the left of the figure.

Figure E: Relative association with conscientiousness of very American (blue) versus very Canadian (red) words in the west coast region

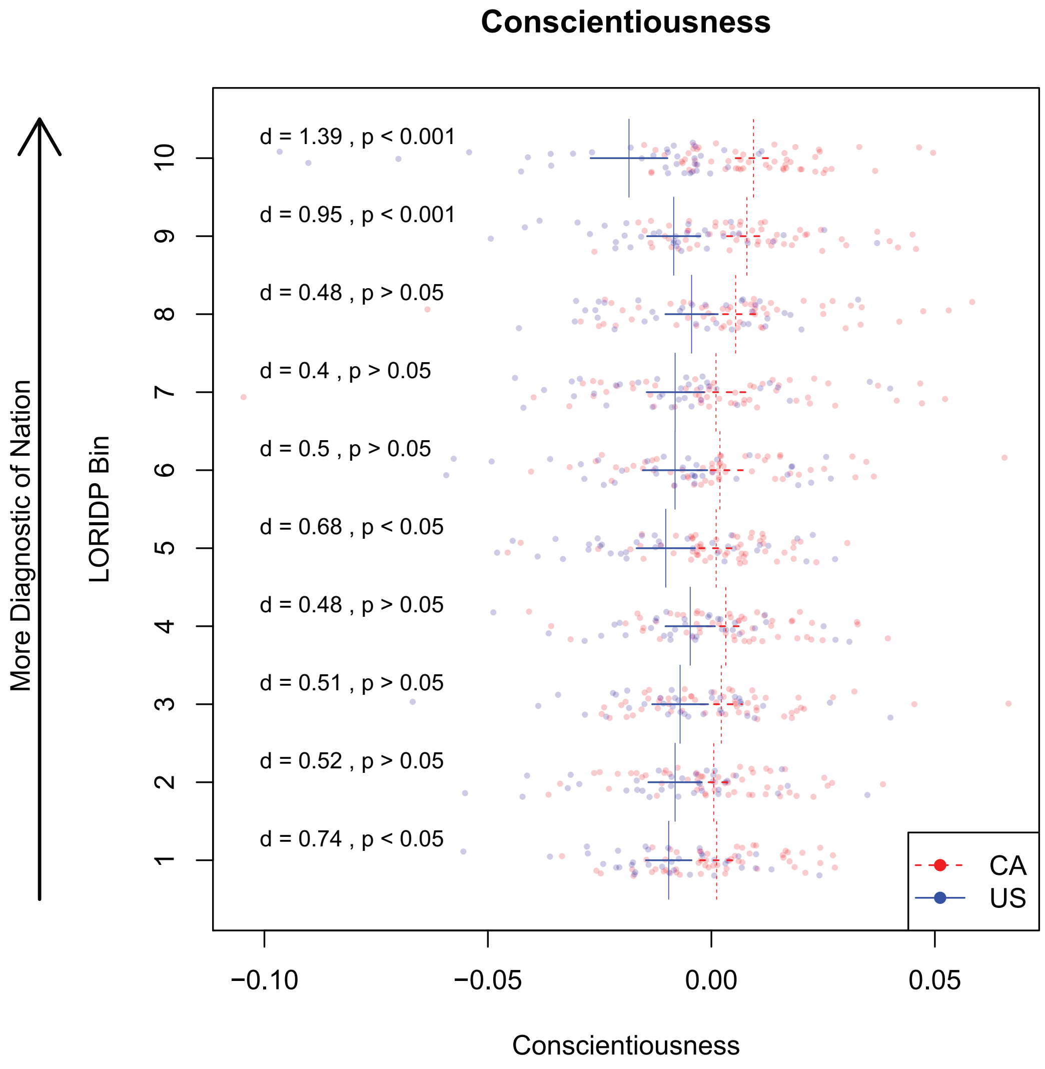

Dashed lines indicate mean conscientiousness of American and Canadian words in each bin. Black lines are 95% confidence intervals of the means. Cohen's D and p-value for t-tests within each bin are reported in the left of the figure.

Figure F: Relative association with extraversion of very American (blue) versus very Canadian (red) words in the west coast region

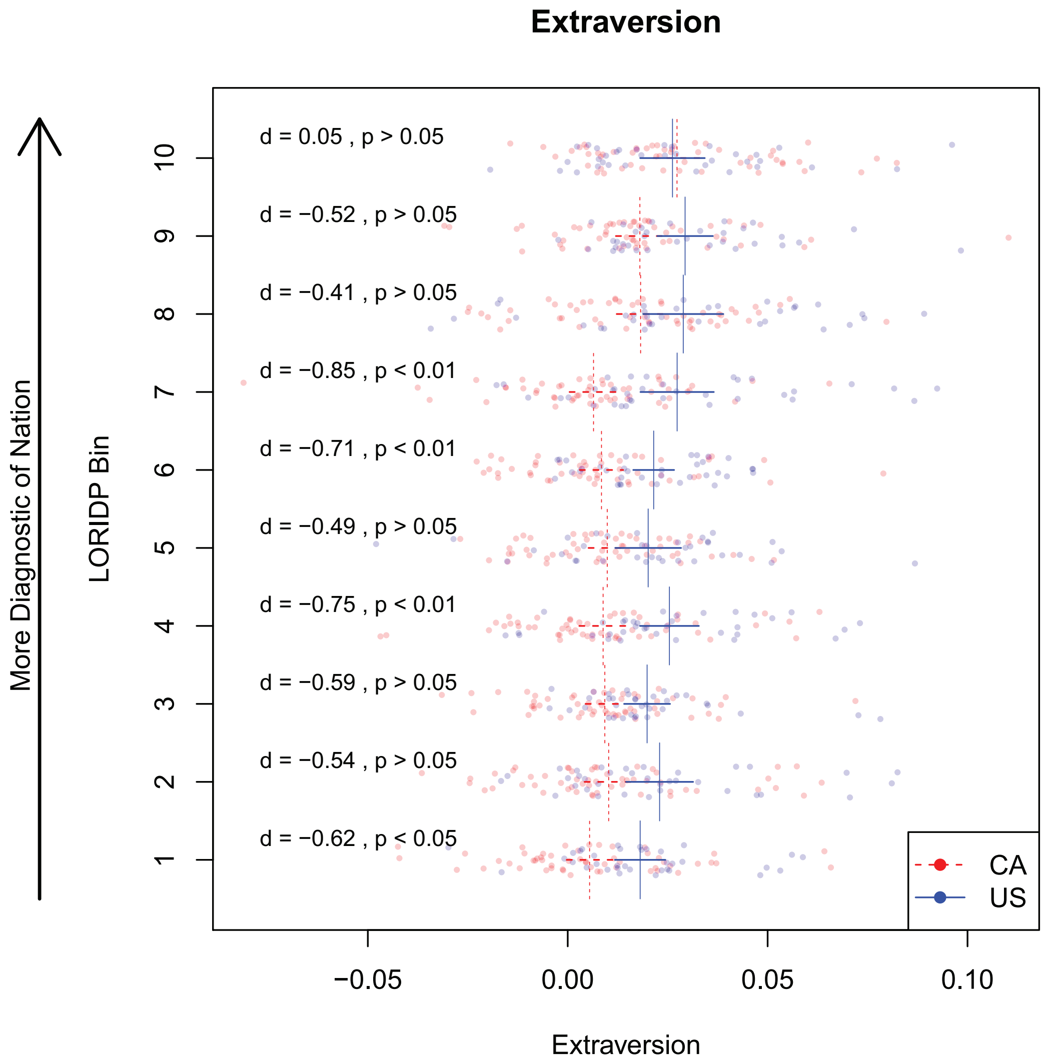

Dashed lines indicate mean extraversion of American and Canadian words in each bin. Black lines are 95% confidence intervals of the means. Cohen's D and p-value for t-tests within each bin are reported in the left of the figure.

Figure G: Relative association with agreeableness of very American (blue) versus very Canadian (red) words in the west coast region

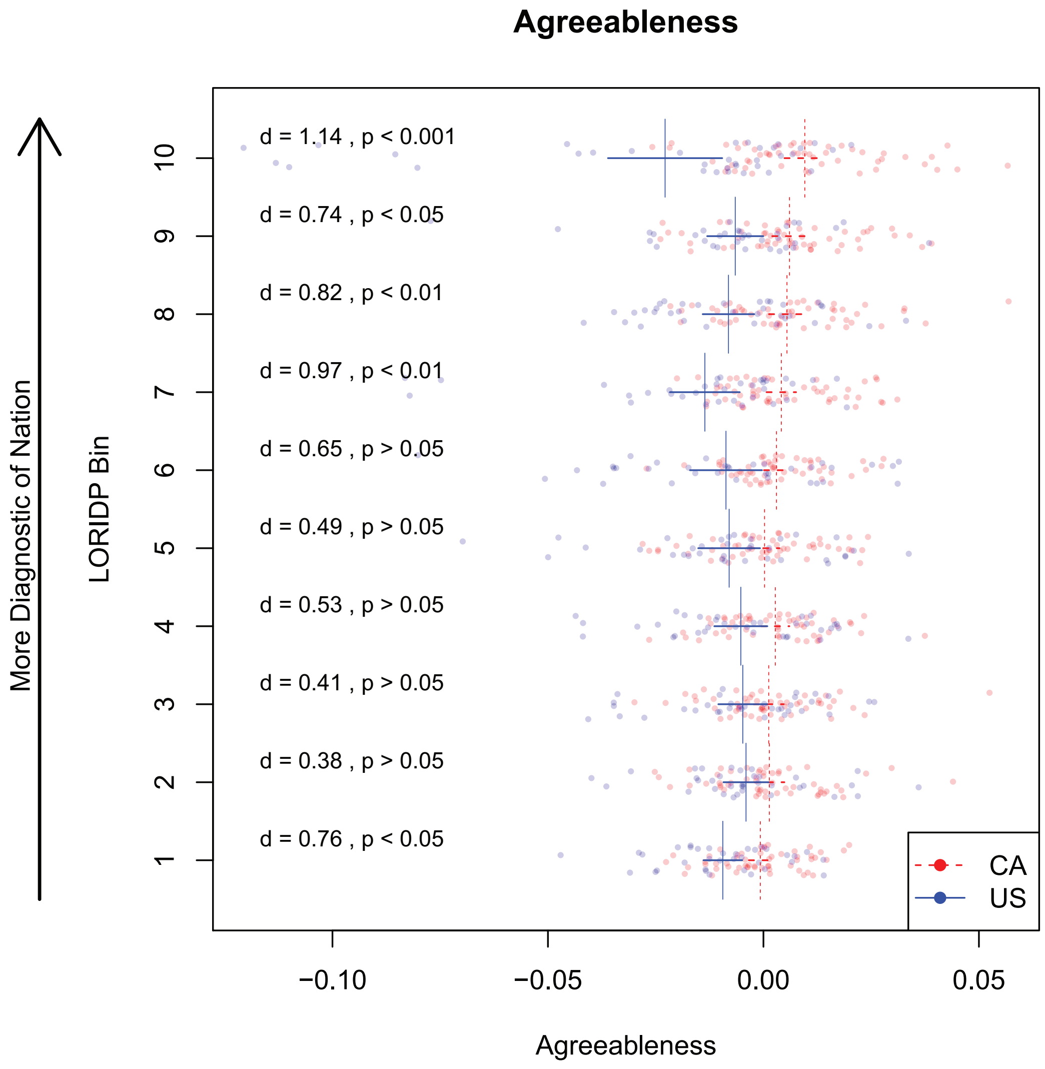

Dashed lines indicate mean agreeableness of American and Canadian words in each bin. Black lines are 95% confidence intervals of the means. Cohen's D and p-value for t-tests within each bin are reported in the left of the figure.

Figure H: Relative association with neuroticism of very American (blue) versus very Canadian (red) words in the west coast region

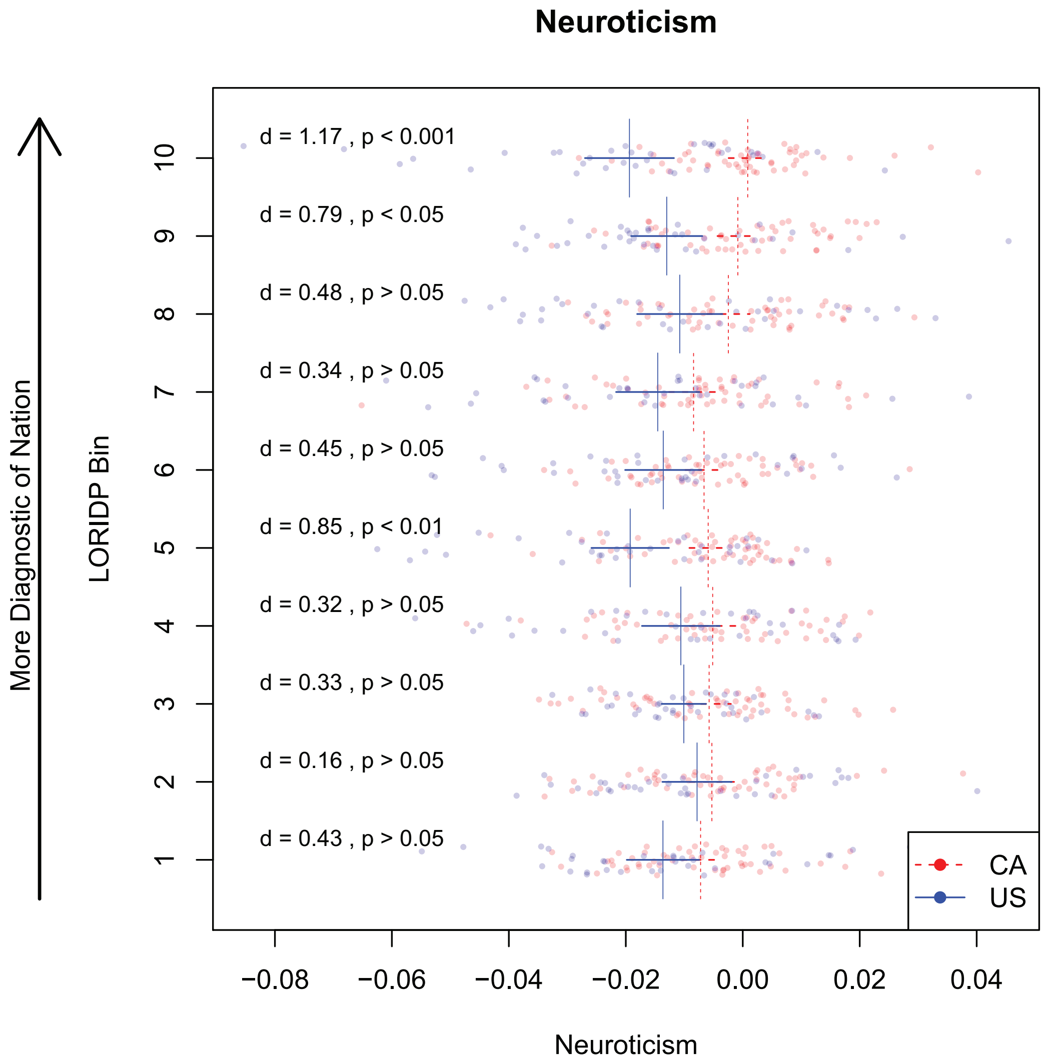

Dashed lines indicate mean neuroticism of American and Canadian words in each bin. Black lines are 95% confidence intervals of the means. Cohen's D and p-value for t-tests within each bin are reported in the left of the figure.
