# Supplementary material for: National character stereotypes mirror language use: A study of Canadian and American tweets
Source: PLoS One. 2018 Nov 21;13(11):e0206188. doi: 10.1371/journal.pone.0206188 (PMC6248921; doi:10.1371/journal.pone.0206188)
Supplement: S3 File — (PDF) [file pone.0206188.s003.pdf]

S3

In this study we focused on an area of land in the eastern part of Canada and the USA (encompassing the areas surrounding Lake Erie and Lake Ontario). 4459 words exceeded the Bonferroni corrected threshold for Canada and 2536 words exceeded the Bonferroni corrected threshold for the US. This involved focusing on any tweets that were sent within the following bounding box; latitude 40.797177, 44.465151; longitude: -85.935059, -75.344238. This area includes the Greater Toronto area, as well as Cleveland, Detroit, Buffalo, and Pittsburgh. Figures C to H present plots of the strength of association of Canadian and American words with personality traits and positivity within this region. Regional trends are consistent with national trends. Canadian words are robustly less associated with extraversion. Canadian words are more associated with agreeableness and conscientiousness among the most nationally diagnostic words. Canadian words are more associated with openness among the lower LORIDP bins. There is no difference in neuroticism.

686 words passed the critical Bonferroni-corrected z-score at the lower tail of the LORIDP distribution, i.e. Canadian words, and 495 words passed the critical Bonferroni-corrected z-score at the upper tail of the LORIDP distribution, i.e. US words. Figure **??** shows the most diagnostic words for each country at the eastern region. Similar to the countries as a whole, Canadian words are more positive than American words in most LORIDP bins. The openness difference is not robust in this smaller data set, though Canadian words skew more open. The most Canadian words are more associated with conscientiousness than the most American words.

Figure A: Area of our analysis of the eastern region

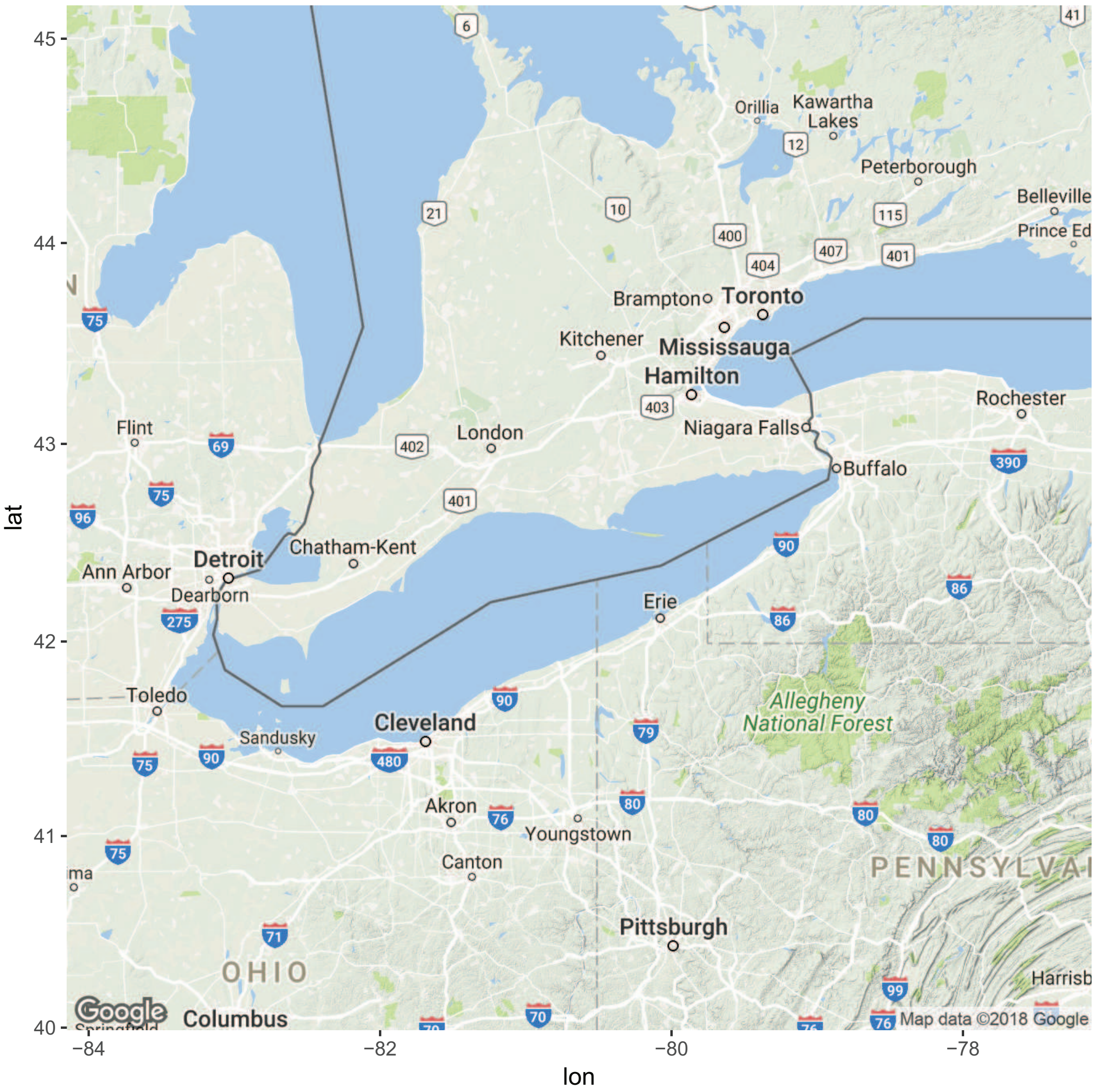

The area contained in bounding box used in our comparison of the Canadian and US eastern region.

Figure B: Most Canadian (red) and Most American (blue) words at the eastern region

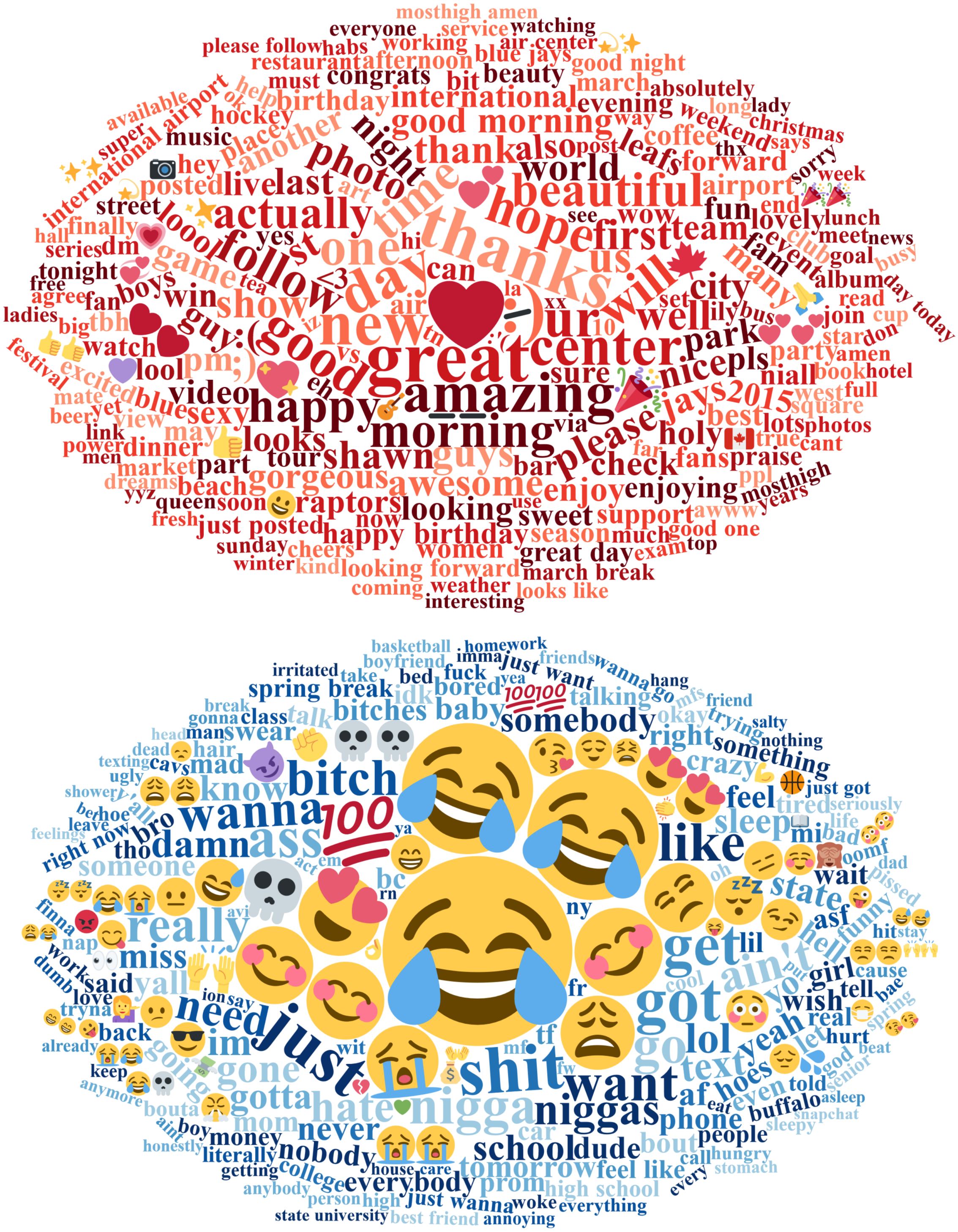

The top 250 US and Canadian words at the eastern region. Text size is proportional to LORIDP z-score. Colour is for readability.

Figure C: Relative Positivity of very American (blue) versus very Canadian (red) words in the eastern region

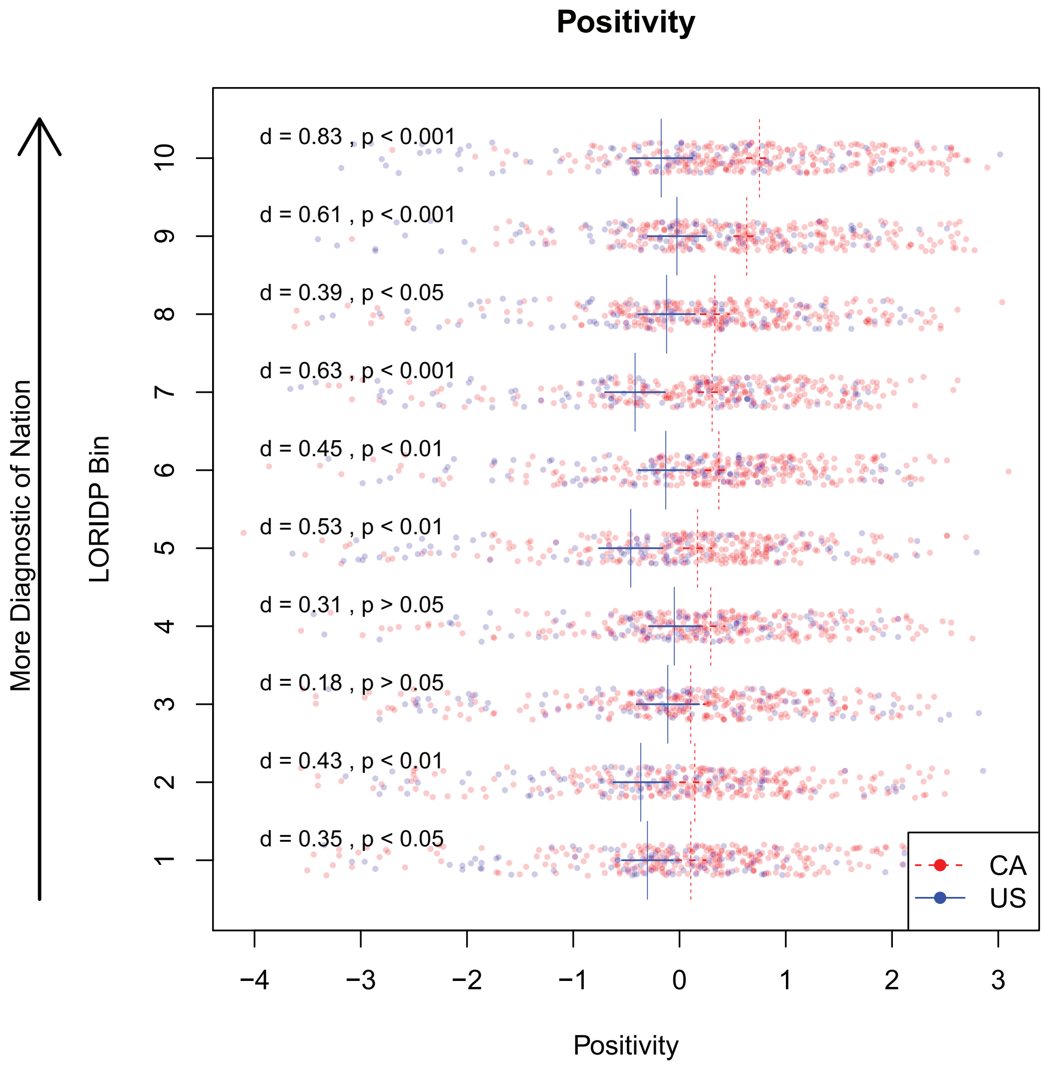

Dashed lines indicate mean positivity of American and Canadian words in each bin. Black lines are 95% confidence intervals of the means. Cohen's D and p-values for t-tests within each bin are reported in the left of the figure.

Figure D: Relative association with openness of very American (blue) versus very Canadian (red) words in the eastern region

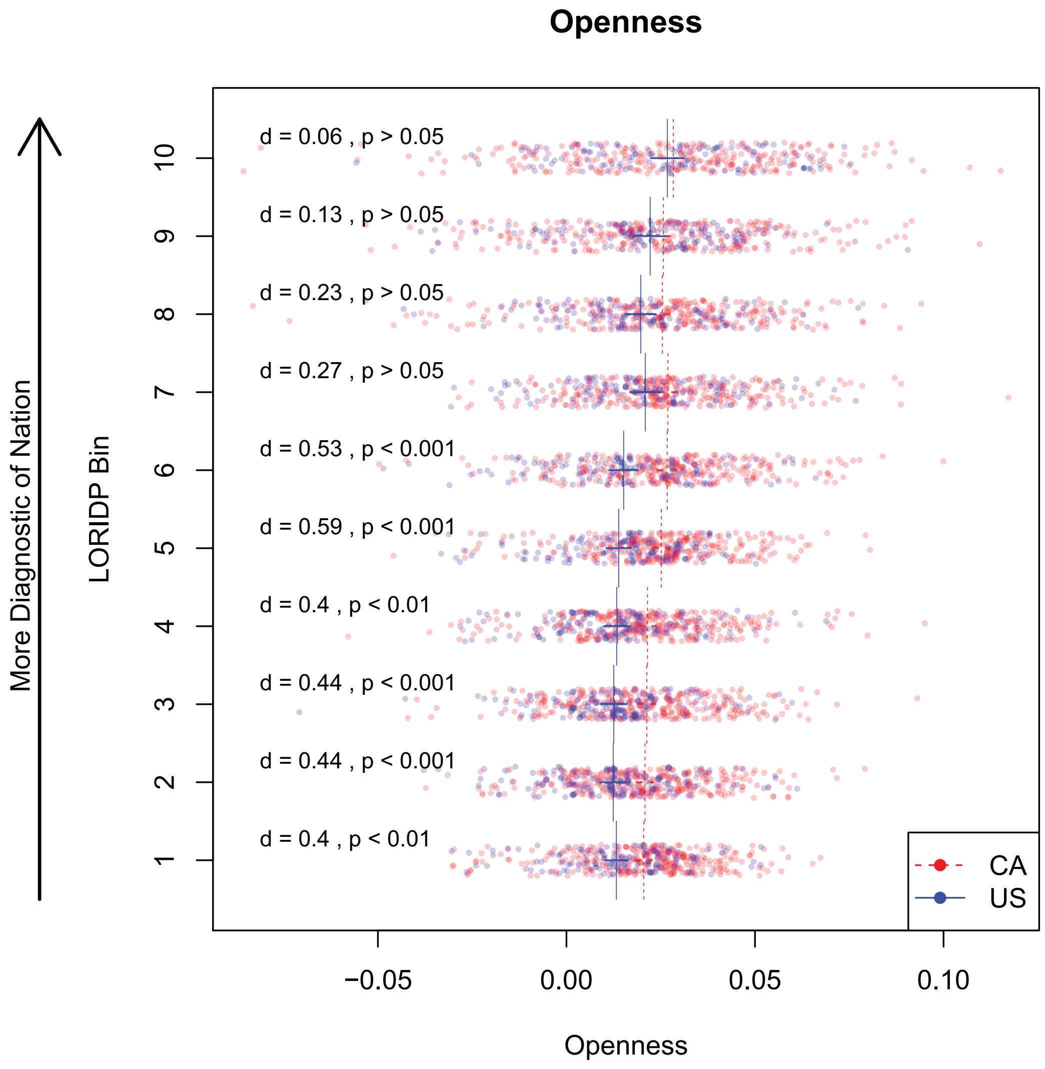

Dashed lines indicate mean openness of American and Canadian words in each bin. Black lines are 95% confidence intervals of the means. Cohen's D and p-value for t-tests within each bin are reported in the left of the figure.

Figure E: Relative association with conscientiousness of very American (blue) versus very Canadian (red) words in the eastern region

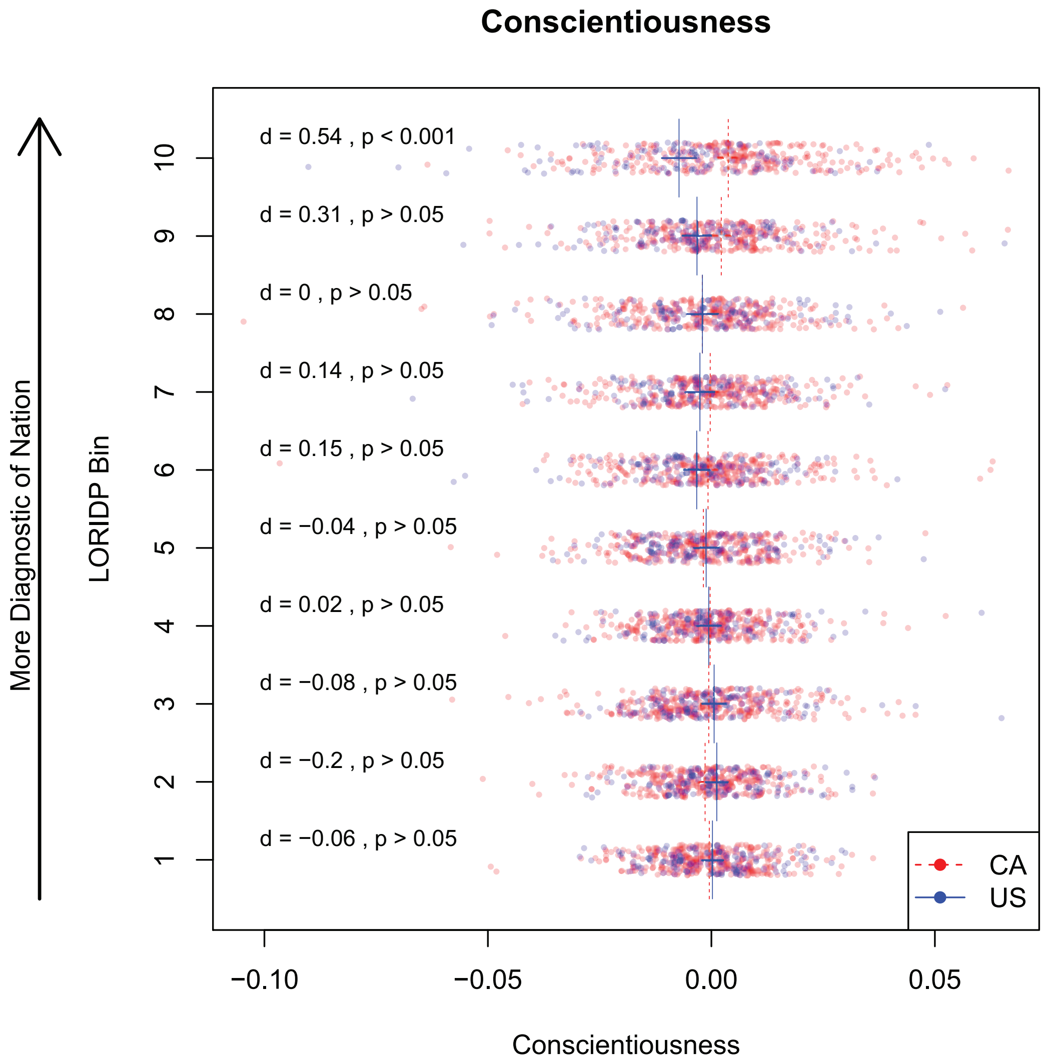

Dashed lines indicate mean conscientiousness of American and Canadian words in each bin. Black lines are 95% confidence intervals of the means. Cohen's D and p-value for t-tests within each bin are reported in the left of the figure.

Figure F: Relative association with extraversion of very American (blue) versus very Canadian (red) words in the eastern region

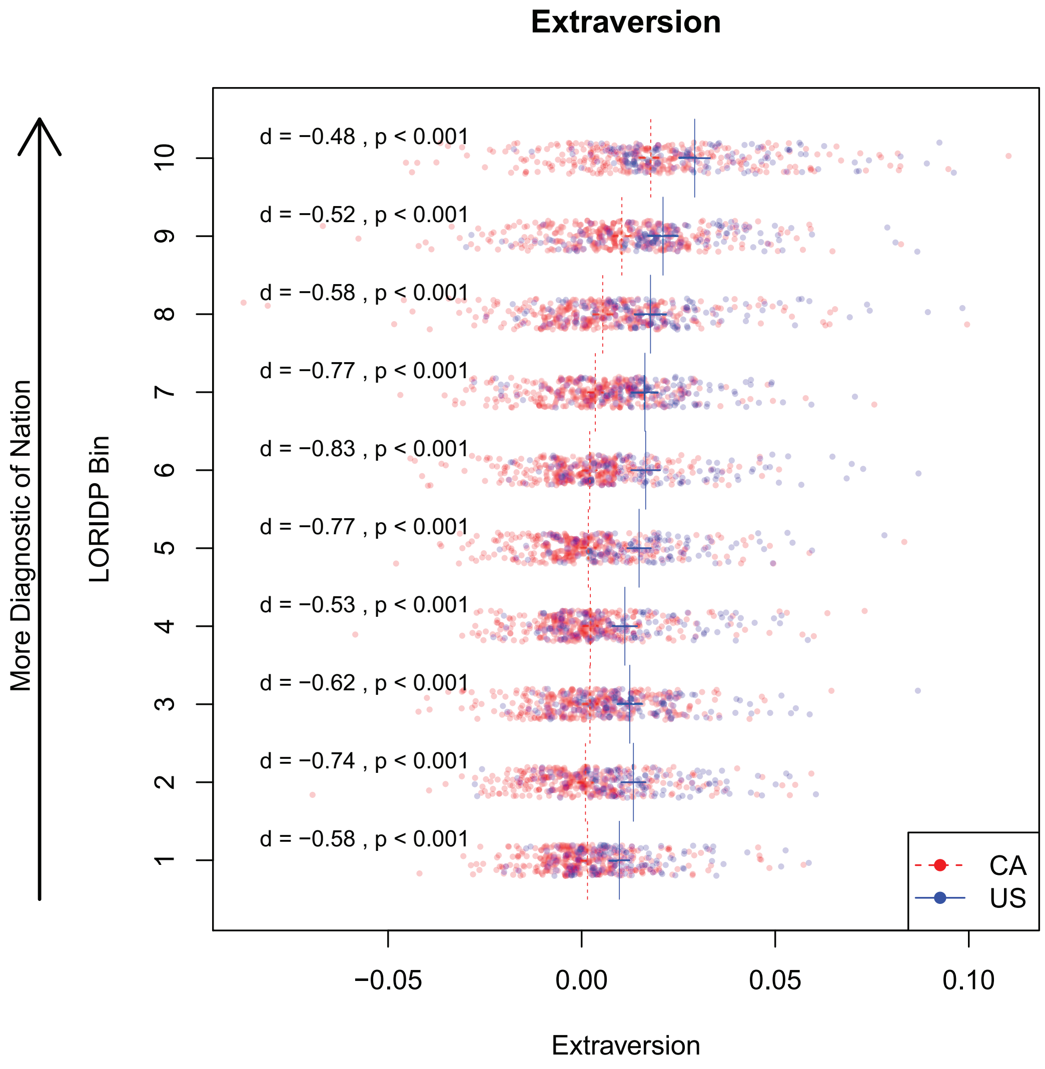

Dashed lines indicate mean extraversion of American and Canadian words in each bin. Black lines are 95% confidence intervals of the means. Cohen's D and p-value for t-tests within each bin are reported in the left of the figure.

Figure G: Relative association with agreeableness of very American (blue) versus very Canadian (red) words in the eastern region

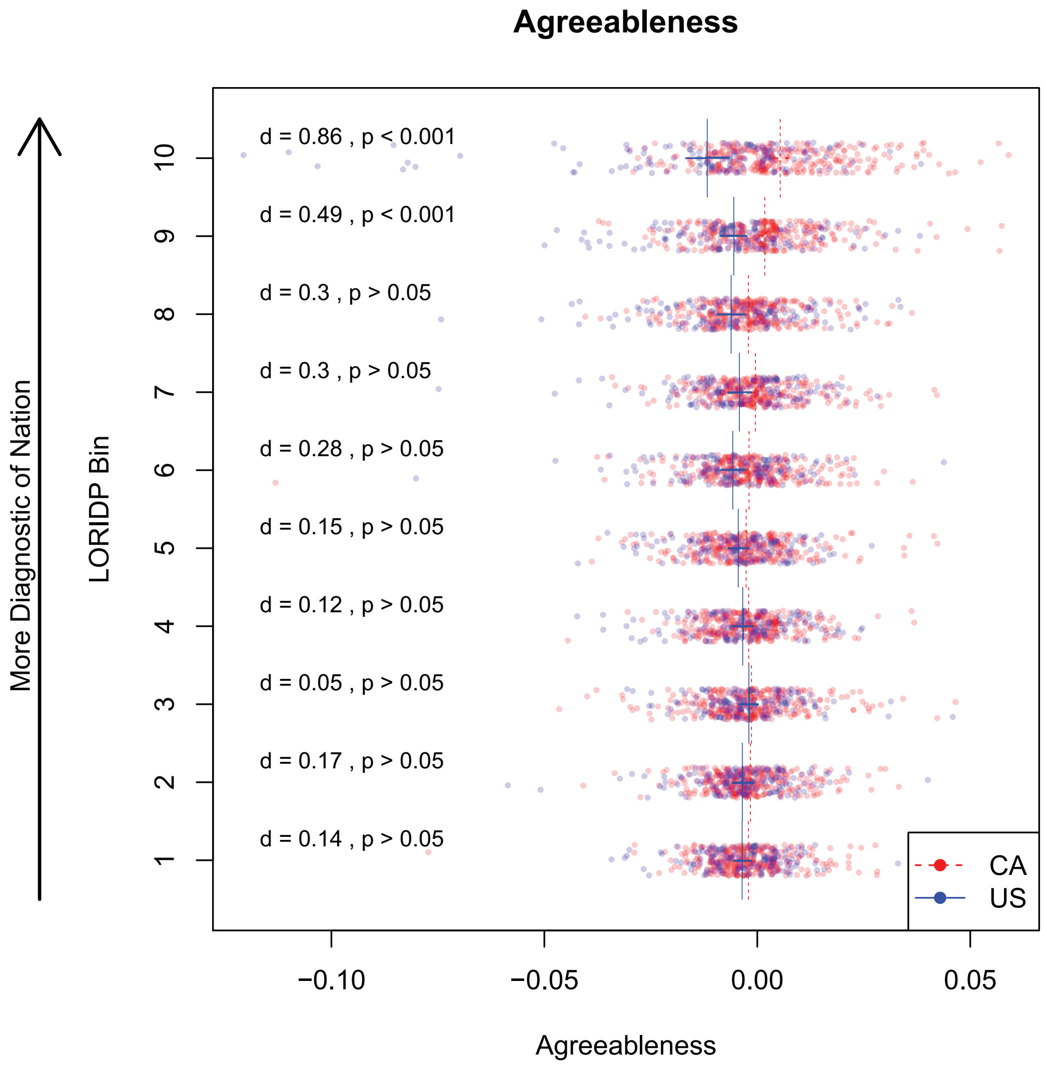

Dashed lines indicate mean agreeableness of American and Canadian words in each bin. Black lines are 95% confidence intervals of the means. Cohen's D and p-value for t-tests within each bin are reported in the left of the figure.

Figure H: Relative association with neuroticism of very American (blue) versus very Canadian (red) words in the eastern region

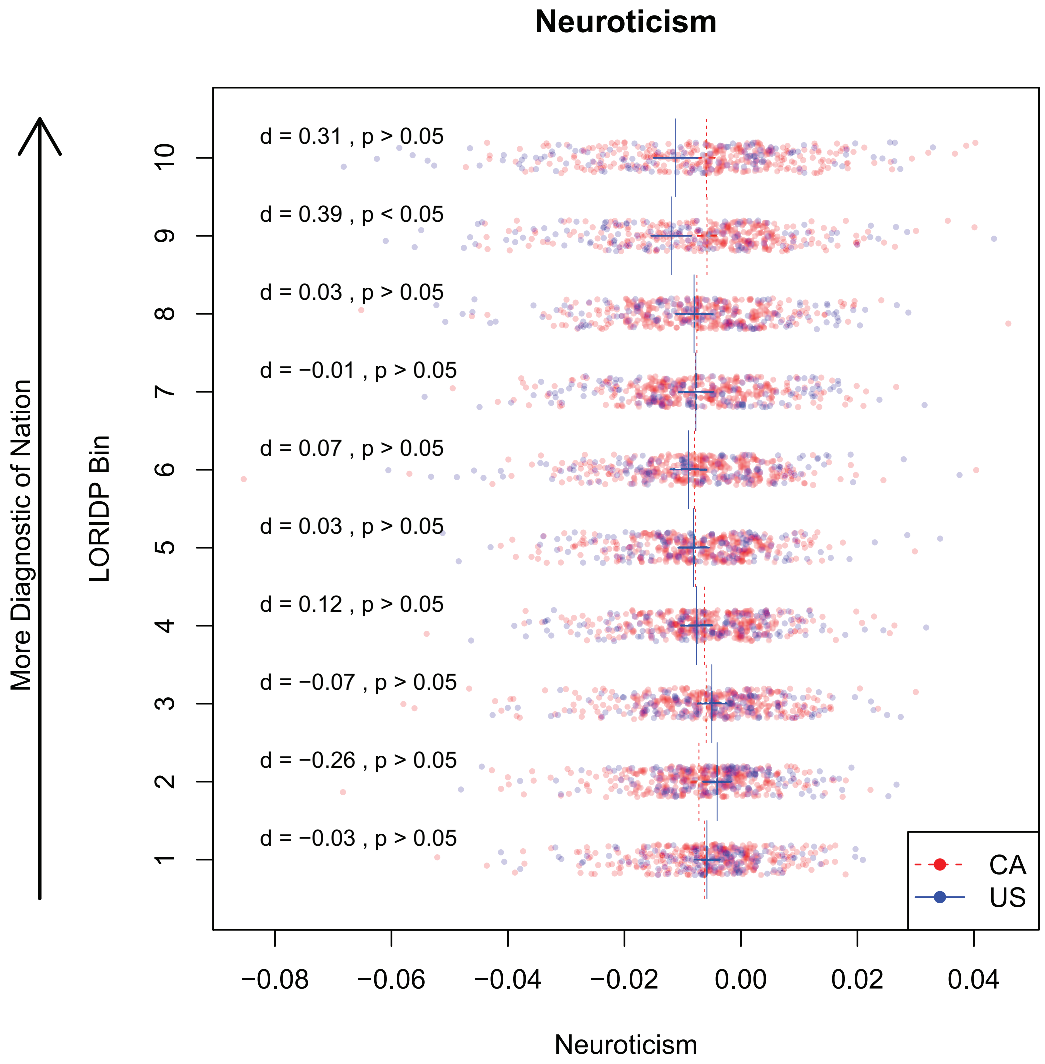

Dashed lines indicate mean neuroticism of American and Canadian words in each bin. Black lines are 95% confidence intervals of the means. Cohen's D and p-value for t-tests within each bin are reported in the left of the figure.
