# Supplementary material for: National character stereotypes mirror language use: A study of Canadian and American tweets
Source: PLoS One. 2018 Nov 21;13(11):e0206188. doi: 10.1371/journal.pone.0206188 (PMC6248921; doi:10.1371/journal.pone.0206188)
Supplement: S4 File — (PDF) [file pone.0206188.s004.pdf]

S4

Quantitative analysis of word use is complicated by the extreme skew of the word frequency distribution. Word frequencies approximately follow a Zipf distribution, where the 2nd most frequent word will occur about half-as often as the most frequent word, the 3rd most frequent word will occur about half-as often as the 2nd most frequent, and so on. The lower ranks of a word frequency distribution will contain rare words with a frequency of 1. Naive application of effect size statistics for counts, such as the log-odds ratio, will tend to overestimate the importance of differences in frequency of rare words, and underestimate the importance of differences in frequency among highly frequent words. By modifying the log-odds ratio to include an estimate of the variance in a word's frequency, and incorporating a prior count for words, LORIDP is sensitive to difference among higher frequency words, and also does not overestimate the importance of rare words.

To illustrate these advantages of LORIDP, we generate artificial corpora with no differences in frequency and with difference in word frequency, and compare LORIDP to the unmodified log-odds ratio. Each corpus contains 20 "word" types (denoted W0 to W19, from most frequent to least frequent) and around 20,000 word tokens, in a Zipf distribution. To avoid many infinite or not-a-number results, frequencies have some minor random variation (a random integer from 1 to 5 added/subtracted to counts). The significance threshold for LORIDP we have chosen for this example is the same threshold we use in our Twitter analysis in the main paper: $p < 0.01$ Bonferroni corrected for the number of comparisons. In this case there are 20 comparisons, so the significance threshold is $|z| > 3.48$. In each example, the background corpus is set as the sum of corpus A and corpus B, representing a case where a corpus is exhaustively split into two sub-corpora, as is the case for our Twitter analysis.

Table A shows two simulated corpora where there are no large differences in word frequency. The unmodified log odds ratios tend to become larger when reaching the low-frequency words in the tail of the word frequency distribution, while LORIDP z-scores do not increase over the word frequency distribution. Table B shows two simulated corpora where there is a small (difference of about 50) difference in counts between the two highest frequency words. Incorporating a prior count for these words into the calculation means LORIDP treats this difference as significant ($z = 4.24$;-3.65), while the unmodified log odds ratio is barely different from zero. Table C shows an example where the same difference in counts (50) occurs between a high and a mid frequency word. LORIDP treats this

difference among both words as significant (z = 4.12;-3.47), whereas the log-odds ratio is larger for the mid-frequency word (log odds ratio = -0.44) than the high frequency word (log odds ratio = 0.00). Differences amongst some rare words in this example still recieve larger effect sizes by the unmodified log odds ratio. Table D shows an example where the absolute difference in frequency between the words has changed, but the overall size of the corpora much larger (2,000,000 words versus 20,000). Increasing the corpus size means the log-odds ratio falls for the mid-frequency word. LORIDP on the other hand, still treats these differences in counts as significant (z = 6.04;-4.67). LORIDP provides a clear statistic for how words are over/under represented in two corpora that is not distorted by the word's position in the word frequency distribution or corpus size.

Table A: Two corpora lacking systematic differences in word frequency.

Word	Corpus A	Corpus B	Difference	LORIDP	Log Odds Ratio
W0	10003	9996	7	1.23	0.00
W1	4997	4998	-1	-1.58	-0.00
W2	2505	2502	3	0.96	0.00
W3	1246	1249	-3	-1.40	-0.00
W4	627	625	2	0.92	0.00
W5	315	310	5	1.57	0.02
W6	157	161	-4	-1.44	-0.03
W7	84	79	5	1.58	0.06
W8	38	42	-4	-1.42	-0.10
W9	23	19	4	1.41	0.19
W10	8	8	0	-0.05	-0.00
W11	6	2	4	1.40	1.10
W12	5	8	-3	-1.22	-0.47
W13	5	1	4	1.39	1.61
W14	1	4	-3	-1.21	-1.39
W15	1	0	1	0.68	Inf
W16	1	4	-3	-1.21	-1.39
W17	1	5	-4	-1.39	-1.61
W18	0	0	0	0	0
W19	1	0	1	0.68	Inf

LORIDP remains below the |z| = 3.48 threshold over the entire word frequency distribution. The log-odds ratio tends to grow for rarer words.

Table B: Two corpora with differences in two high frequency words (bold rows).

Word	Corpus A	Corpus B	Difference	LORIDP	Log Odds Ratio
**W0**	**9997**	**9979**	**18**	**4.24**	**0.00**
**W1**	**5002**	**5022**	**-20**	**-3.65**	**-0.01**
W2	2497	2497	0	0	0.00
W3	1249	1254	-5	-1.63	-0.00
W4	624	621	3	1.24	0.00
W5	309	314	-5	-1.59	-0.02
W6	161	155	6	1.74	0.04
W7	83	83	0	0	0.00
W8	45	44	1	0.71	0.02
W9	19	20	-1	-0.71	-0.05
W10	7	9	-2	-1.00	-0.25
W11	5	4	1	0.71	0.22
W12	3	3	0	0	0.00
W13	6	7	-1	-0.71	-0.15
W14	2	0	2	0.96	Inf
W15	5	0	5	1.52	Inf
W16	1	0	1	0.68	Inf
W17	1	4	-3	-1.21	-1.39
W18	1	2	-1	-0.70	-0.69
W19	1	0	1	0.68	Inf

W0 has 18 more occurences in corpus A and W1 has 20 fewer occurences compared to corpus A. An unmodified log-odds ratio treats these differences as very small, whereas these difference pass the $|z| = 3.48$ significance threshold for LORIDP.

Table C: Two corpora with differences in a high (W0) and low (W8) frequency word.

Word	Corpus A	Corpus B	Difference	LORIDP	Log Odds Ratio
**W0**	**10000**	**9972**	**28**	**4.12**	**0.00**
W1	4999	5003	-4	-2.52	-0.00
W2	2505	2496	9	1.89	0.00
W3	1250	1247	3	0.93	0.00
W4	623	621	2	0.82	0.00
W5	314	314	0	-0.42	-0.00
W6	156	152	4	1.39	0.03
W7	81	77	4	1.40	0.05
**W8**	**44**	**68**	**-24**	**-3.47**	**-0.44**
W9	21	17	4	1.41	0.21
W10	10	12	-2	-1.00	-0.18
W11	5	5	0	-0.05	-0.00
W12	7	1	6	1.69	1.95
W13	0	1	-1	-0.68	-Inf
W14	1	3	-2	-0.99	-1.10
W15	3	0	3	1.18	Inf
W16	3	3	0	-0.04	-0.00
W17	1	1	0	-0.02	-0.00
W18	1	3	-2	-0.99	-1.10
W19	0	6	-6	-1.67	-Inf

LORIDP successfully captures this difference in W0 and W8, whereas the log odds ratio treats the difference between corpora for W0 as very small, and some log odds ratios for very rare words are larger.

Table D: Two corpora with differences in frequency in a high and low frequency word, and the corpus sizes are much larger (2,000,000 instead of 20,000).

Word	Corpus A	Corpus B	Difference	LORIDP	Log Odds Ratio
**W0**	**1000003**	**999979**	**24**	**6.04**	**0.00**
W1	499999	500004	-5	0.91	0.00
W2	249998	249996	2	1.71	0.00
W3	124998	125002	-4	-1.14	-0.00
W4	62497	62503	-6	-1.64	-0.00
W5	31247	31252	-5	-1.53	-0.00
W6	15622	15629	-7	-1.85	-0.00
W7	7811	7817	-6	-1.72	-0.00
**W8**	**3906**	**3933**	**-27**	**-3.67**	**-0.01**
W9	1953	1951	2	1.01	0.00
W10	981	976	5	1.58	0.01
W11	490	492	-2	-1.00	-0.00
W12	246	241	5	1.58	0.02
W13	122	126	-4	-1.41	-0.03
W14	58	60	-2	-1.00	-0.03
W15	28	30	-2	-1.00	-0.07
W16	19	14	5	1.58	0.31
W17	6	5	1	0.71	0.18
W18	0	5	-5	-1.52	-Inf
W19	6	0	6	1.67	Inf

Although the corpus size is larger, LORIDP z-scores still treat the difference in frequency between W0 and W8 as significant. Corpus size influences the unmodified log-odds ratios, which are are smaller than in the illustrations in in Tables A, B, and C.
